# Supplementary material for: Genomic signatures associated with maintenance of genome stability and venom turnover in two parasitoid wasps
Source: Nat Commun. 2022 Oct 27;13:6417. doi: 10.1038/s41467-022-34202-y (PMC9613689; doi:10.1038/s41467-022-34202-y)
Supplement: Supplementary file 1 — Supplementary information [file 41467_2022_34202_MOESM1_ESM.pdf]

Supplementary Information

Genomic signatures associated with maintenance of genome stability and venom turnover in two parasitoid wasps

Xinhai Ye, Yi Yang, Can Zhao, Shan Xiao, Yu H. Sun, Chun He, Shijiao Xiong, Xianxin Zhao, Bo Zhang, Haiwei Lin, Jiamin Shi, Yang Mei, Hongxing Xu, Qi Fang, Fei Wu, Dunsong Li, Gongyin Ye

Correspondence:
Gongyin Ye, chu@zju.edu.cn; DunSong Li, dsli@gdppri.cn

Xinhai Ye, Yi Yang and Can Zhao contribute equally.

This PDF file includes:
Supplementary Fig. 1-85
Supplementary Table 1-22

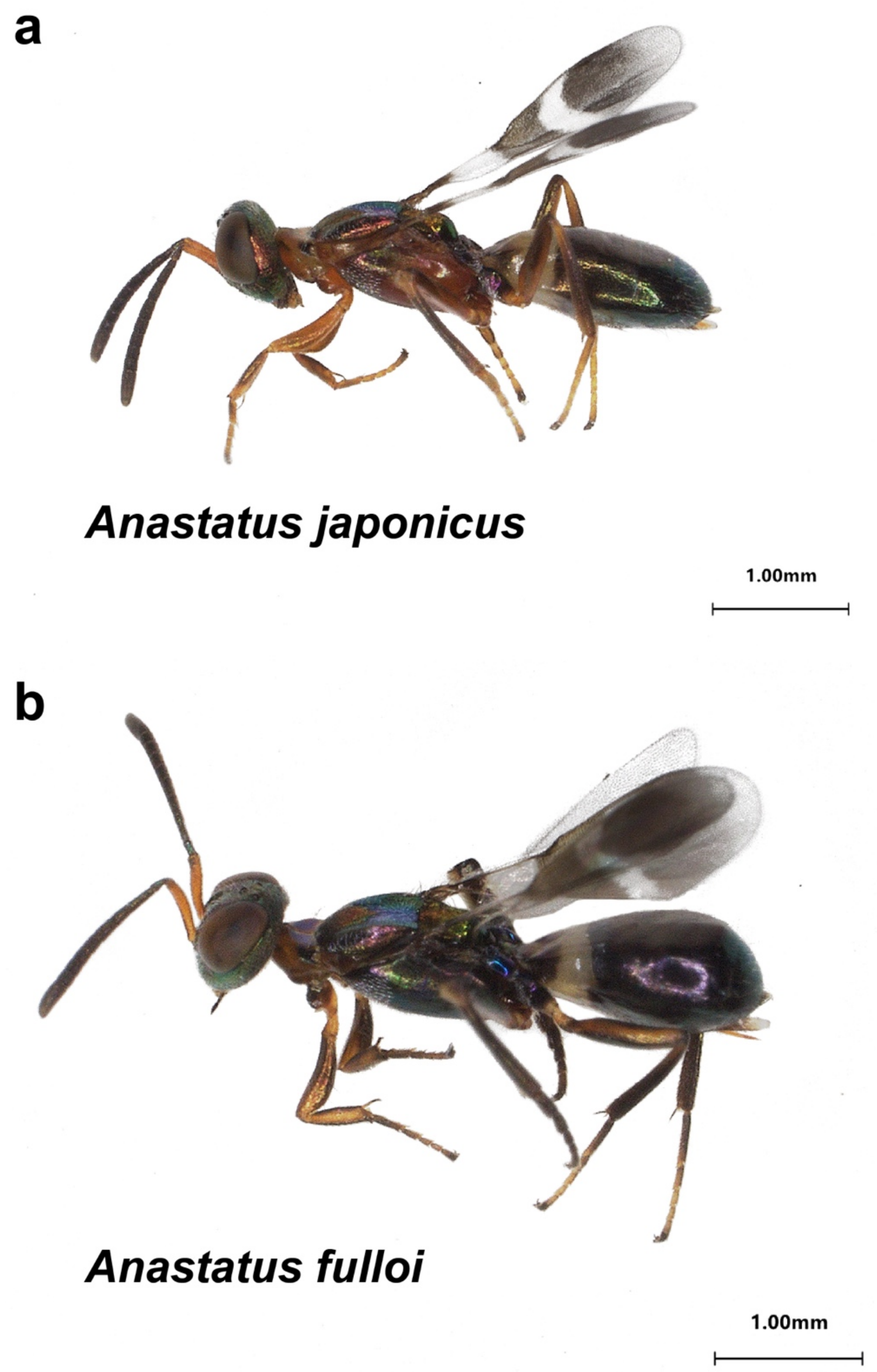

Supplementary Fig. 1 | The pictures of the two *Anastatus* wasps we sequenced in this study. a, The lateral habitus of a female *A. japonicus*. **b**, The lateral habitus of a female *A. fulloi*. The photos were taken by the digital microscope VHX-7100 (KEYENCE). Scale bars: 1.00 mm.

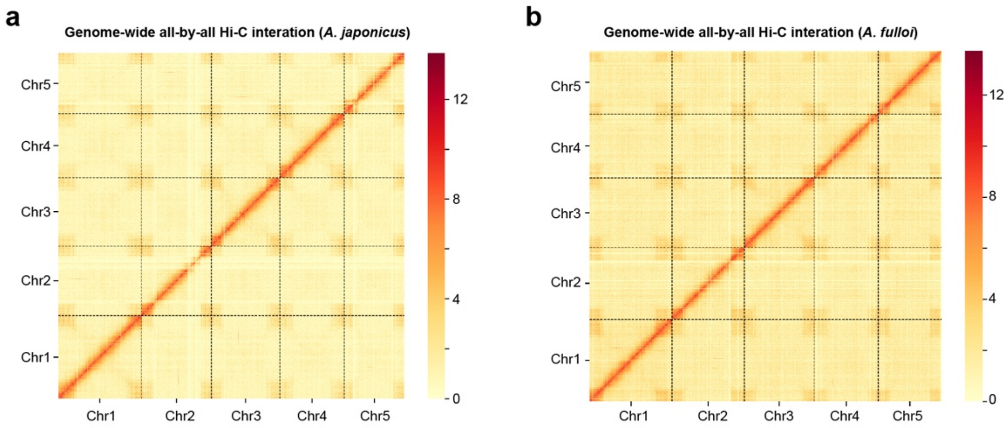

Supplementary Fig. 2 | Heatmap of Hi-C interactions among all chromosomes of *A. japonicus* (a) and *A. fulloi* (b).

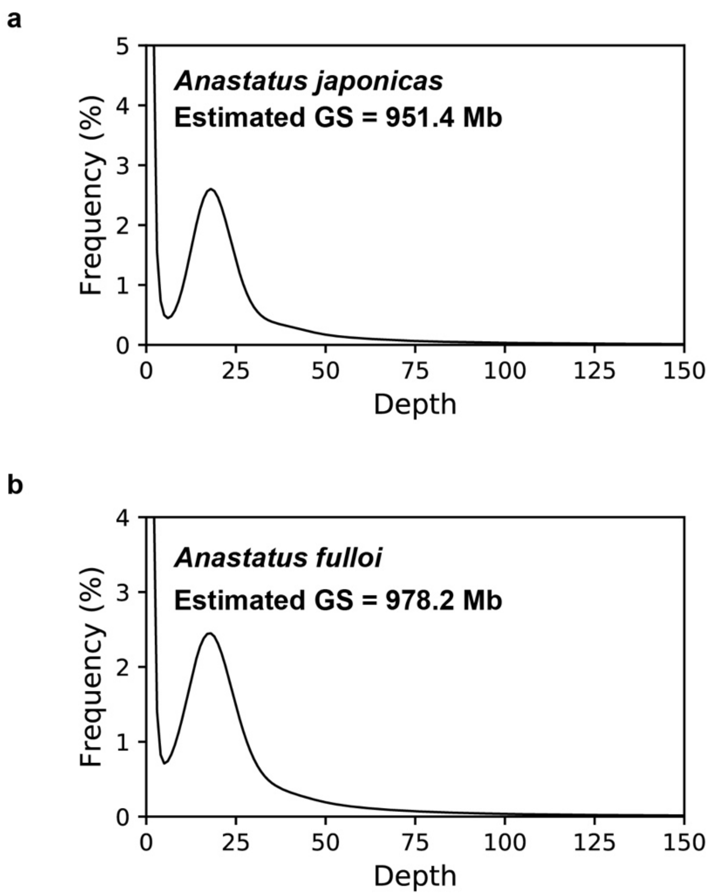

Supplementary Fig. 3 | *K*-mer analyses of *A. japonicus* (a) and *A. fulloi* (b).

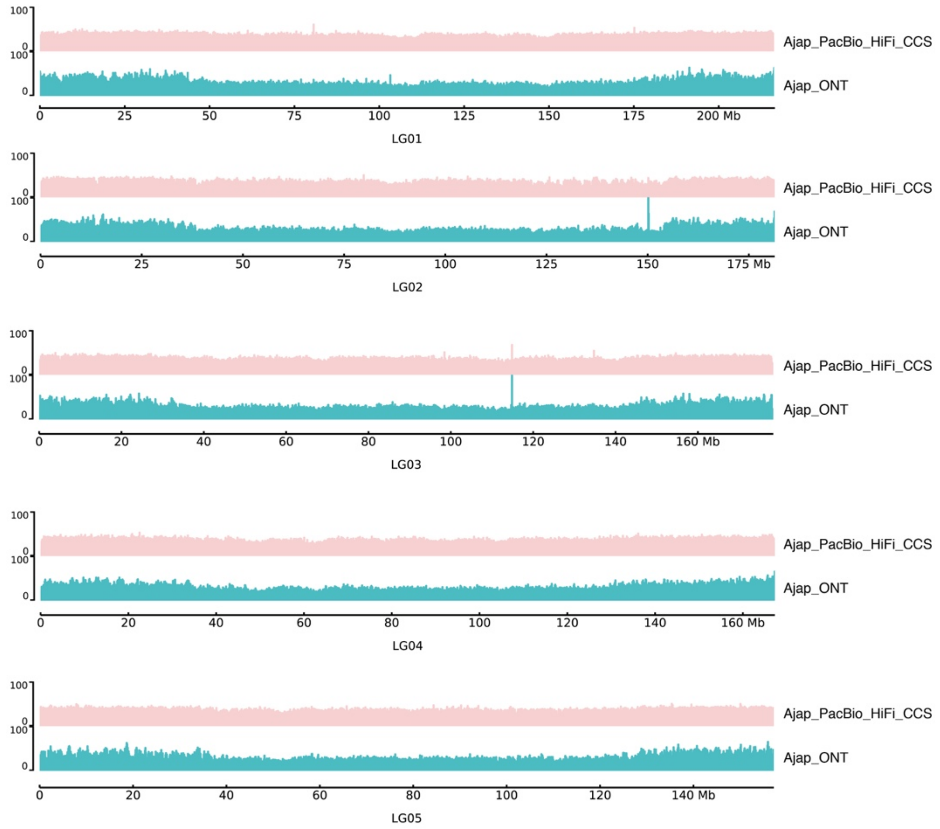

Supplementary Fig. 4 | Sequencing coverage of *A. japonicus*. Chromosome-level genome coverage of mapped HiFi and ONT reads is shown with primary alignments.

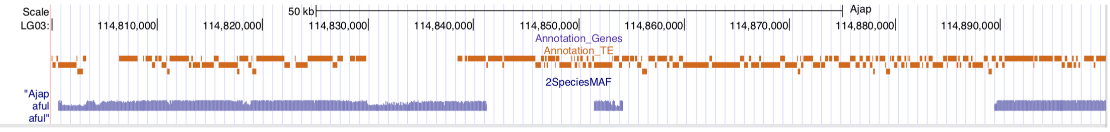

Supplementary Fig. 5 | UCSC genome browser view of a region with increased coverage supported by HiFi and ONT in *A. japonicus* genome (LG03:114,800,000-114,900,000).

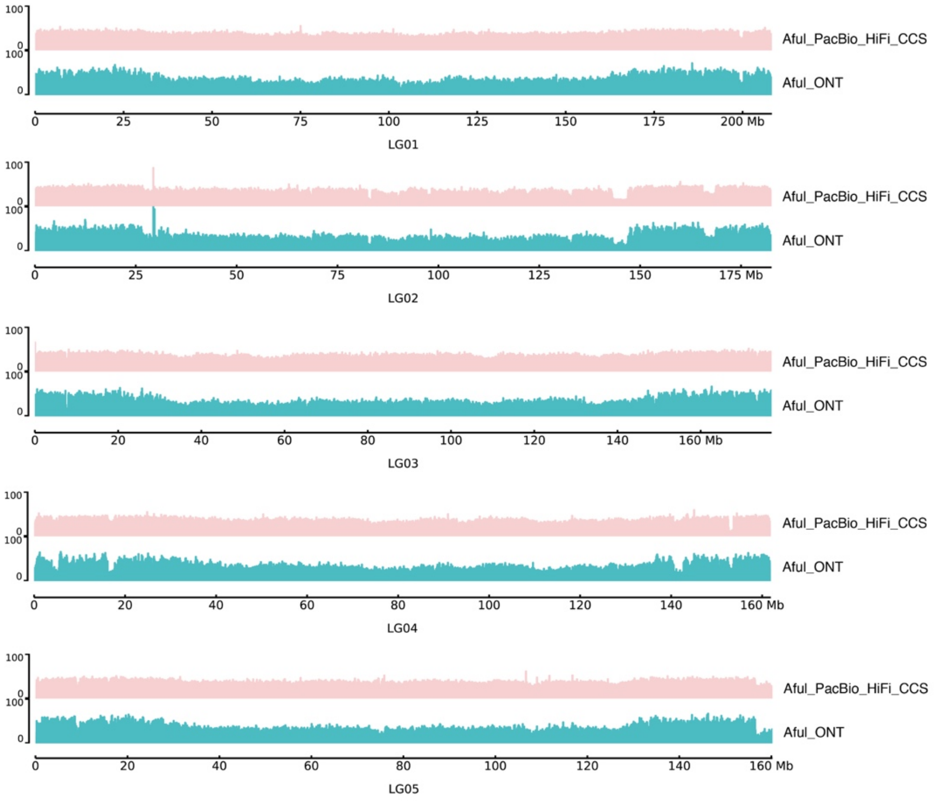

Supplementary Fig. 6 | Sequencing coverage of *A. fulloi*. Chromosome-level genome coverage of mapped HiFi and ONT reads is shown with primary alignments.

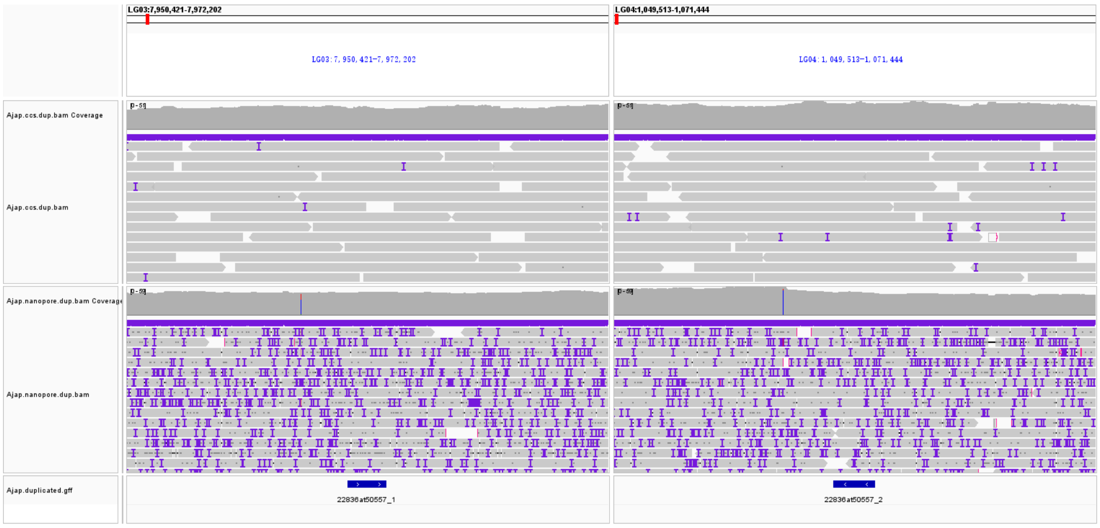

Supplementary Fig. 7 | IGV browser view of PacBio HiFi and ONT reads mapping on the duplicated BUSCO gene (geneid: 22836at50557) in *A. japonicus*.

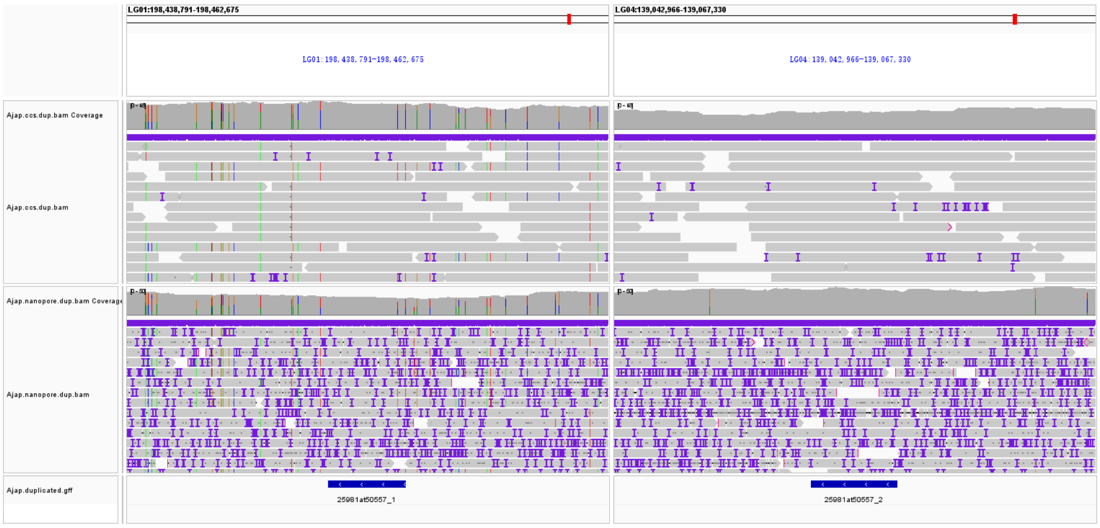

Supplementary Fig. 8 | IGV browser view of PacBio HiFi and ONT reads mapping on the duplicated BUSCO gene (geneid: 25981at50557) in *A. japonicus*.

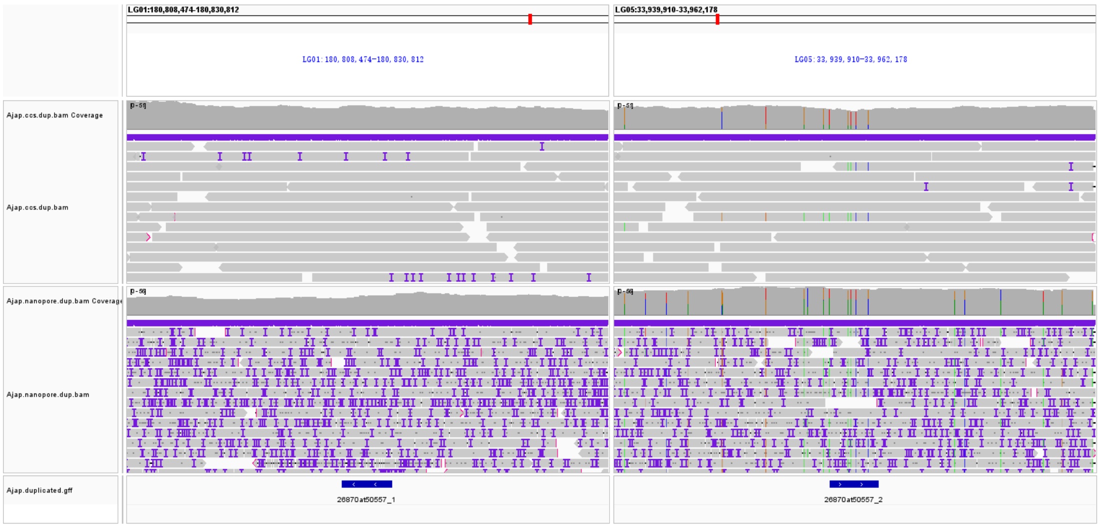

Supplementary Fig. 9 | IGV browser view of PacBio HiFi and ONT reads mapping on the duplicated BUSCO gene (geneid: 26870at50557) in *A. japonicus*.

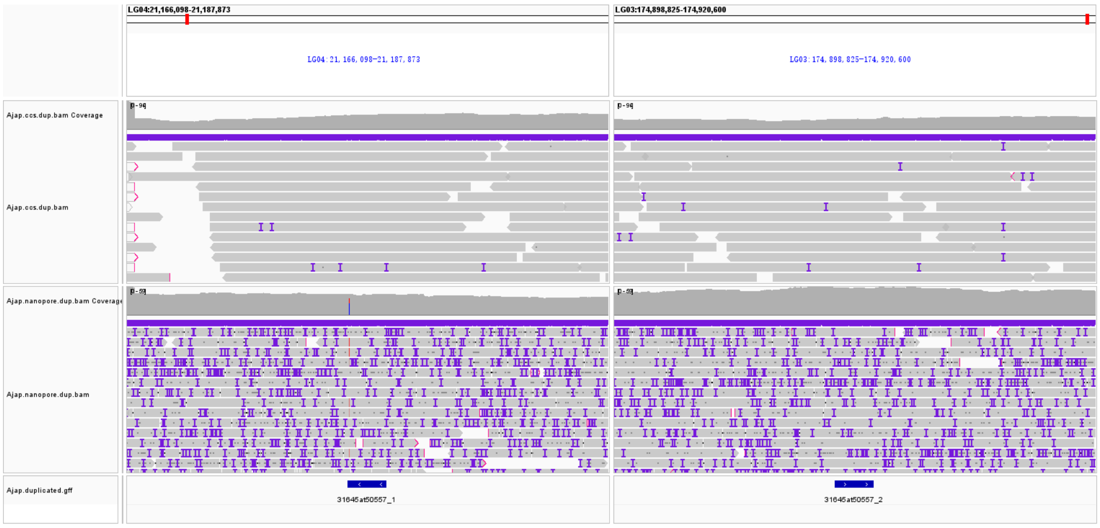

Supplementary Fig. 10 | IGV browser view of PacBio HiFi and ONT reads mapping on the duplicated BUSCO gene (geneid: 31645at50557) in *A. japonicus*.

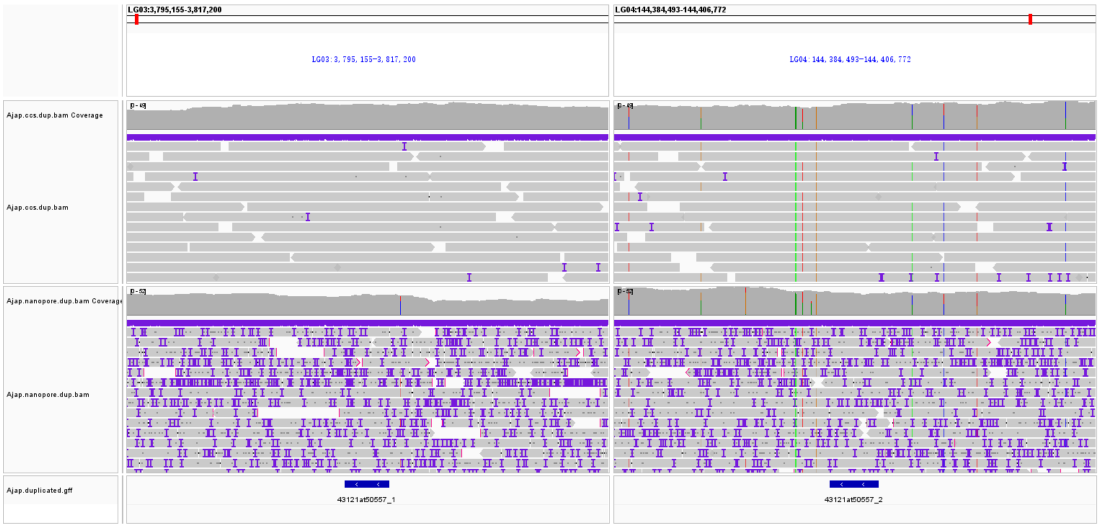

Supplementary Fig. 11 | IGV browser view of PacBio HiFi and ONT reads mapping on the duplicated BUSCO gene (geneid: 43121at50557) in *A. japonicus.*

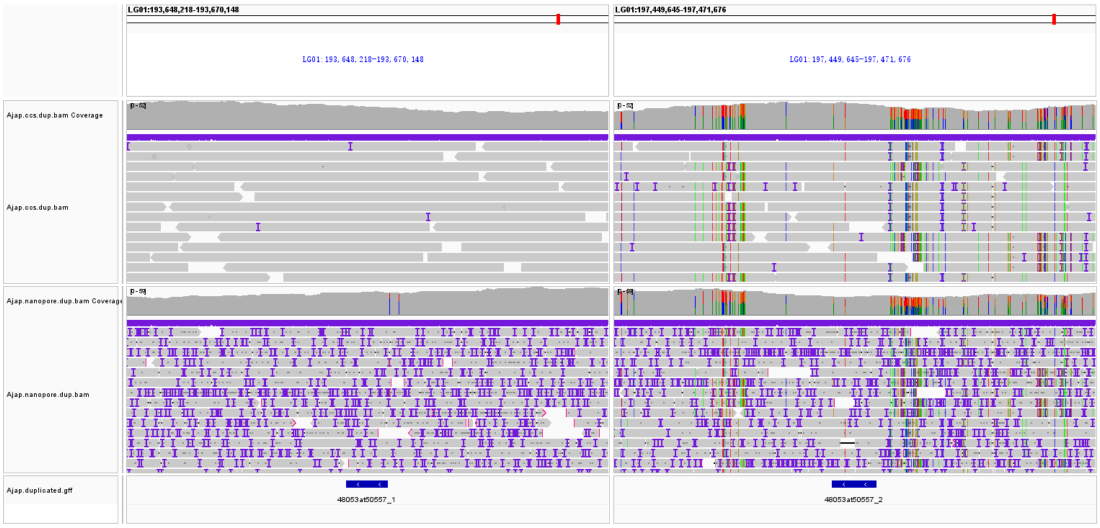

Supplementary Fig. 12 | IGV browser view of PacBio HiFi and ONT reads mapping on the duplicated BUSCO gene (geneid: 48053at50557) in *A. japonicus.*

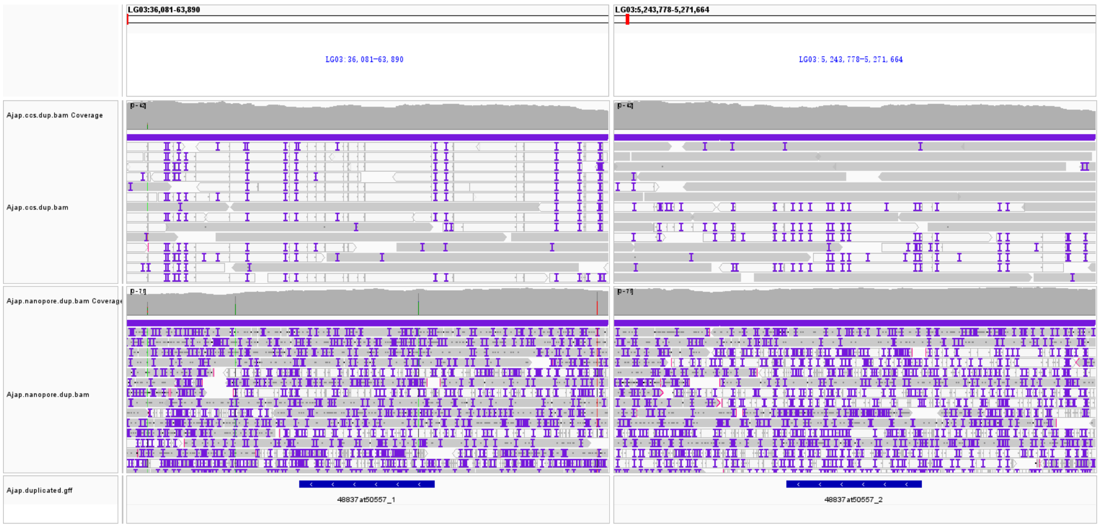

Supplementary Fig. 13 | IGV browser view of PacBio HiFi and ONT reads mapping on the duplicated BUSCO gene (geneid: 48837at50557) in *A. japonicus.*

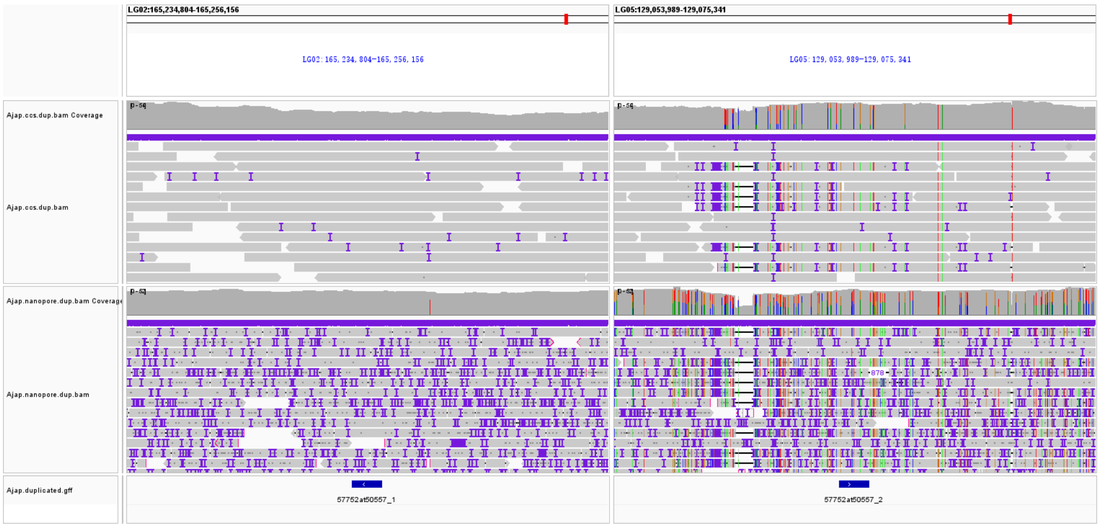

Supplementary Fig. 14 | IGV browser view of PacBio HiFi and ONT reads mapping on the duplicated BUSCO gene (geneid: 57752at50557) in *A. japonicus.*

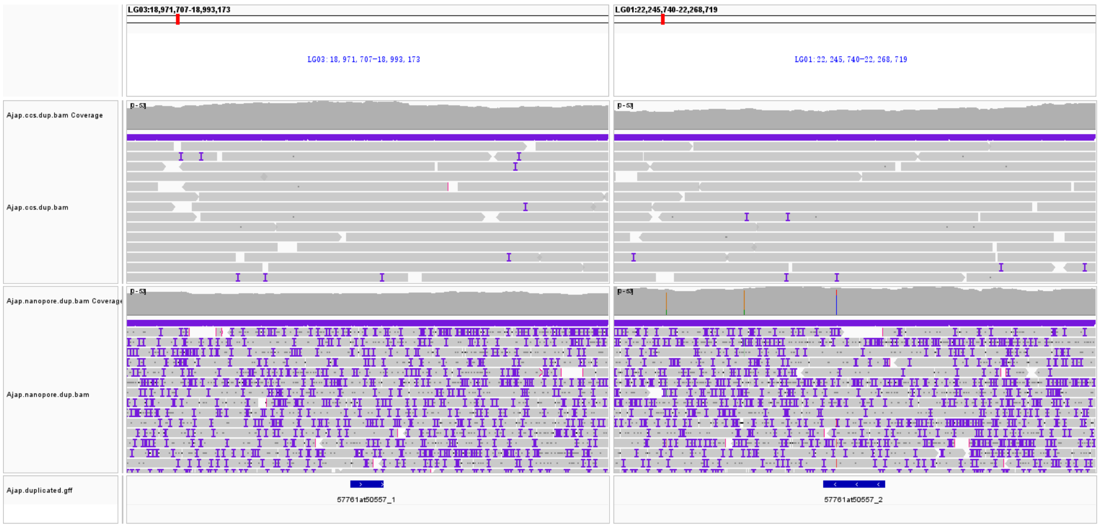

Supplementary Fig. 15 | IGV browser view of PacBio HiFi and ONT reads mapping on the duplicated BUSCO gene (geneid: 57761at50557) in *A. japonicus.*

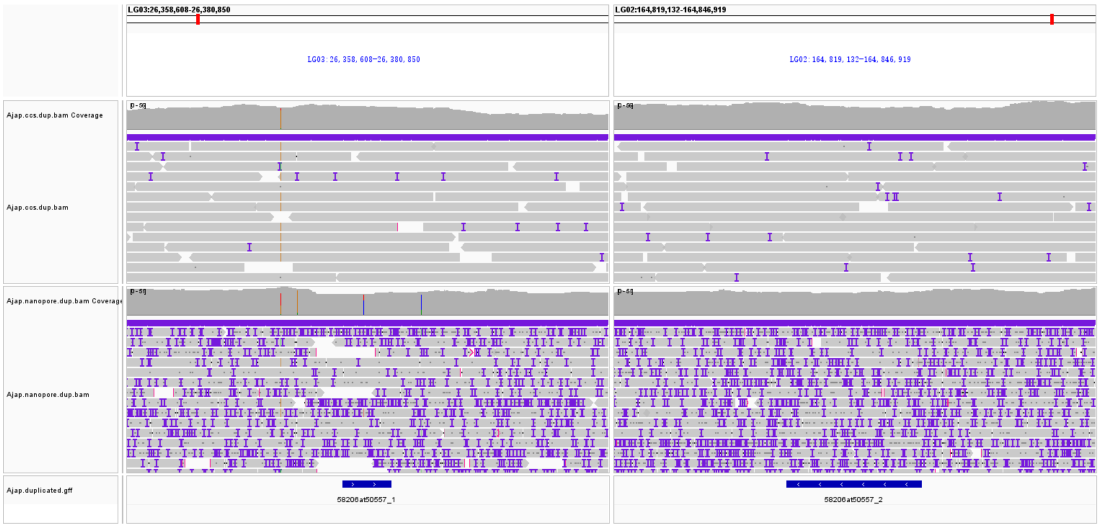

Supplementary Fig. 16 | IGV browser view of PacBio HiFi and ONT reads mapping on the duplicated BUSCO gene (geneid: 58206at50557) in *A. japonicus.*

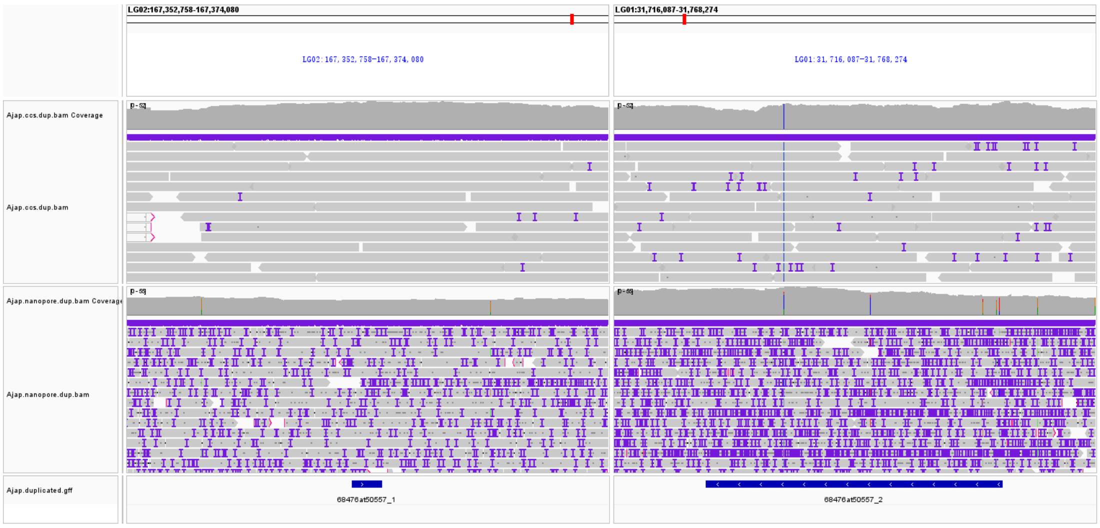

Supplementary Fig. 17 | IGV browser view of PacBio HiFi and ONT reads mapping on the duplicated BUSCO gene (geneid: 68476at50557) in *A. japonicus.*

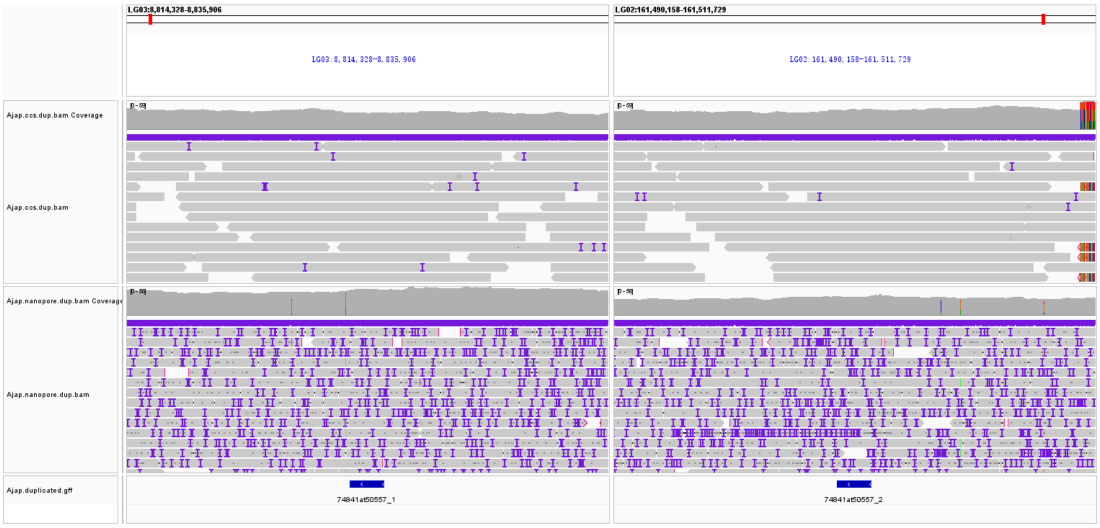

Supplementary Fig. 18 | IGV browser view of PacBio HiFi and ONT reads mapping on the duplicated BUSCO gene (geneid: 74841at50557) in *A. japonicus.*

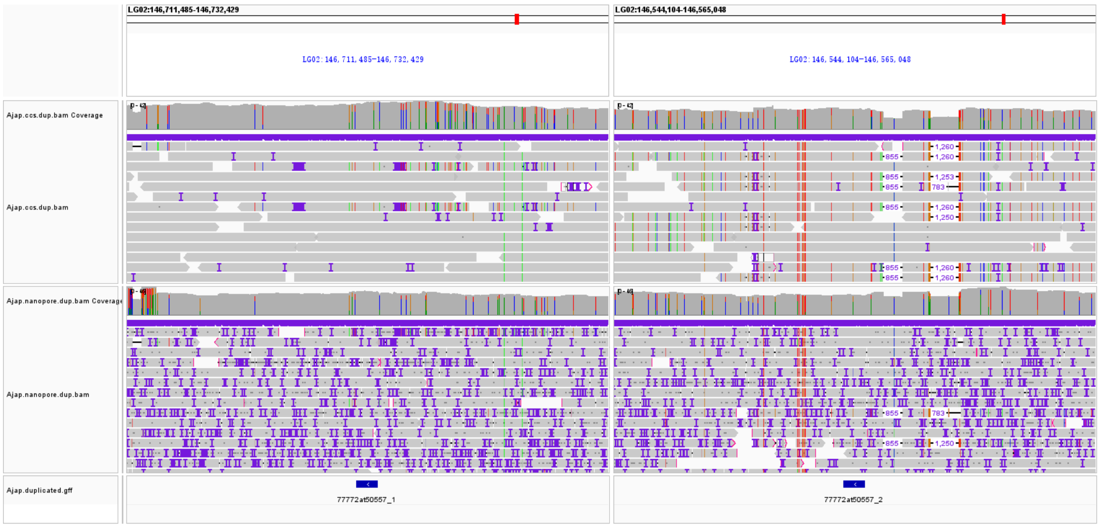

Supplementary Fig. 19 | IGV browser view of PacBio HiFi and ONT reads mapping on the duplicated BUSCO gene (geneid: 77772at50557) in *A. japonicus.*

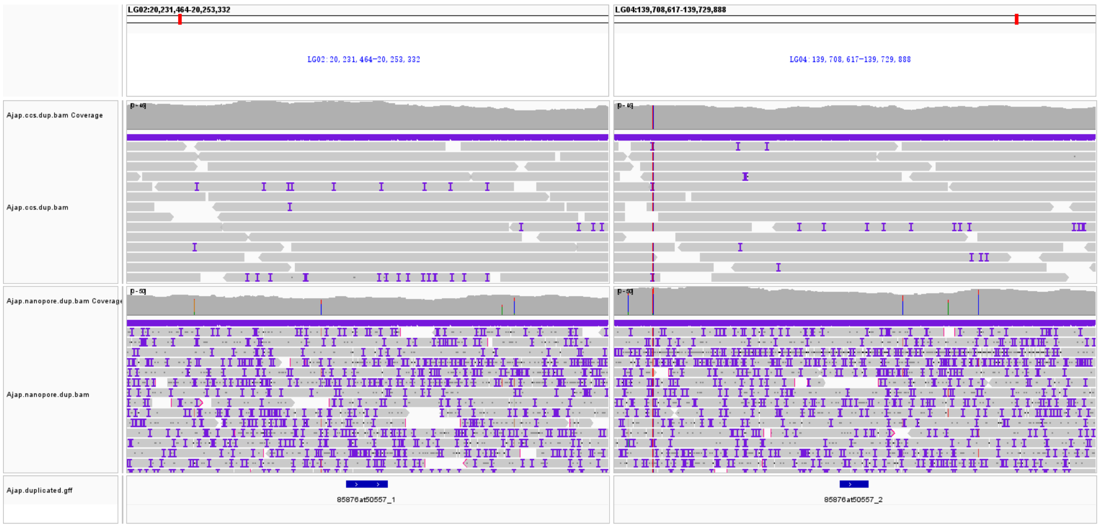

Supplementary Fig. 20 | IGV browser view of PacBio HiFi and ONT reads mapping on the duplicated BUSCO gene (geneid: 85876at50557) in *A. japonicus.*

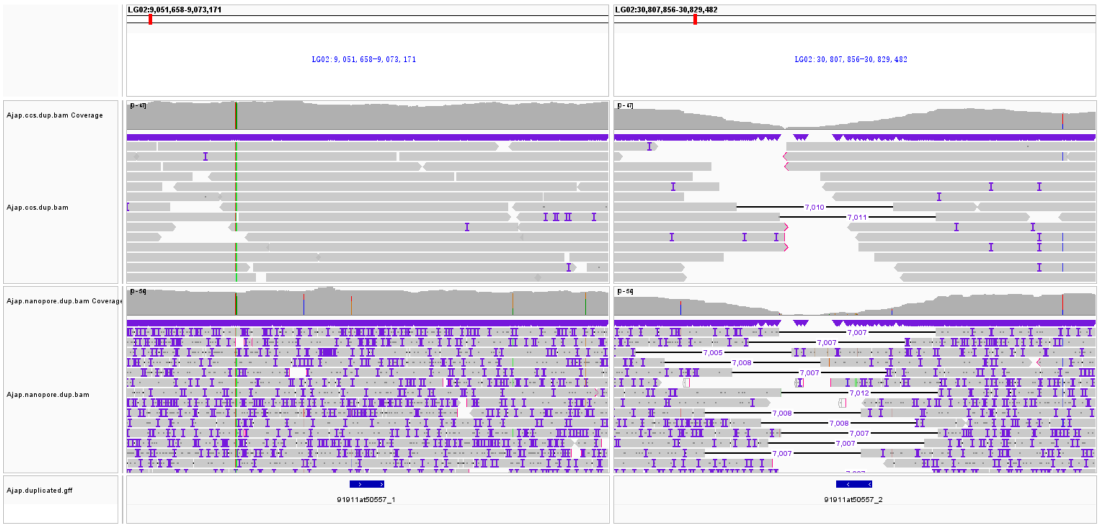

Supplementary Fig. 21 | IGV browser view of PacBio HiFi and ONT reads mapping on the duplicated BUSCO gene (geneid: 91911at50557) in *A. japonicus.*

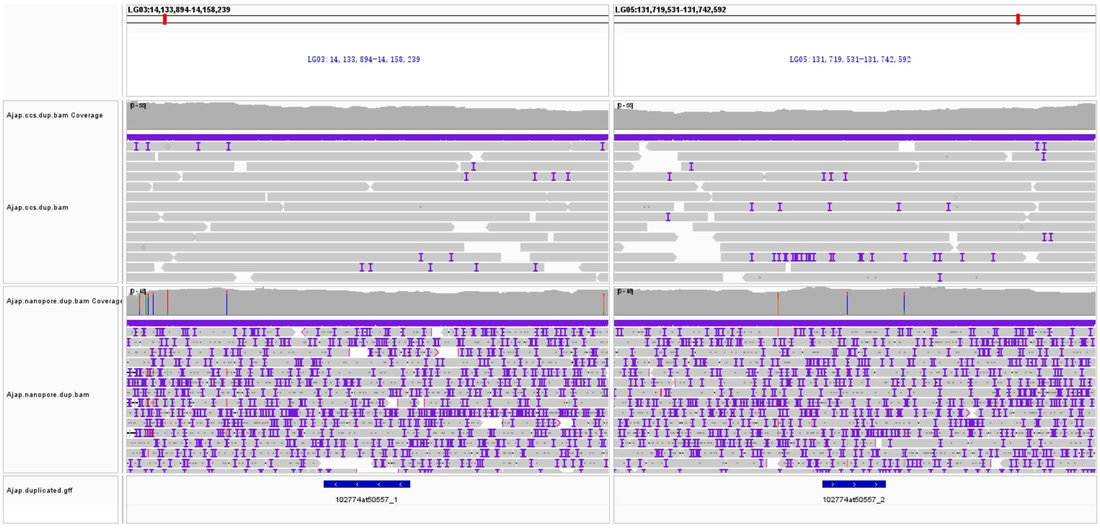

Supplementary Fig. 22 | IGV browser view of PacBio HiFi and ONT reads mapping on the duplicated BUSCO gene (geneid: 102774at50557) in *A. japonicus.*

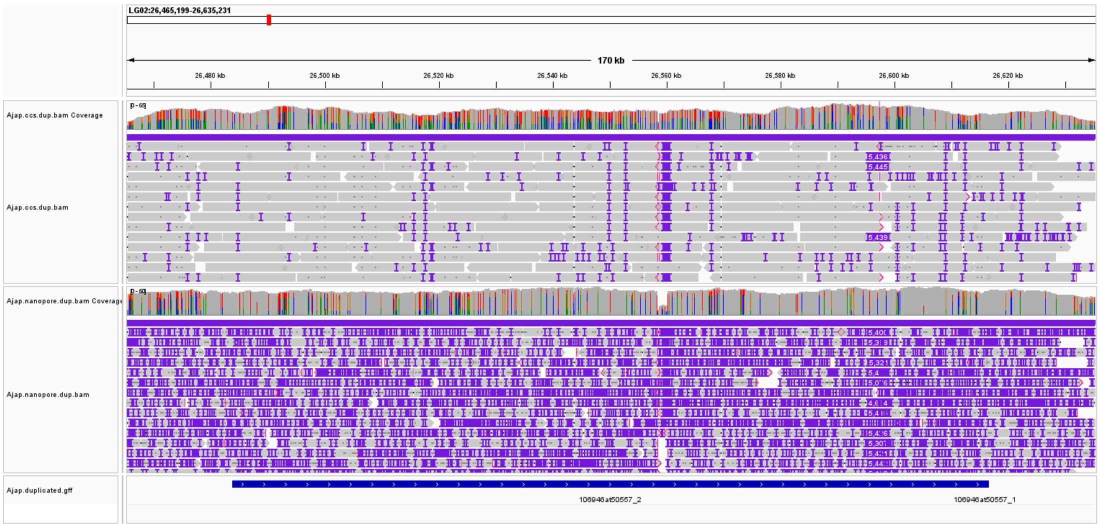

Supplementary Fig. 23 | IGV browser view of PacBio HiFi and ONT reads mapping on the duplicated BUSCO gene (geneid: 106946at50557) in *A. japonicus.*

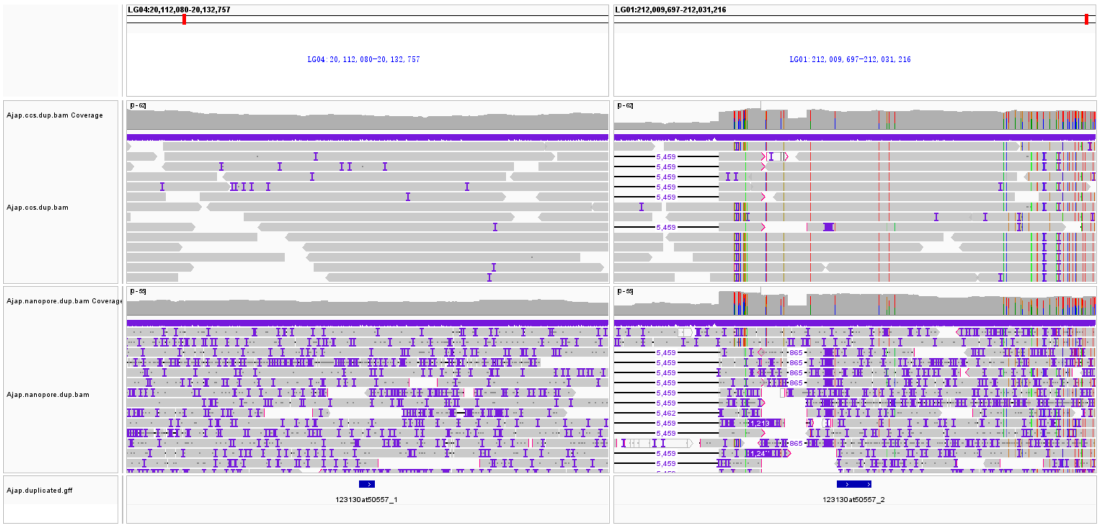

Supplementary Fig. 24 | IGV browser view of PacBio HiFi and ONT reads mapping on the duplicated BUSCO gene (geneid: 123130at50557) in *A. japonicus.*

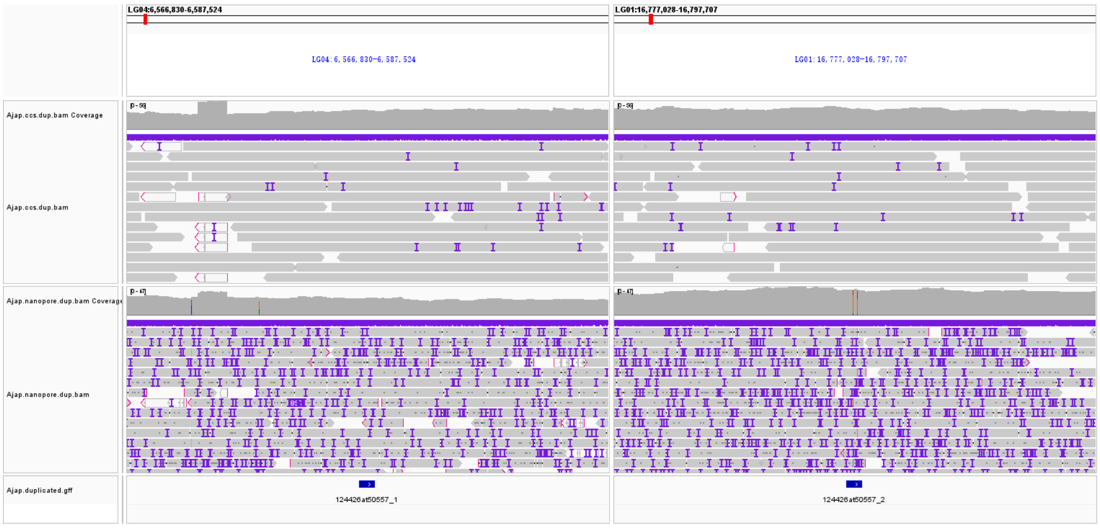

Supplementary Fig. 25 | IGV browser view of PacBio HiFi and ONT reads mapping on the duplicated BUSCO gene (geneid: 124426at50557) in *A. japonicus.*

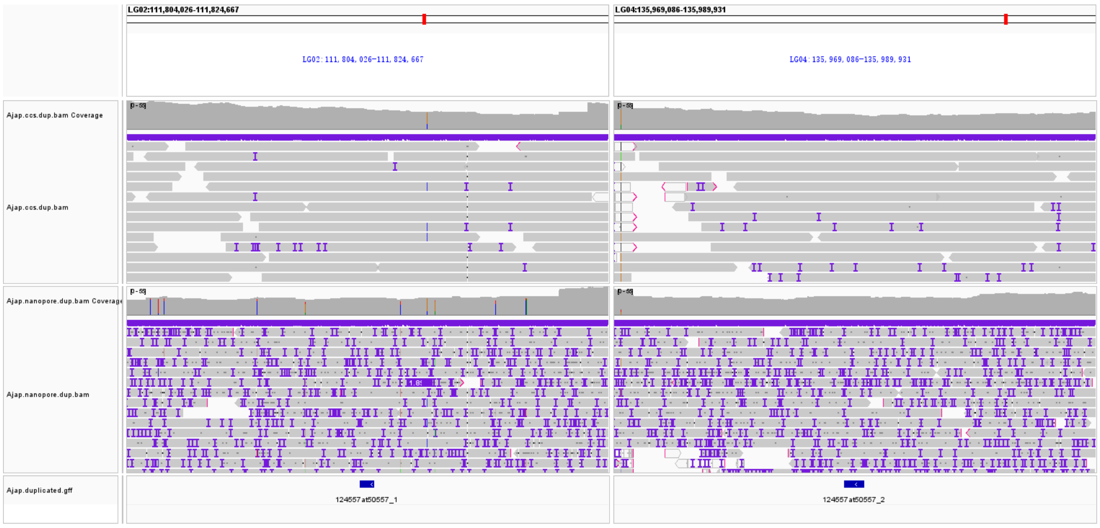

Supplementary Fig. 26 | IGV browser view of PacBio HiFi and ONT reads mapping on the duplicated BUSCO gene (geneid: 124557at50557) in *A. japonicus.*

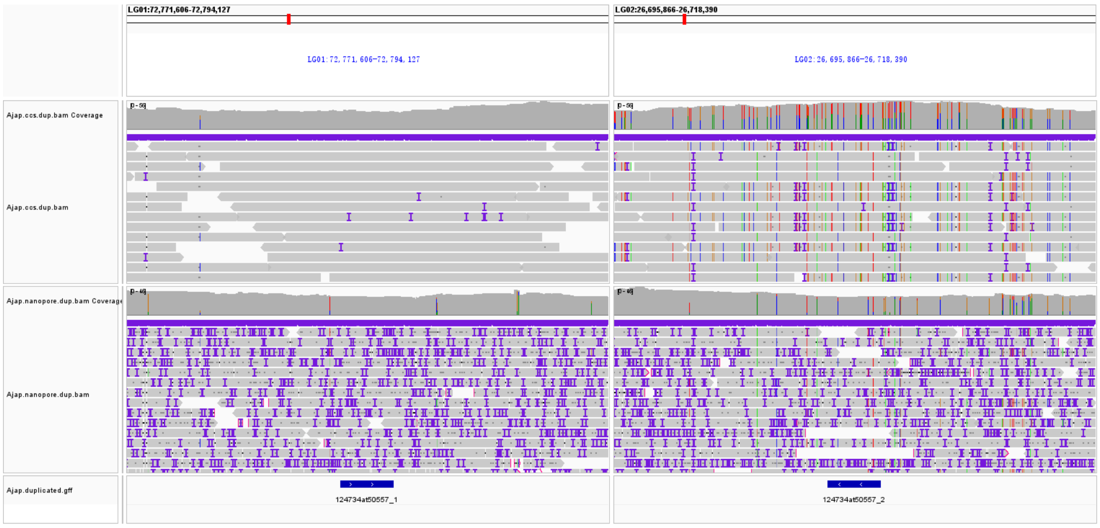

Supplementary Fig. 27 | IGV browser view of PacBio HiFi and ONT reads mapping on the duplicated BUSCO gene (geneid: 124734at50557) in *A. japonicus.*

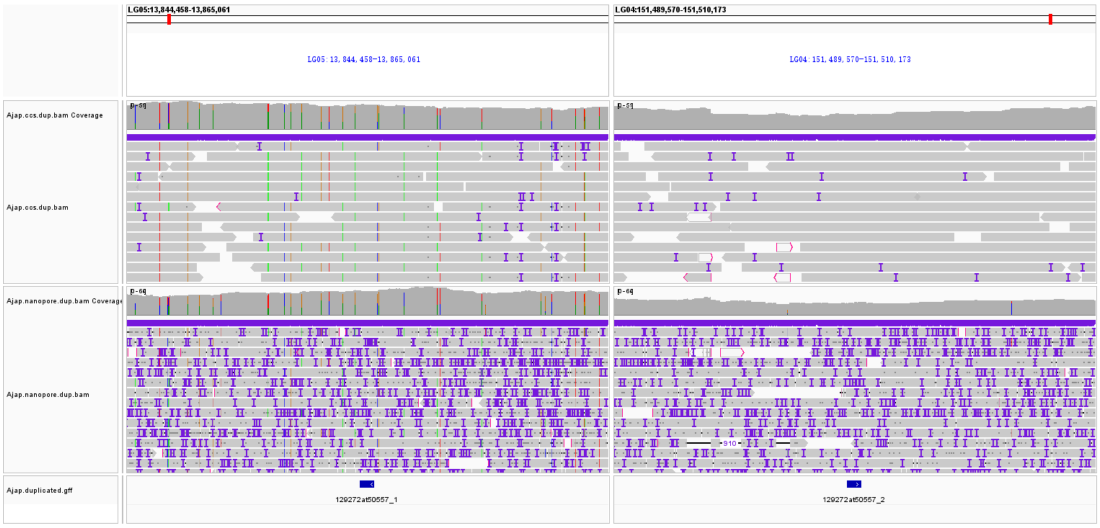

Supplementary Fig. 28 | IGV browser view of PacBio HiFi and ONT reads mapping on the duplicated BUSCO gene (geneid: 129272at50557) in *A. japonicus.*

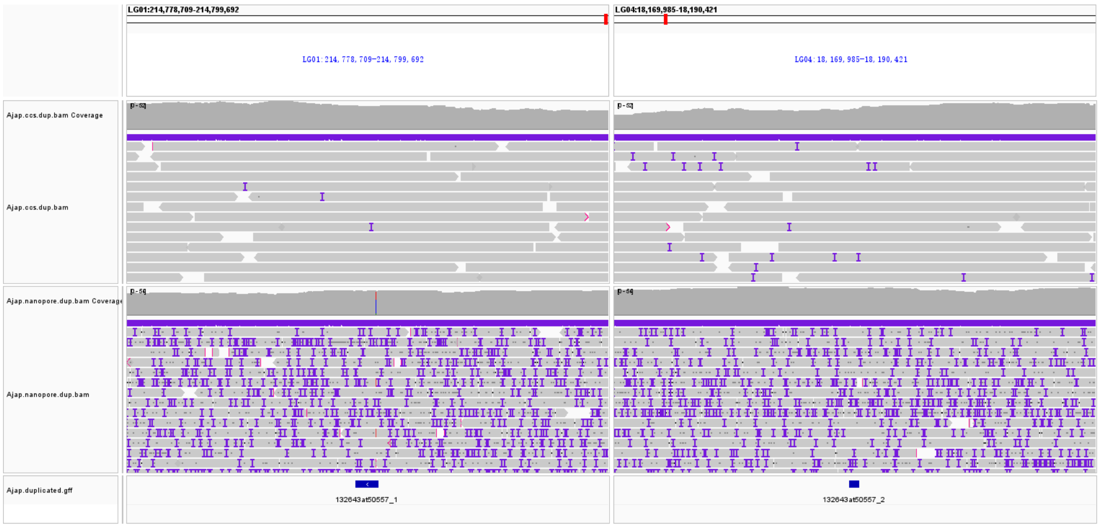

Supplementary Fig. 29 | IGV browser view of PacBio HiFi and ONT reads mapping on the duplicated BUSCO gene (geneid: 132643at50557) in *A. japonicus.*

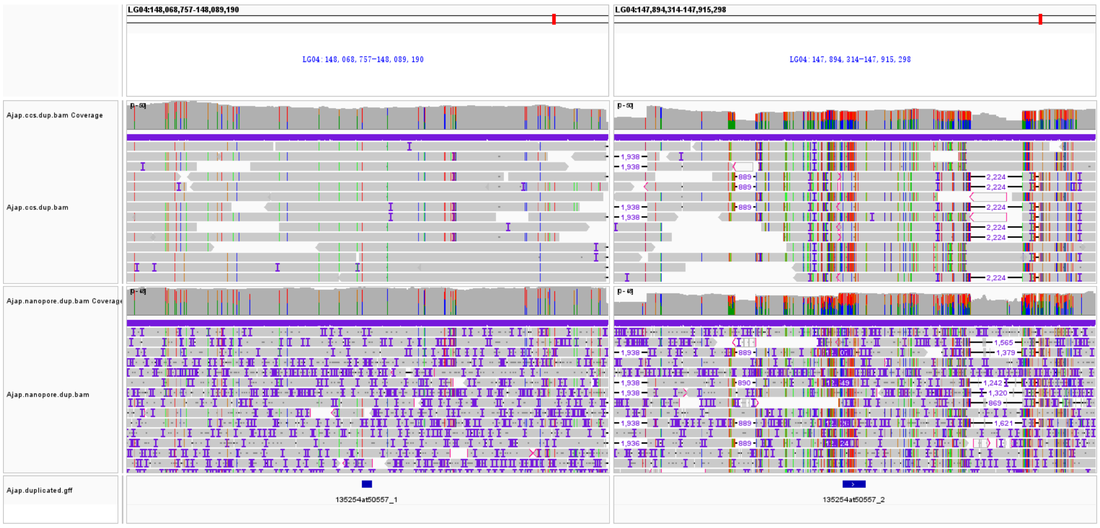

Supplementary Fig. 30 | IGV browser view of PacBio HiFi and ONT reads mapping on the duplicated BUSCO gene (geneid: 135254at50557) in *A. japonicus.*

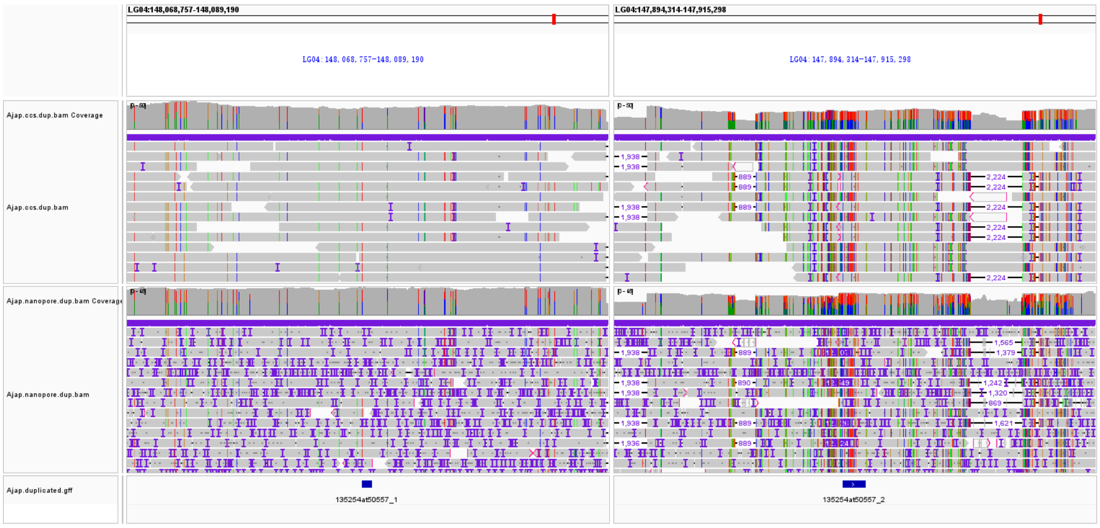

Supplementary Fig. 31 | IGV browser view of PacBio HiFi and ONT reads mapping on the duplicated BUSCO gene (geneid: 137402at50557) in *A. japonicus.*

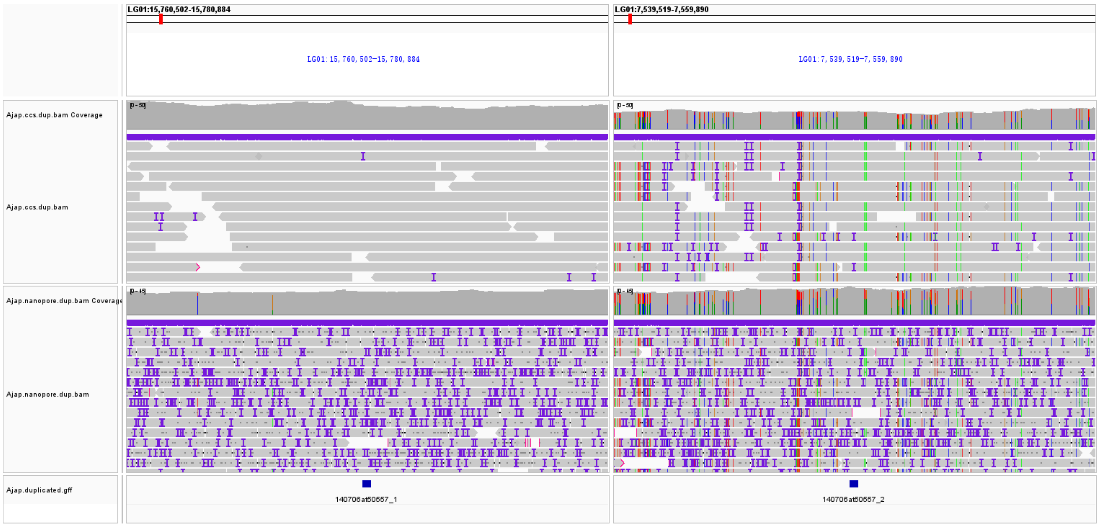

Supplementary Fig. 32 | IGV browser view of PacBio HiFi and ONT reads mapping on the duplicated BUSCO gene (geneid: 140706at50557) in *A. japonicus.*

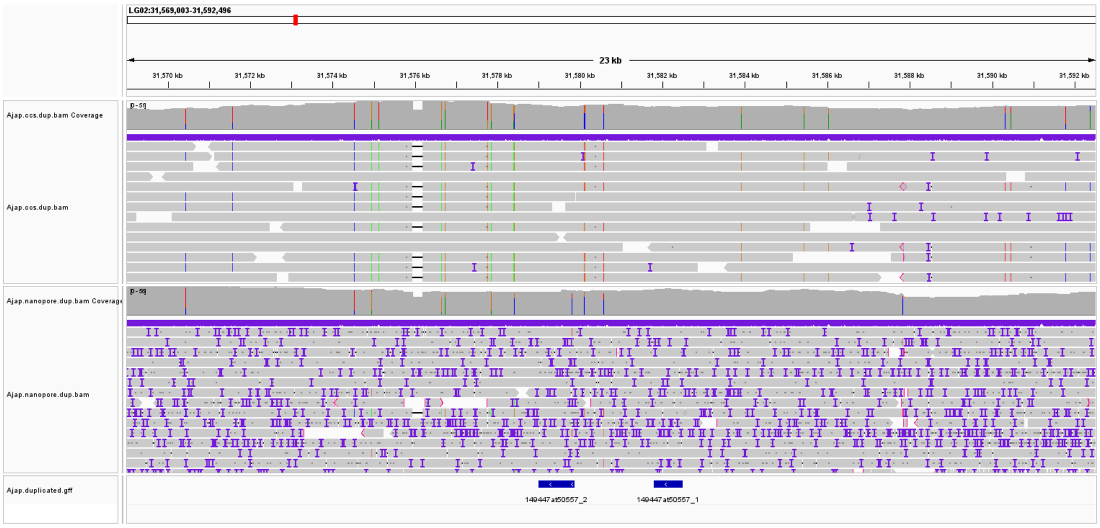

Supplementary Fig. 33 | IGV browser view of PacBio HiFi and ONT reads mapping on the duplicated BUSCO gene (geneid: 149447at50557) in *A. japonicus.*

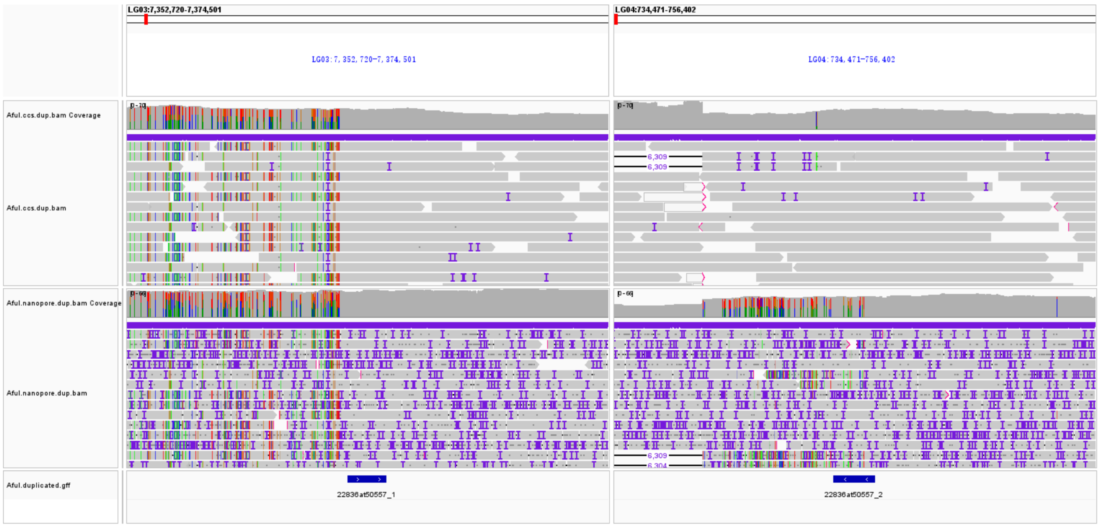

Supplementary Fig. 34 | IGV browser view of PacBio HiFi and ONT reads mapping on the duplicated BUSCO gene (geneid: 22836at50557) in *A. fulloi.*

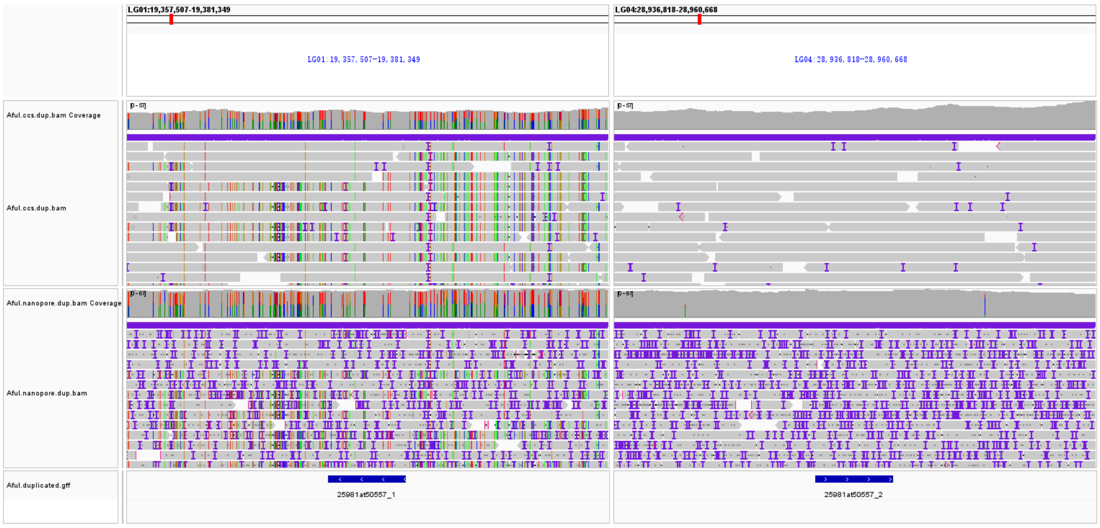

Supplementary Fig. 35 | IGV browser view of PacBio HiFi and ONT reads mapping on the duplicated BUSCO gene (geneid: 25981at50557) in *A. fulloi.*

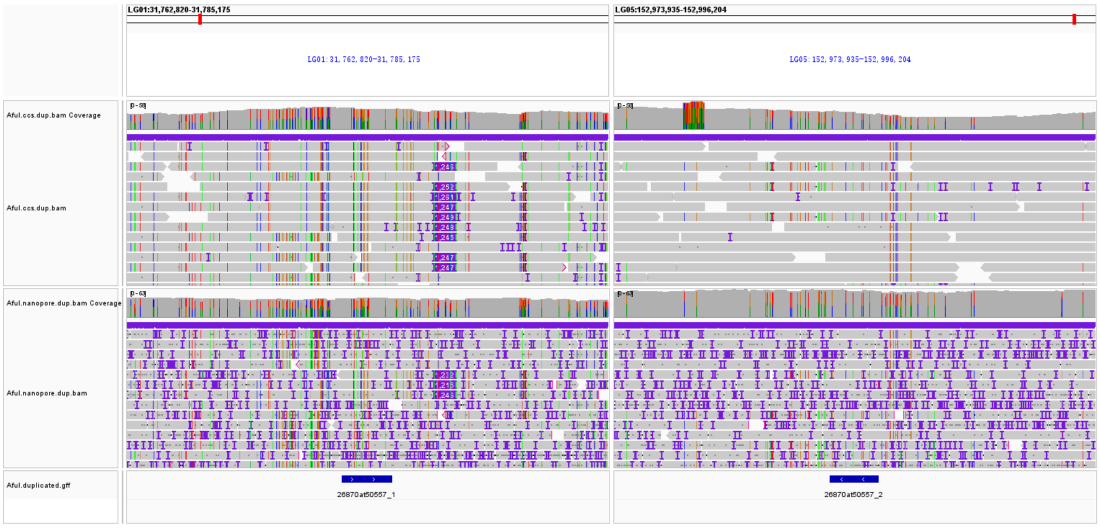

Supplementary Fig. 36 | IGV browser view of PacBio HiFi and ONT reads mapping on the duplicated BUSCO gene (geneid: 26870at50557) in *A. fulloi.*

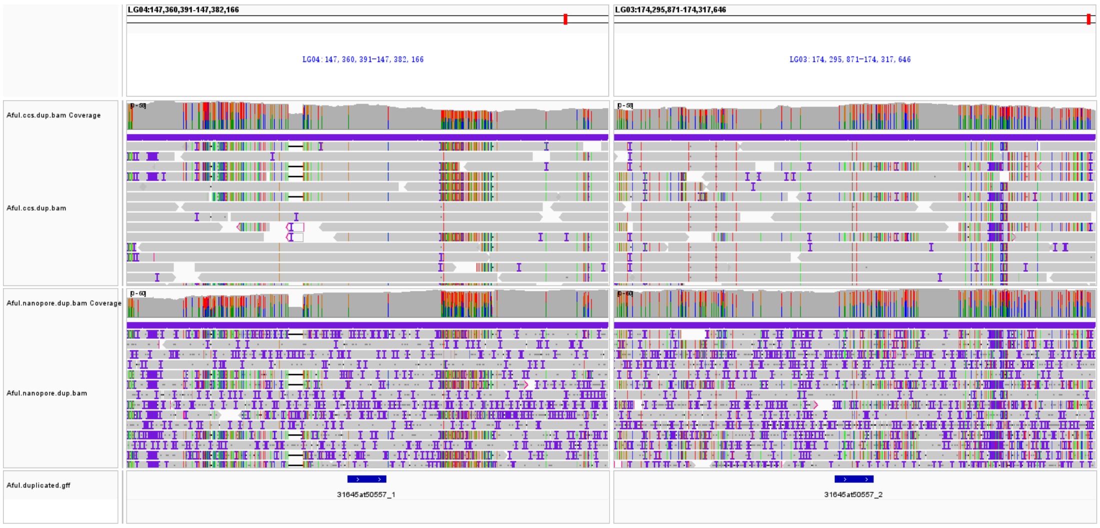

Supplementary Fig. 37 | IGV browser view of PacBio HiFi and ONT reads mapping on the duplicated BUSCO gene (geneid: 31645at50557) in *A. fulloi.*

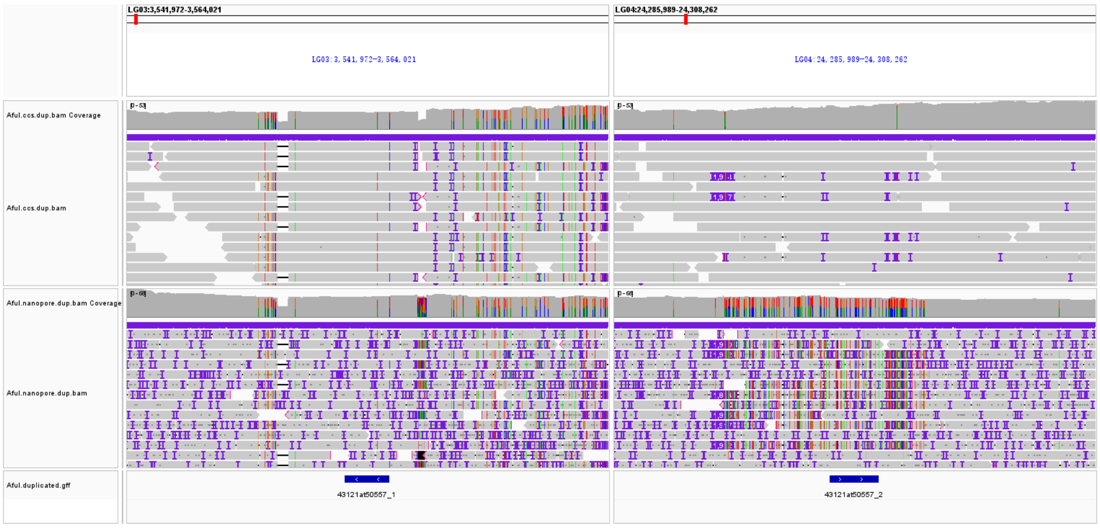

Supplementary Fig. 38 | IGV browser view of PacBio HiFi and ONT reads mapping on the duplicated BUSCO gene (geneid: 43121at50557) in *A. fulloi.*

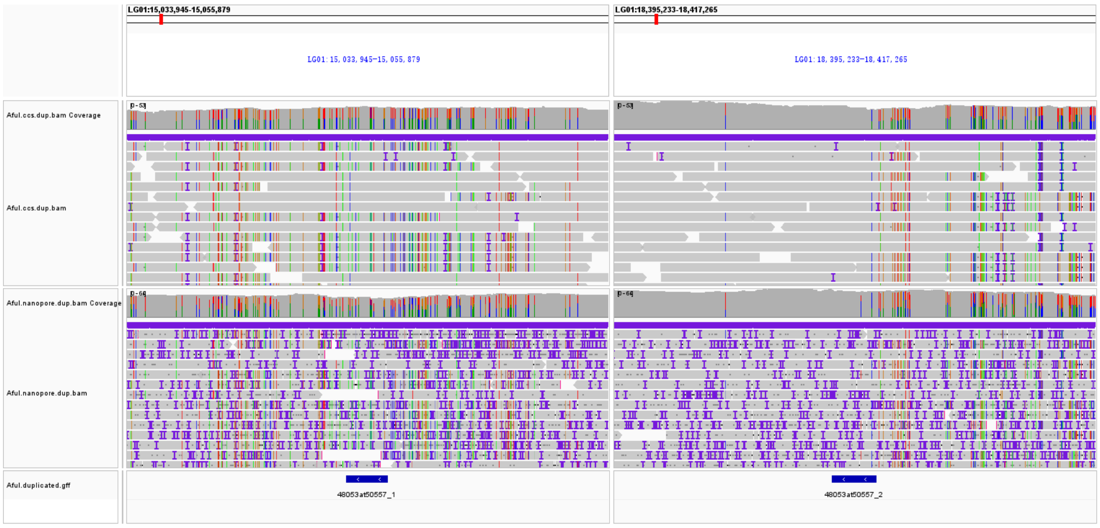

Supplementary Fig. 39 | IGV browser view of PacBio HiFi and ONT reads mapping on the duplicated BUSCO gene (geneid: 48053at50557) in *A. fulloi.*

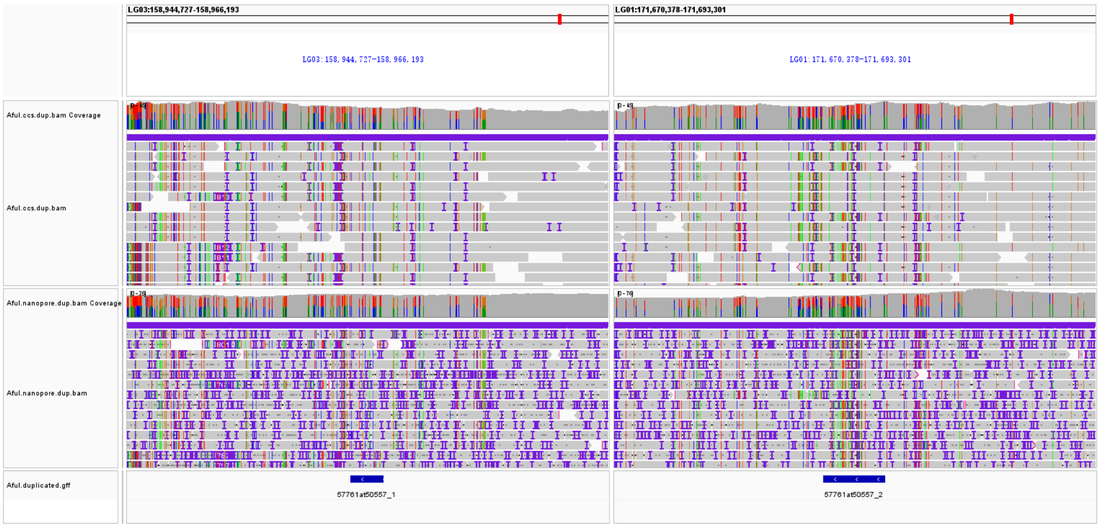

Supplementary Fig. 40 | IGV browser view of PacBio HiFi and ONT reads mapping on the duplicated BUSCO gene (geneid: 57761at50557) in *A. fulloi.*

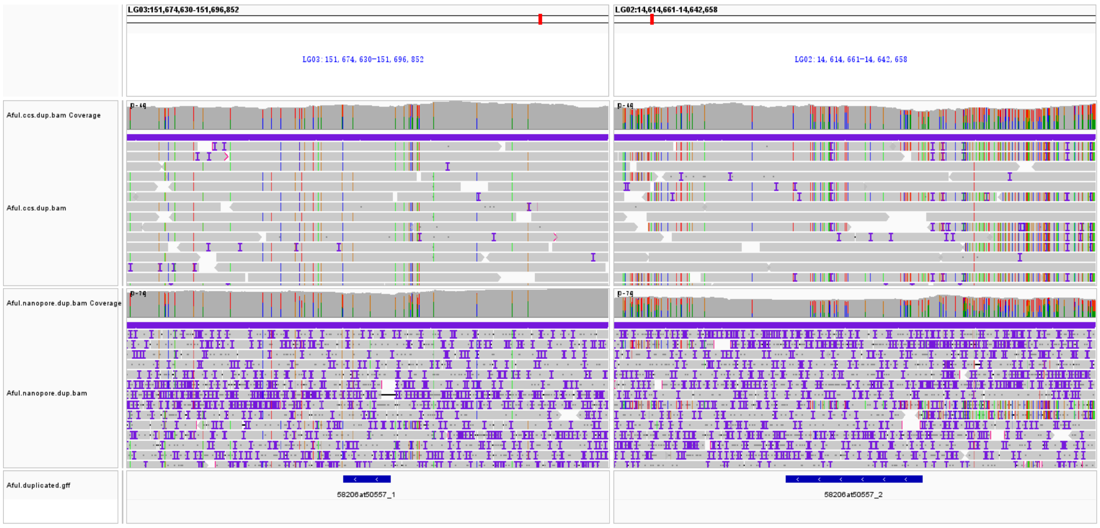

Supplementary Fig. 41 | IGV browser view of PacBio HiFi and ONT reads mapping on the duplicated BUSCO gene (geneid: 58206at50557) in *A. fulloi.*

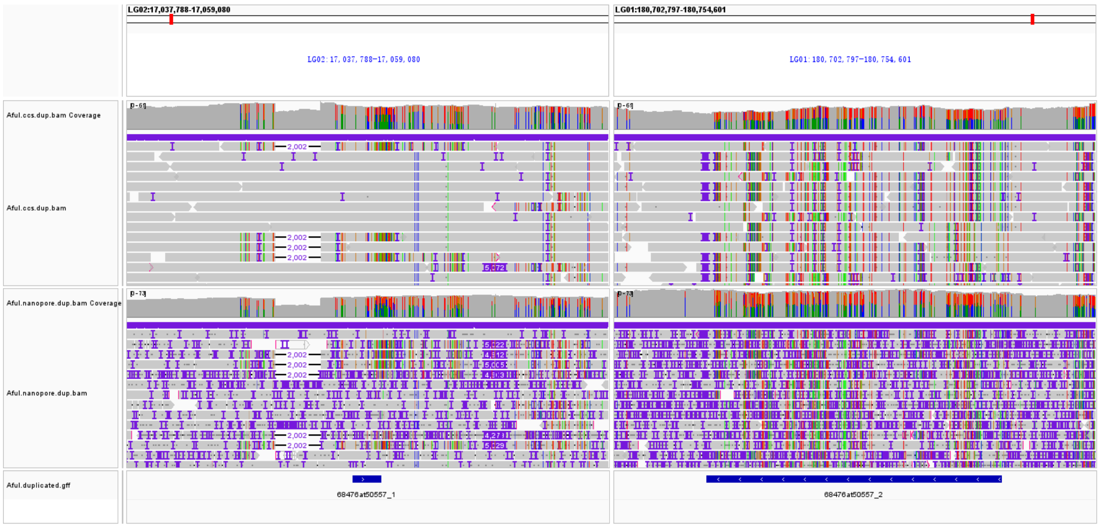

Supplementary Fig. 42 | IGV browser view of PacBio HiFi and ONT reads mapping on the duplicated BUSCO gene (geneid: 68476at50557) in *A. fulloi.*

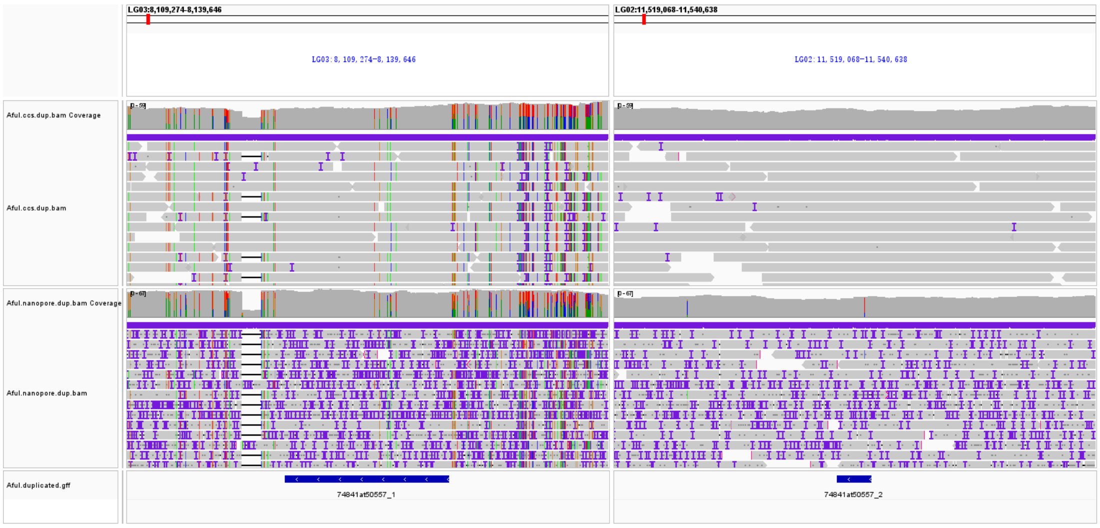

Supplementary Fig. 43 | IGV browser view of PacBio HiFi and ONT reads mapping on the duplicated BUSCO gene (geneid: 74841at50557) in *A. fulloi.*

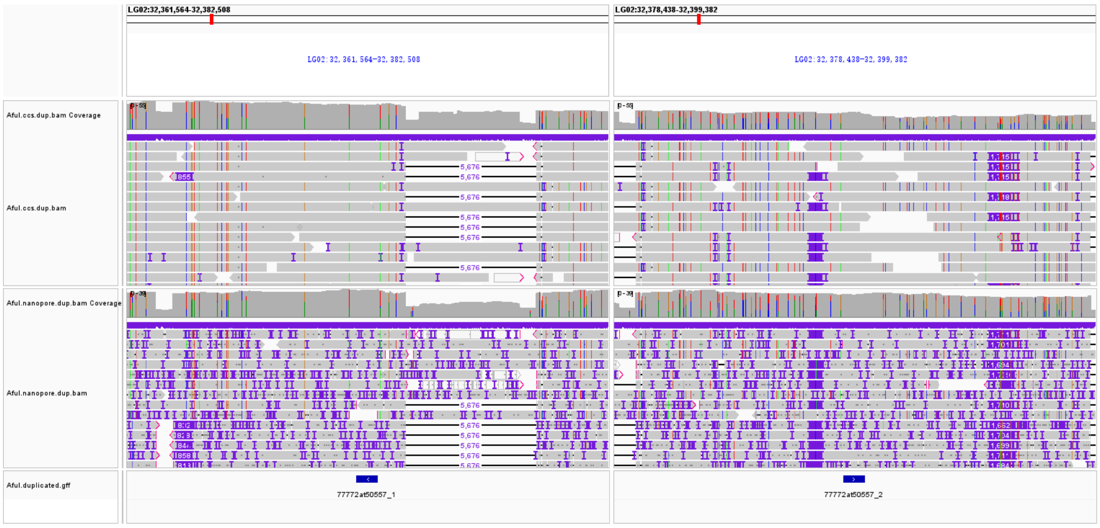

Supplementary Fig. 44 | IGV browser view of PacBio HiFi and ONT reads mapping on the duplicated BUSCO gene (geneid: 77772at50557) in *A. fulloi.*

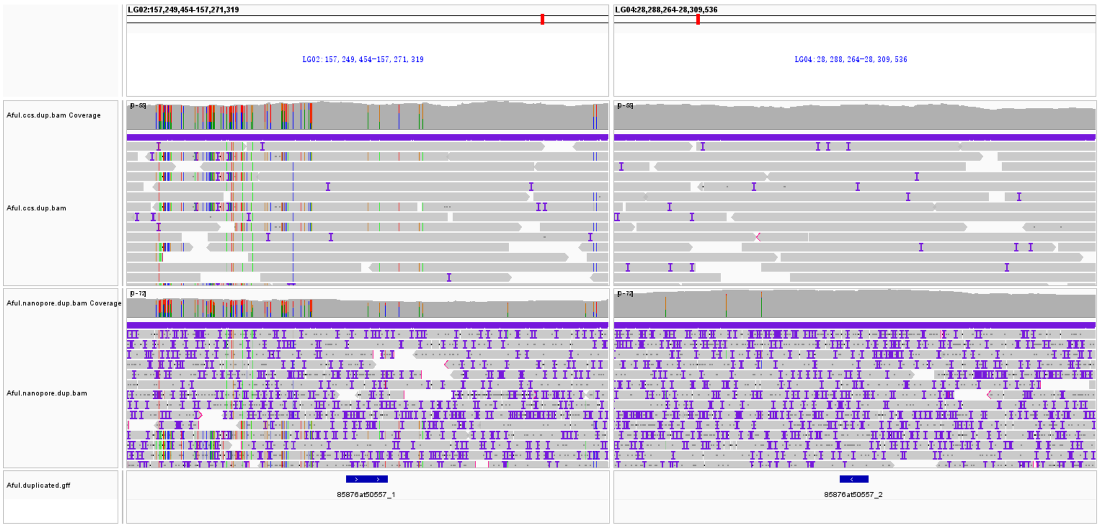

Supplementary Fig. 45 | IGV browser view of PacBio HiFi and ONT reads mapping on the duplicated BUSCO gene (geneid: 85876at50557) in *A. fulloi.*

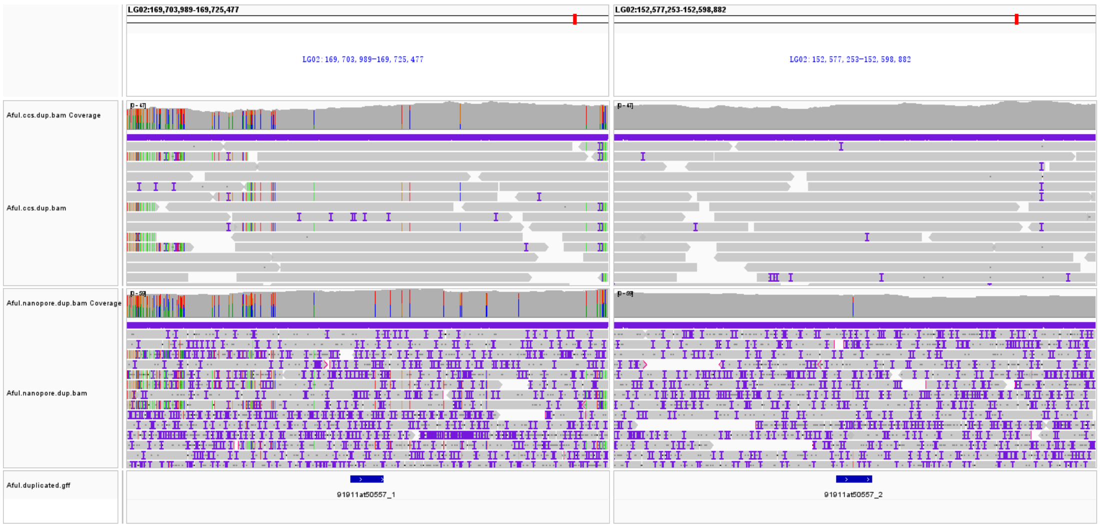

Supplementary Fig. 46 | IGV browser view of PacBio HiFi and ONT reads mapping on the duplicated BUSCO gene (geneid: 91911at50557) in *A. fulloi.*

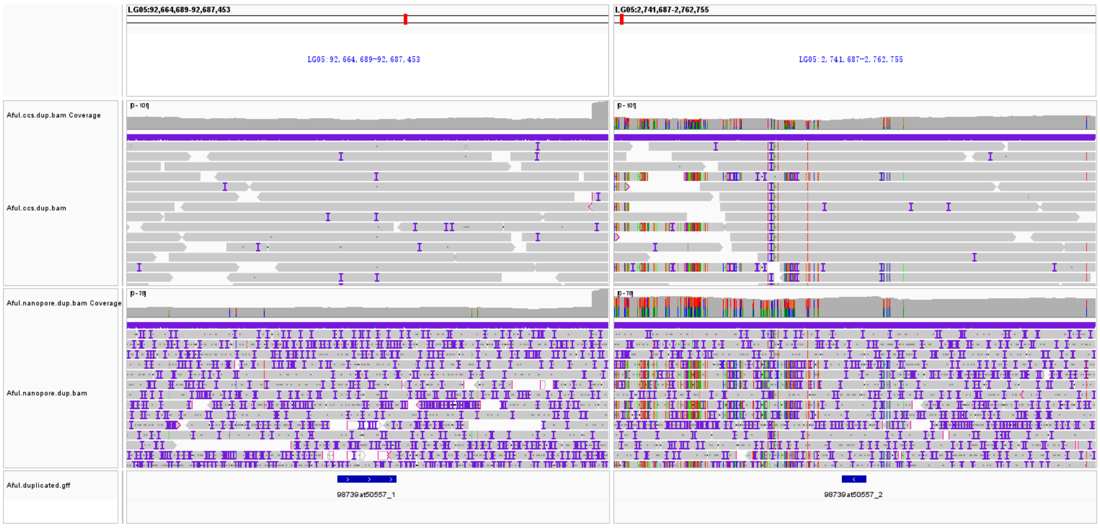

Supplementary Fig. 47 | IGV browser view of PacBio HiFi and ONT reads mapping on the duplicated BUSCO gene (geneid: 98739at50557) in *A. fulloi.*

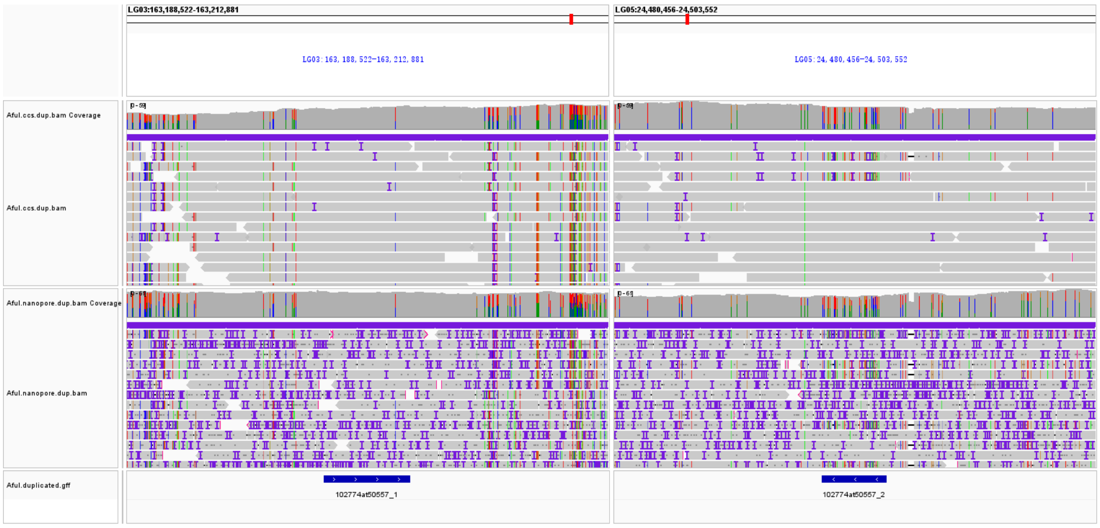

Supplementary Fig. 48 | IGV browser view of PacBio HiFi and ONT reads mapping on the duplicated BUSCO gene (geneid: 102774at50557) in *A. fulloi.*

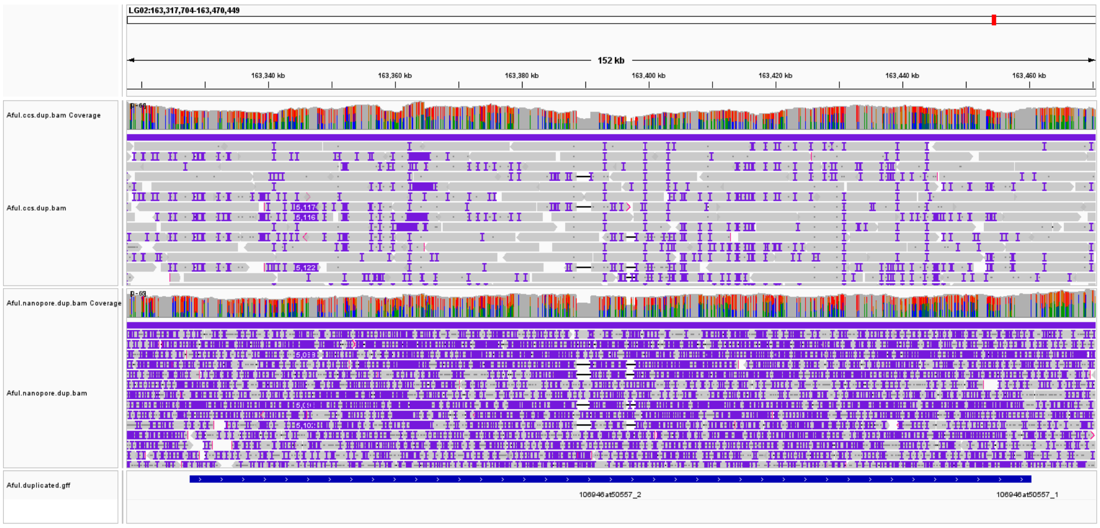

Supplementary Fig. 49 | IGV browser view of PacBio HiFi and ONT reads mapping on the duplicated BUSCO gene (geneid: 106946at50557) in *A. fulloi.*

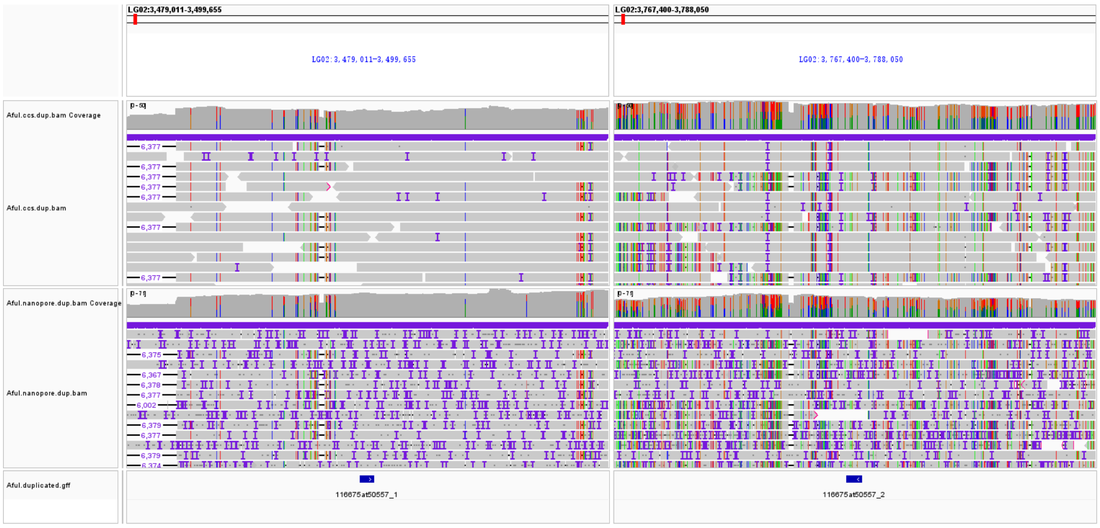

Supplementary Fig. 50 | IGV browser view of PacBio HiFi and ONT reads mapping on the duplicated BUSCO gene (geneid: 116675at50557) in *A. fulloi.*

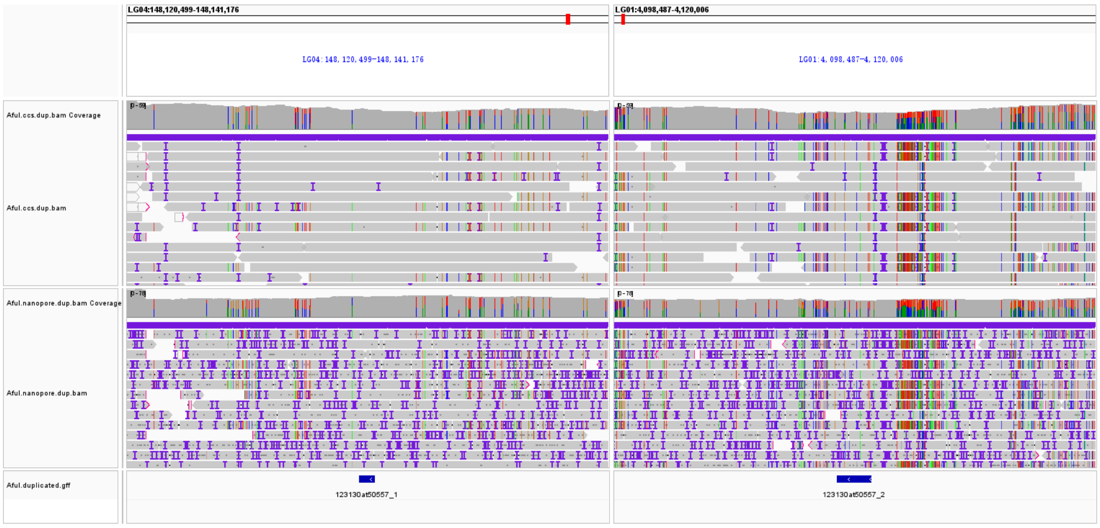

Supplementary Fig. 51 | IGV browser view of PacBio HiFi and ONT reads mapping on the duplicated BUSCO gene (geneid: 123130at50557) in *A. fulloi.*

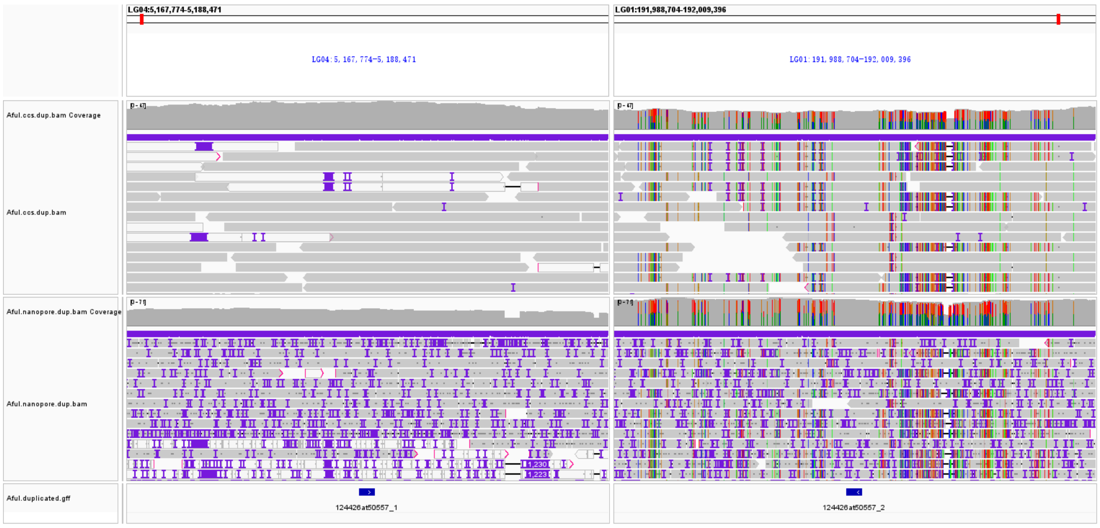

Supplementary Fig. 52 | IGV browser view of PacBio HiFi and ONT reads mapping on the duplicated BUSCO gene (geneid: 124426at50557) in *A. fulloi.*

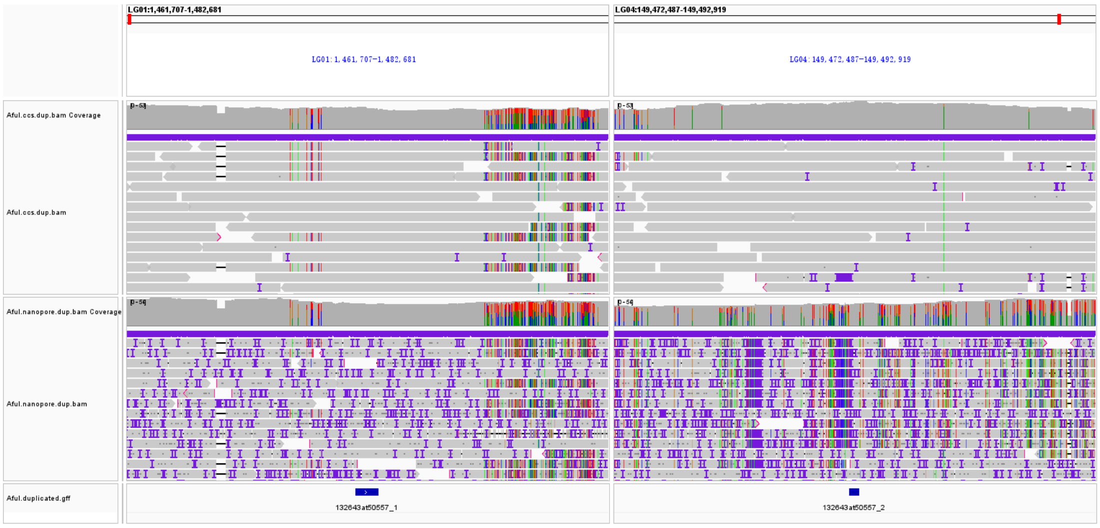

Supplementary Fig. 53 | IGV browser view of PacBio HiFi and ONT reads mapping on the duplicated BUSCO gene (geneid: 132643at50557) in *A. fulloi.*

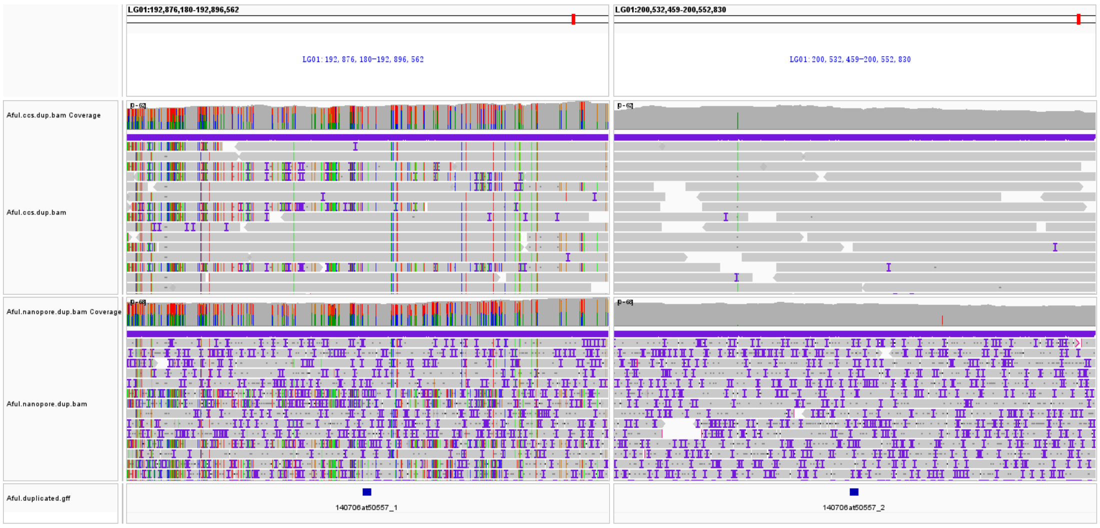

Supplementary Fig. 54 | IGV browser view of PacBio HiFi and ONT reads mapping on the duplicated BUSCO gene (geneid: 140706at50557) in *A. fulloi.*

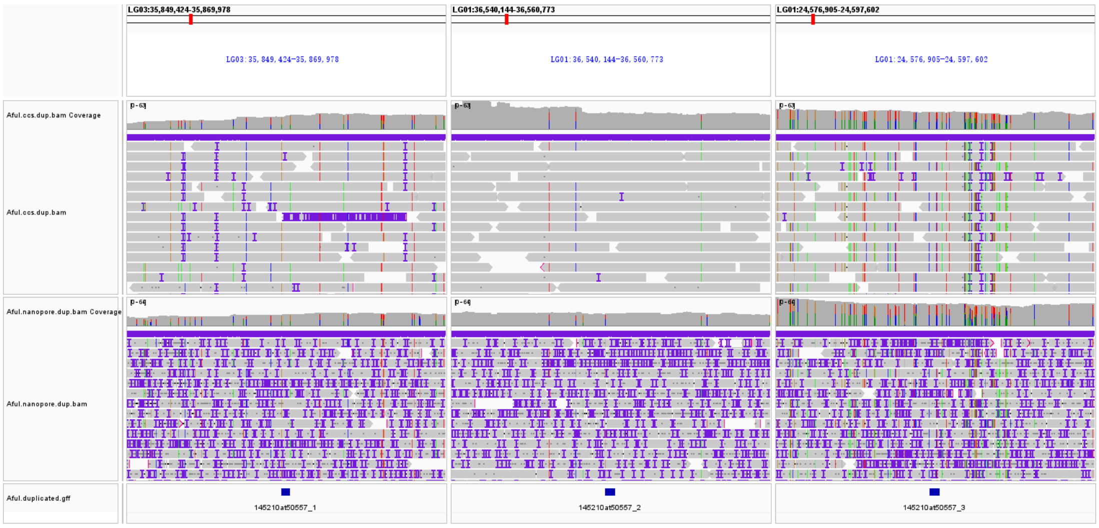

Supplementary Fig. 55 | IGV browser view of PacBio HiFi and ONT reads mapping on the duplicated BUSCO gene (geneid: 145210at50557) in *A. fulloi.*

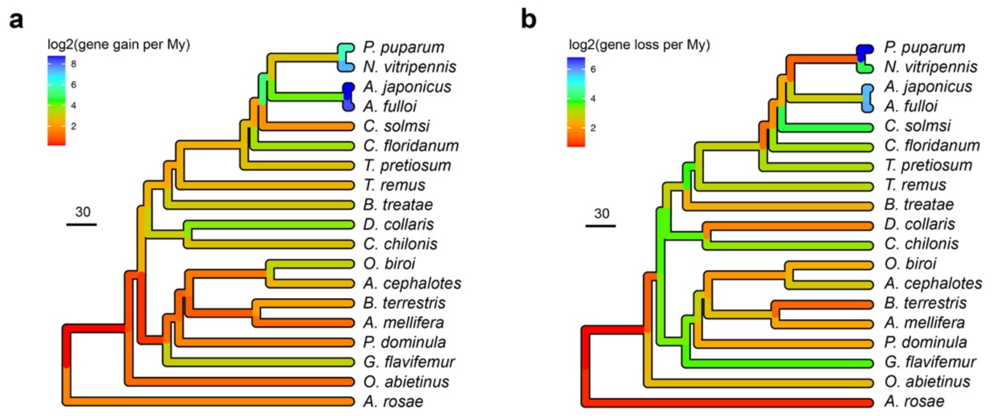

Supplementary Fig. 56 |. Rate of gene gain (a) and loss (b) along the hymenopteran phylogeny. All rates are color-indicated as branches of the phylogenetic tree. The phylogenetic tree was obtained from Fig. 1b. Source data are provided in Supplementary Data 5.

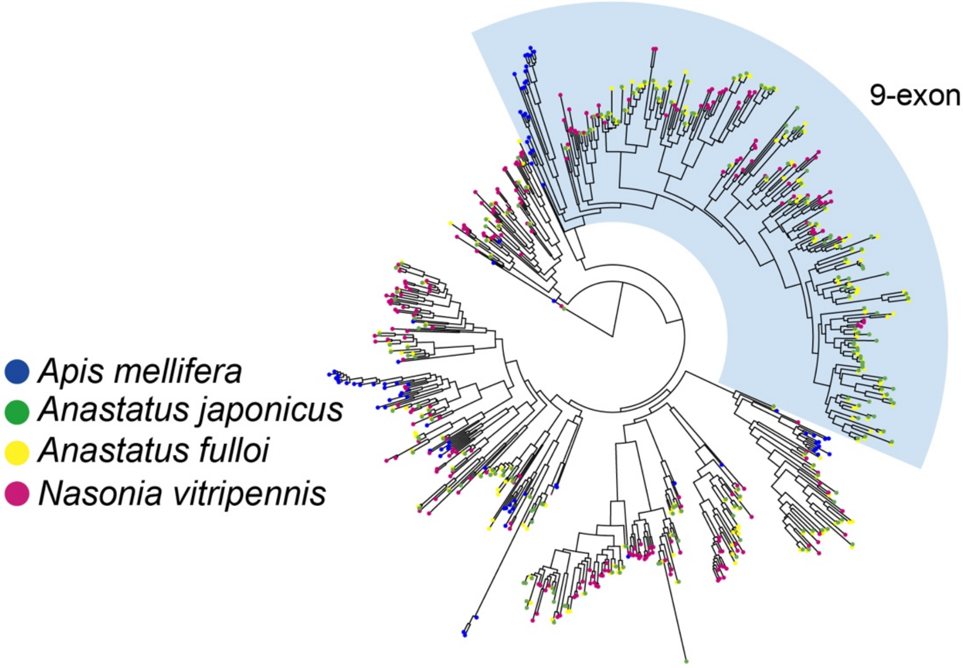

Supplementary Fig. 57 | Phylogenetic analysis of OR genes in four hymenopteran insects. This phylogeny showing the highly duplicated *Anastatus* OR genes in 9-exon subfamily comparing with *Nasonia vitripennis* and *Apis mellifera*. Source data are provided as a Source Data file.

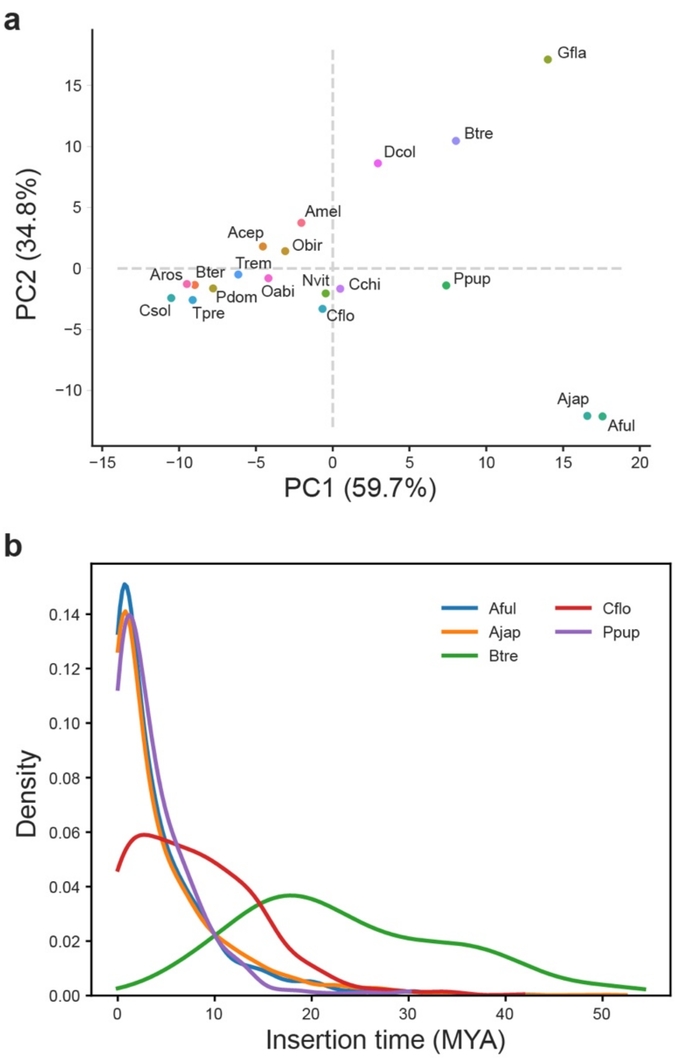

Supplementary Fig. 58 | TE features of the two *Anastatus* genomes. a, Principal component analysis of TEs (LTR retrotransposon, non-LTR retrotransposon, DNA transposon, and Unknown) of hymenopterans. This analysis showing the unique TE feature of the two *Anastatus* genomes. **b**, Density plot showing the distribution of the estimated insertion time of full-length LTR retrotransposons in five hymenopteran insects. Source data are provided as a Source Data file.

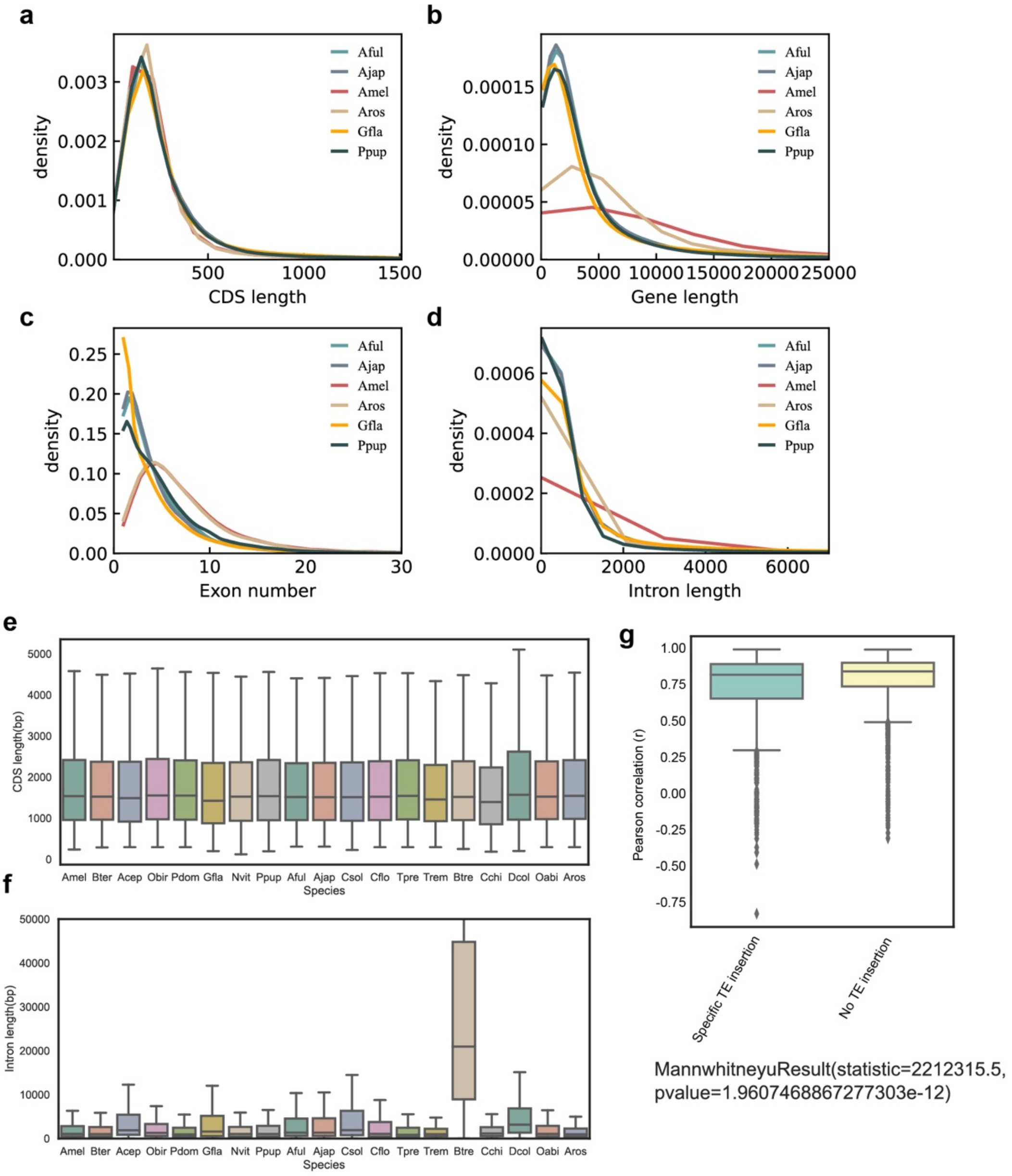

Supplementary Fig. 59 | Gene features of the two *Anastatus* genomes. Comparison of the length of CDS (**a**), gene (**b**), exon (**c**) and intron (**d**) among six selected hymenopteran species. Comparison of the length of CDS (**e**) and intron (**f**) using 1,792 strict one-to-one orthologous genes among 19 hymenopterans. **g**, Comparison of the expression correlation coefficient of the orthologous gene pairs in the two *Anastatus* wasps with specifical TE insertion in the potential regulatory region (1Kb upstream or downstream) and others without recent TE insertion. Two-sided Wilcoxon rank-sum test is used for statistical tests. $n_{\text{Specific TE insertion}}$ = 1571; $n_{\text{No TE insertion}}$ = 3219. Boxplots represent the median and interquartile range (IQR); whiskers mark 1.5x the IQR; data beyond 1.5x the IQR are plotted as individual points. Source data are provided as a Source Data file.

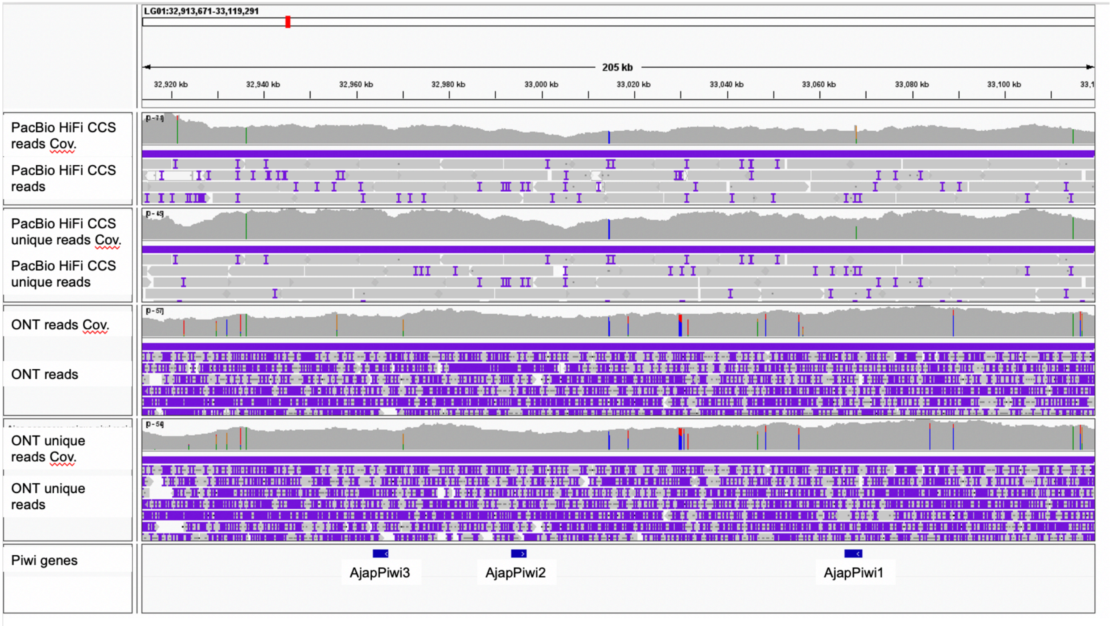

Supplementary Fig. 60 | PacBio HiFi and ONT reads spanning *Piwi* genes (*Piwi1*, *Piwi2*, *Piwi3*) in *A. japonicus*.

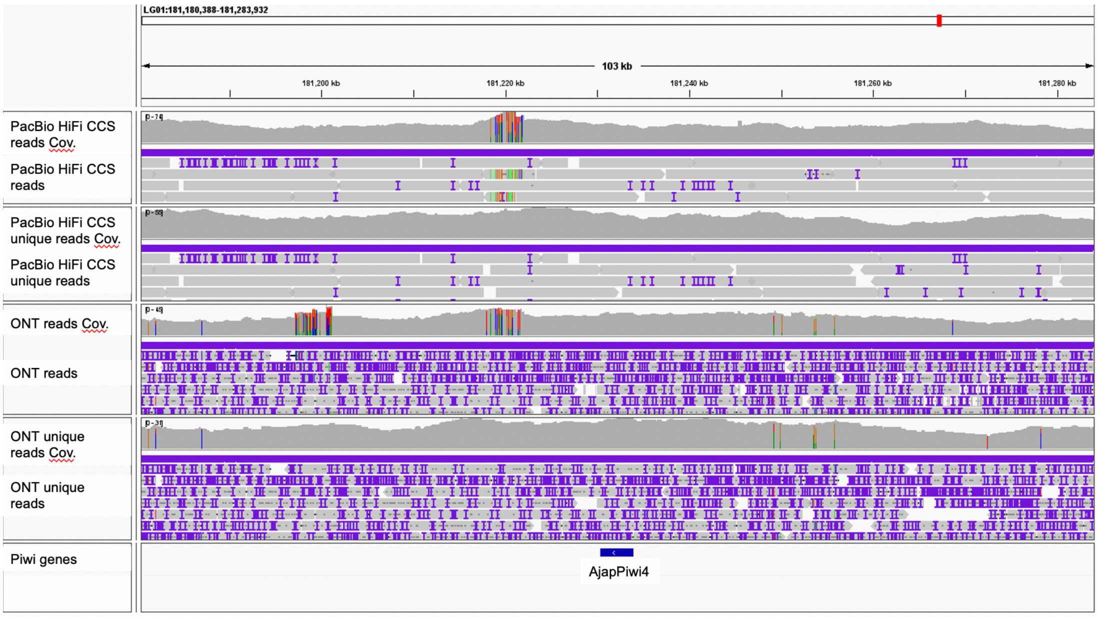

Supplementary Fig. 61 | PacBio HiFi and ONT reads spanning *Piwi* gene (*Piwi4*) in *A. japonicus*.

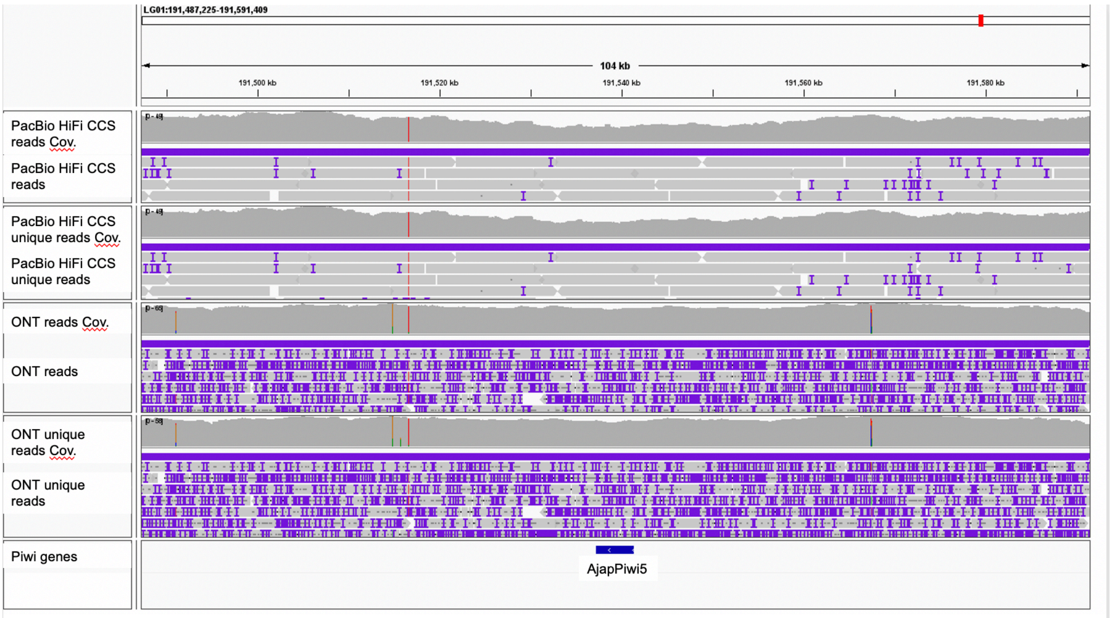

Supplementary Fig. 62 | PacBio HiFi and ONT reads spanning *Piwi* gene (*Piwi5*) in *A. japonicus*.

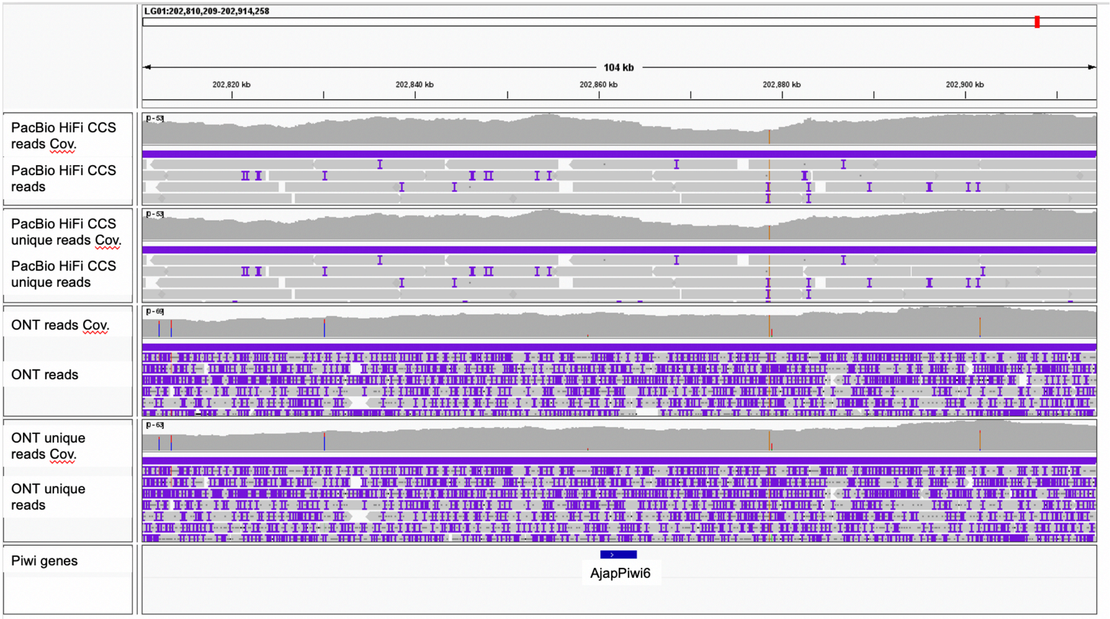

Supplementary Fig. 63 | PacBio HiFi and ONT reads spanning *Piwi* gene (*Piwi6*) in *A. japonicus*.

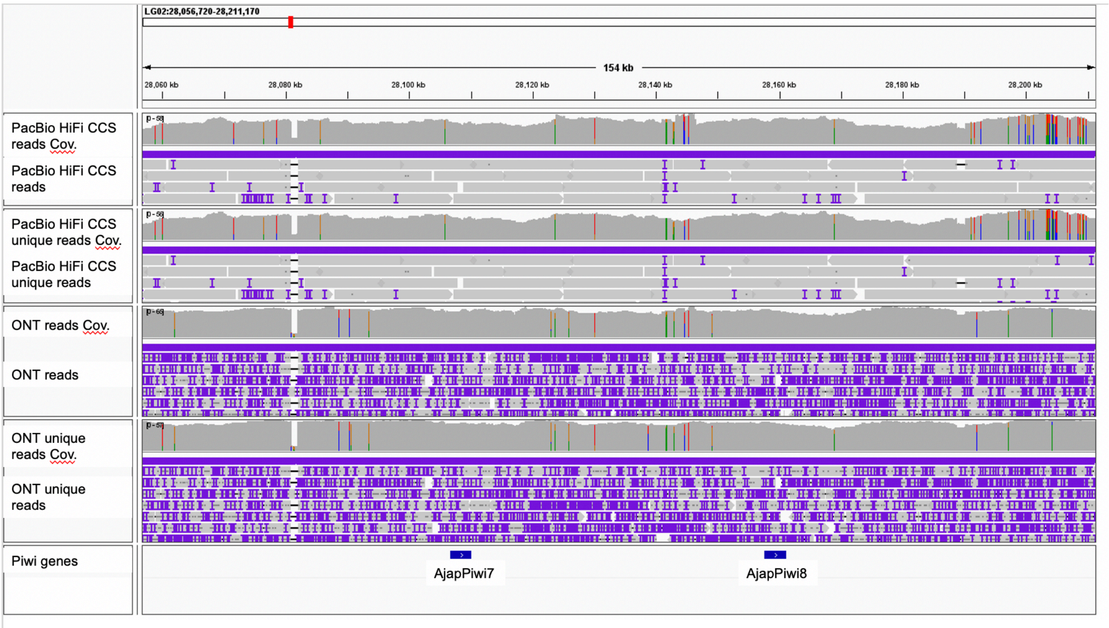

Supplementary Fig. 64 | PacBio HiFi and ONT reads spanning *Piwi* genes (*Piwi7-8*) in *A. japonicus*.

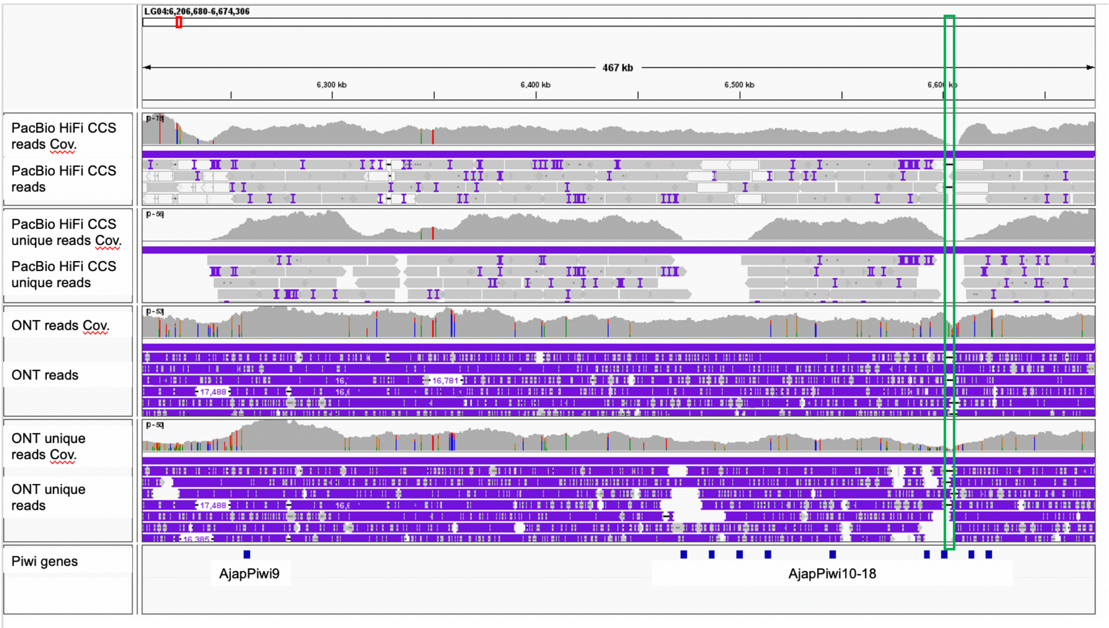

Supplementary Fig. 65 | PacBio HiFi and ONT reads spanning *Piwi* genes (*Piwi9-18*) in *A. japonicus*. The green box represents the gap (100Ns) concatenated contigs to create five chromosome-scale scaffolds.

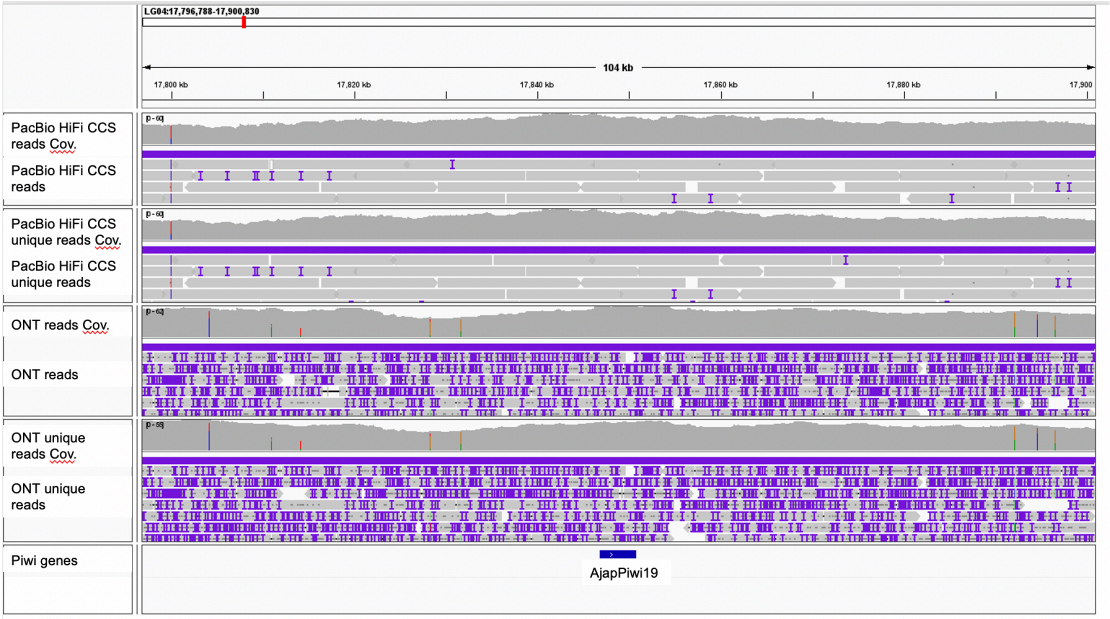

Supplementary Fig. 66 | PacBio HiFi and ONT reads spanning *Piwi* gene (*Piwi19*) in *A. japonicus*.

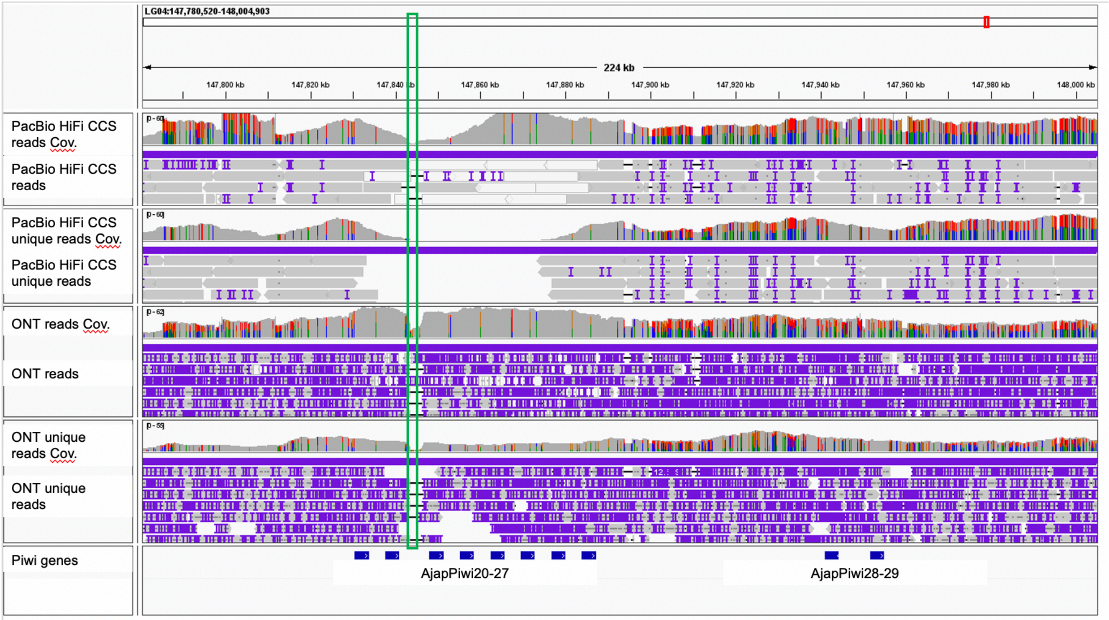

Supplementary Fig. 67 | PacBio HiFi and ONT reads spanning *Piwi* genes (*Piwi20-29*) in *A. japonicus*. The green box represents the gap (100Ns) concatenated contigs to create five chromosome-scale scaffolds.

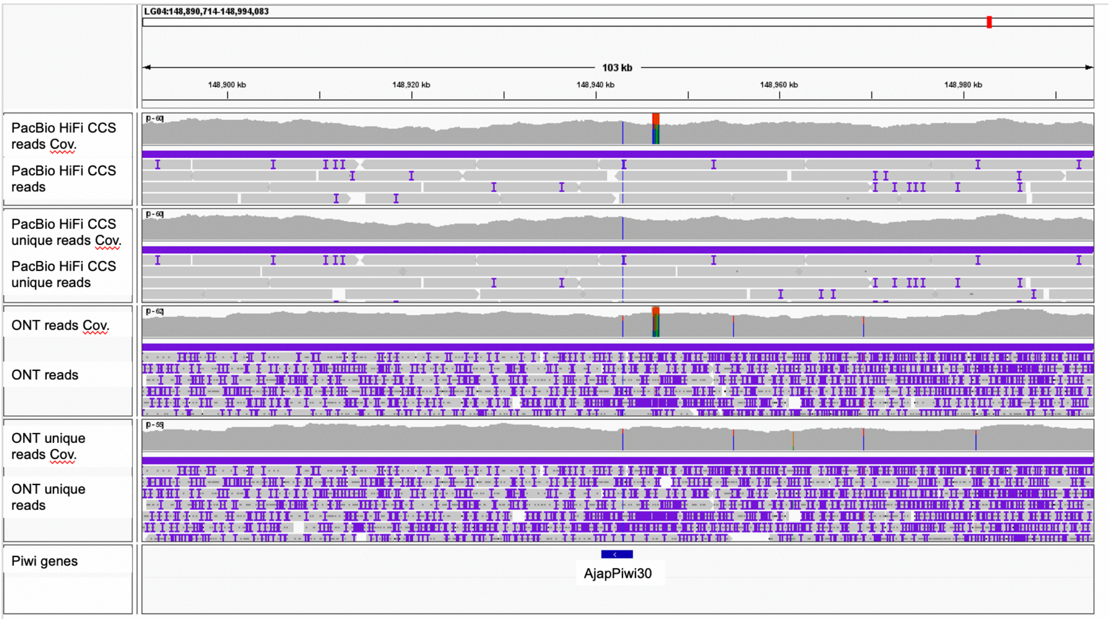

Supplementary Fig. 68 | PacBio HiFi and ONT reads spanning *Piwi* gene (*Piwi30*) in *A. japonicus*.

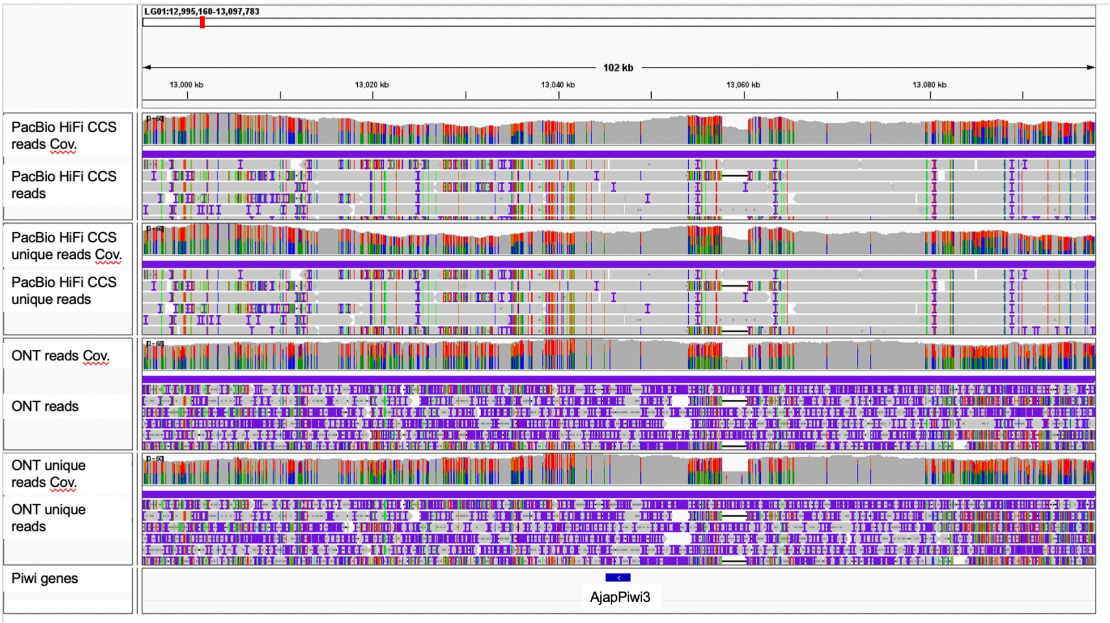

Supplementary Fig. 69 | PacBio HiFi and ONT reads spanning *Piwi* gene (*Piwi3*) in *A. fulloi*.

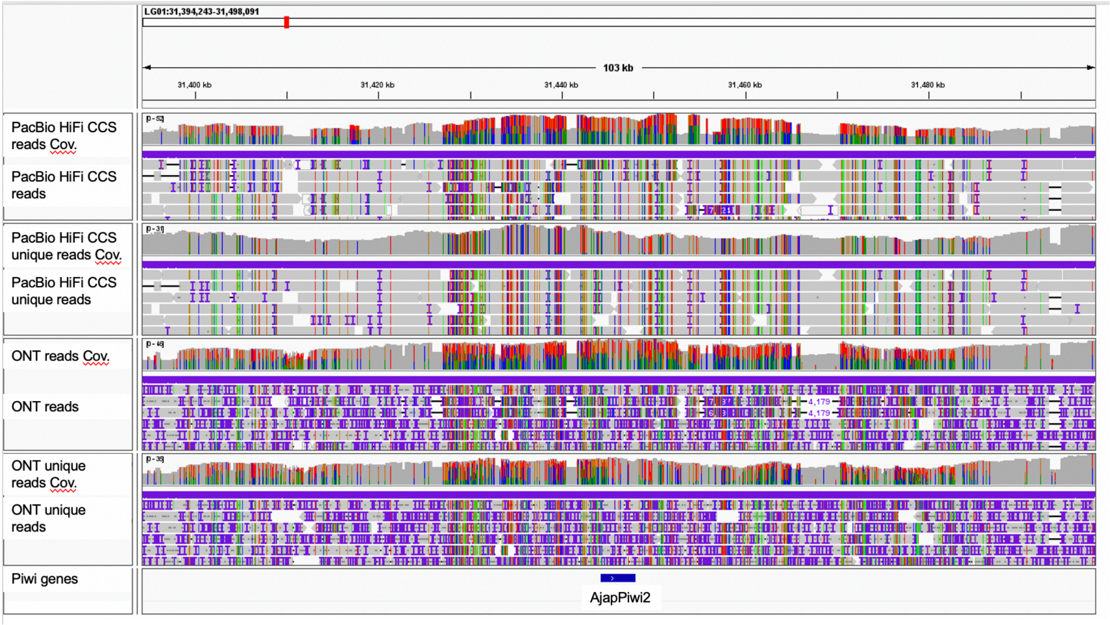

Supplementary Fig. 70 | PacBio HiFi and ONT reads spanning *Piwi* gene (*Piwi2*) in *A. fulloi*.

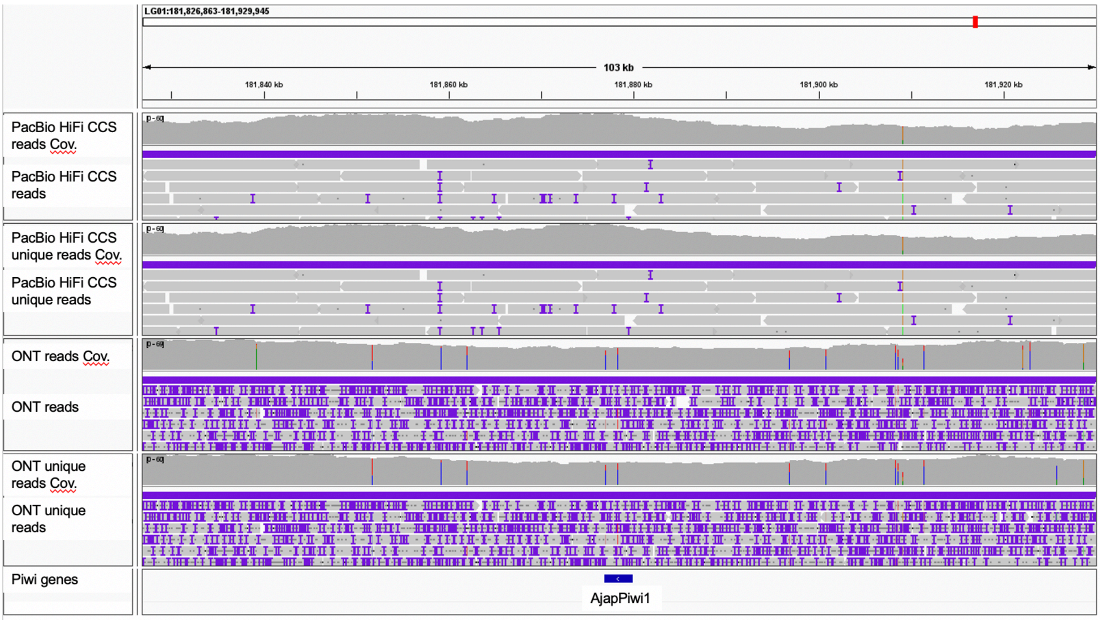

Supplementary Fig. 71 | PacBio HiFi and ONT reads spanning *Piwi* gene (*Piwi1*) in *A. fulloi*.

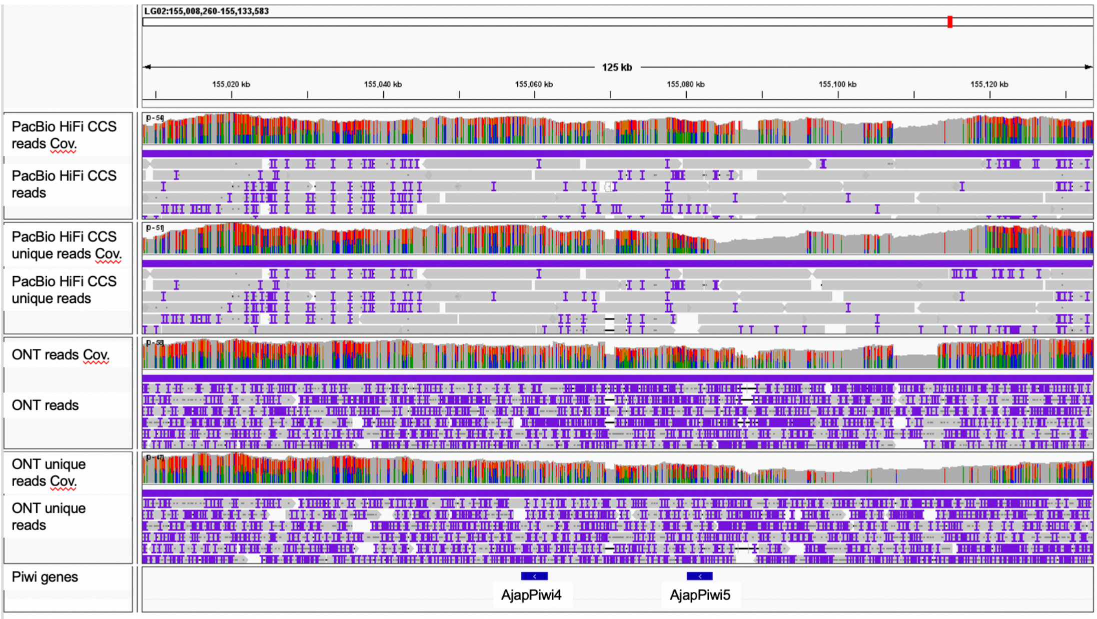

Supplementary Fig. 72 | PacBio HiFi and ONT reads spanning *Piwi* genes (*Piwi4-5*) in *A. fulloi*.

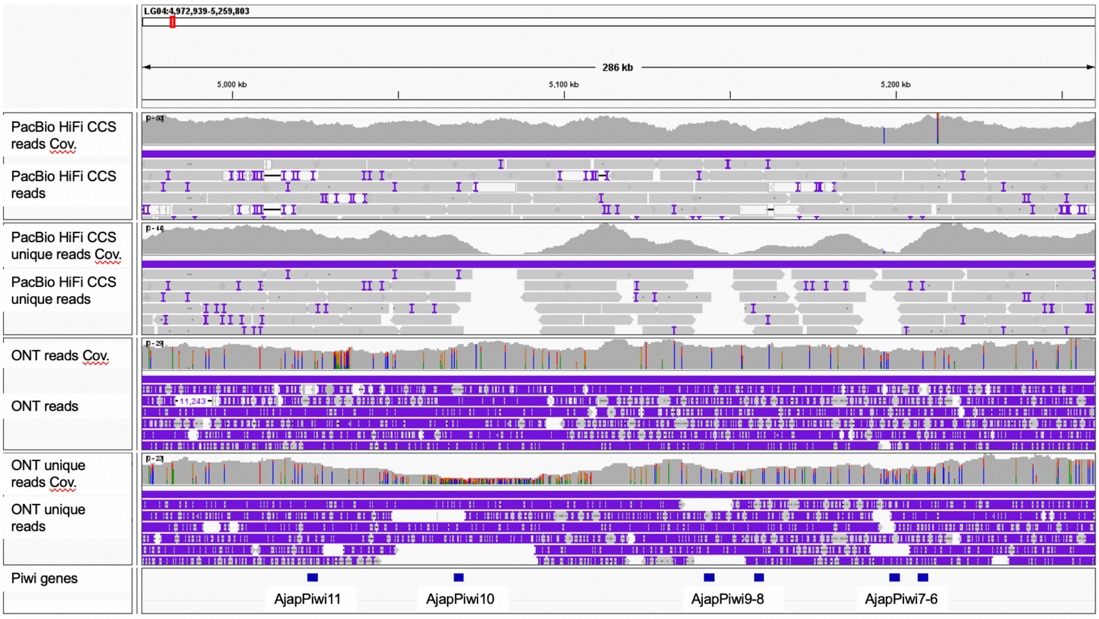

Supplementary Fig. 73 | PacBio HiFi and ONT reads spanning *Piwi* genes (*Piwi6-11*) in *A. fulloi*.

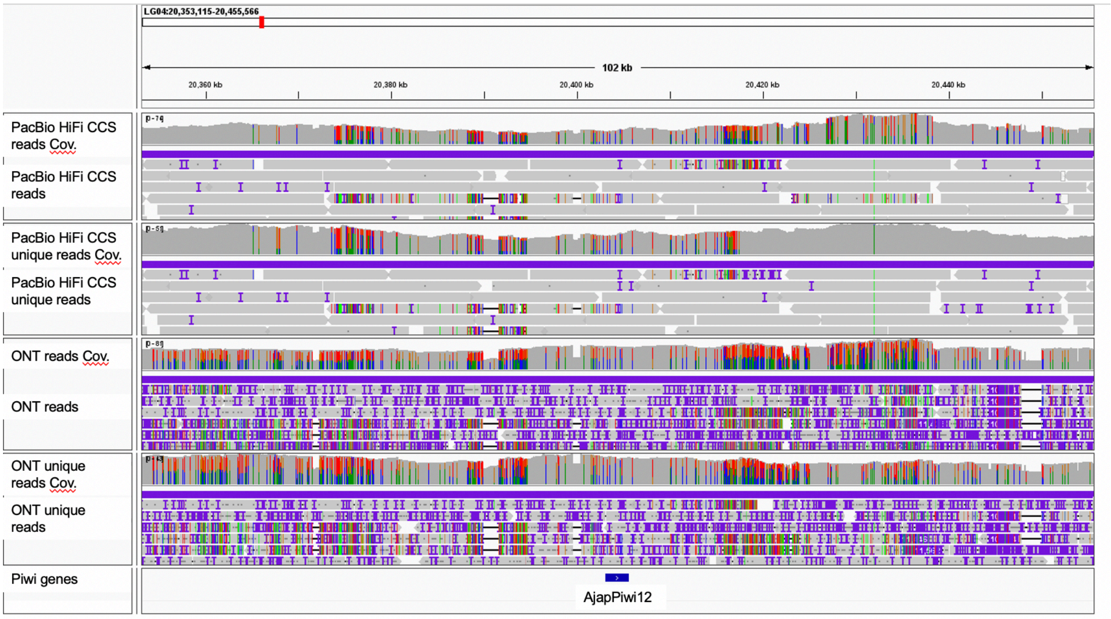

Supplementary Fig. 74 | PacBio HiFi and ONT reads spanning *Piwi* gene (*Piwi12*) in *A. fulloi*.

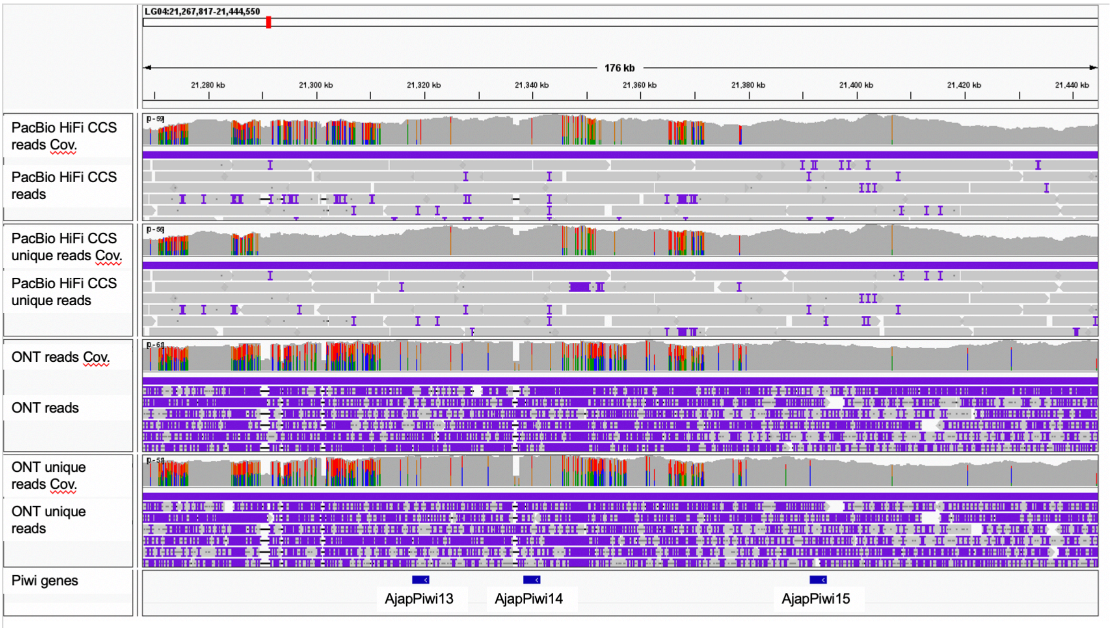

Supplementary Fig. 75 | PacBio HiFi and ONT reads spanning *Piwi* genes (*Piwi13-15*) in *A. fulloi*.

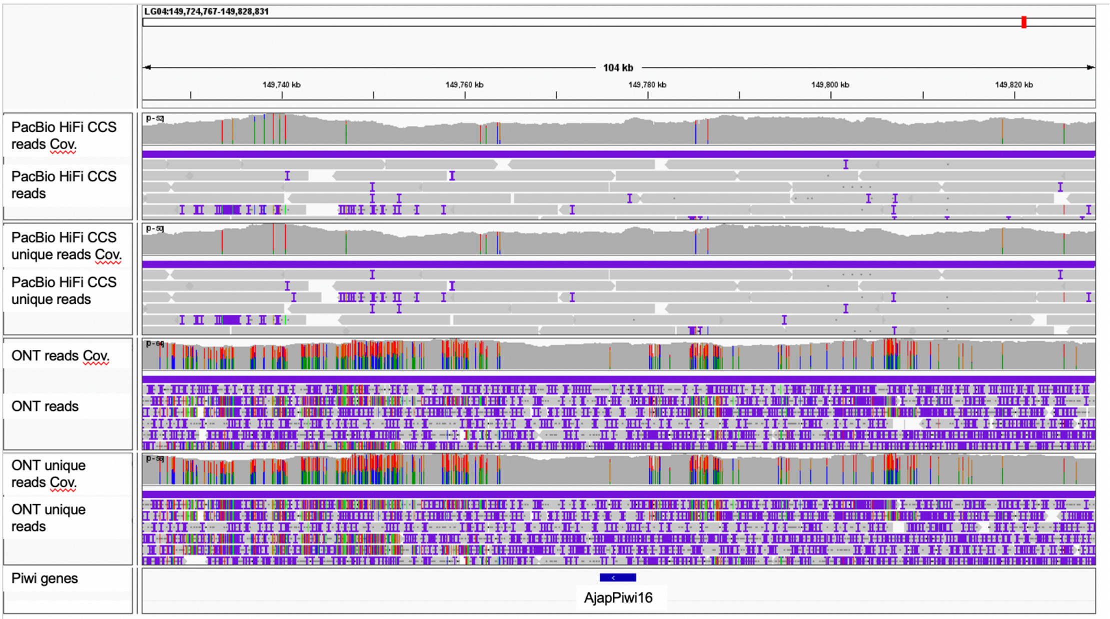

Supplementary Fig. 76 | PacBio HiFi and ONT reads spanning *Piwi* gene (*Piwi16*) in *A. fulloi*.

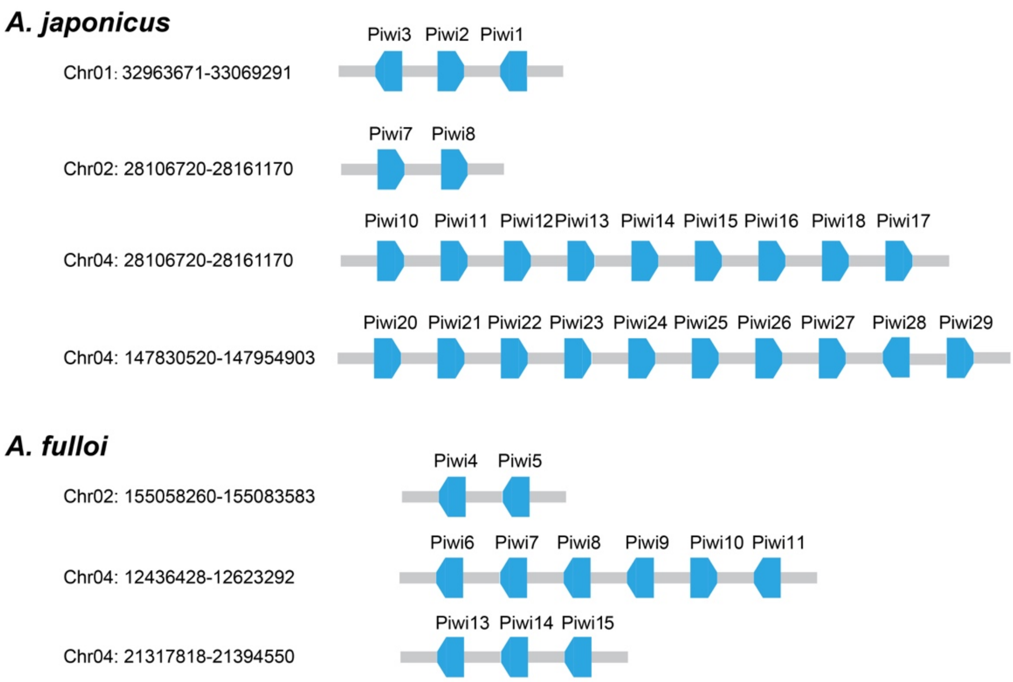

Supplementary Fig. 77 | Tandem repeats of *Piwi* genes in the two *Anastatus* genomes.

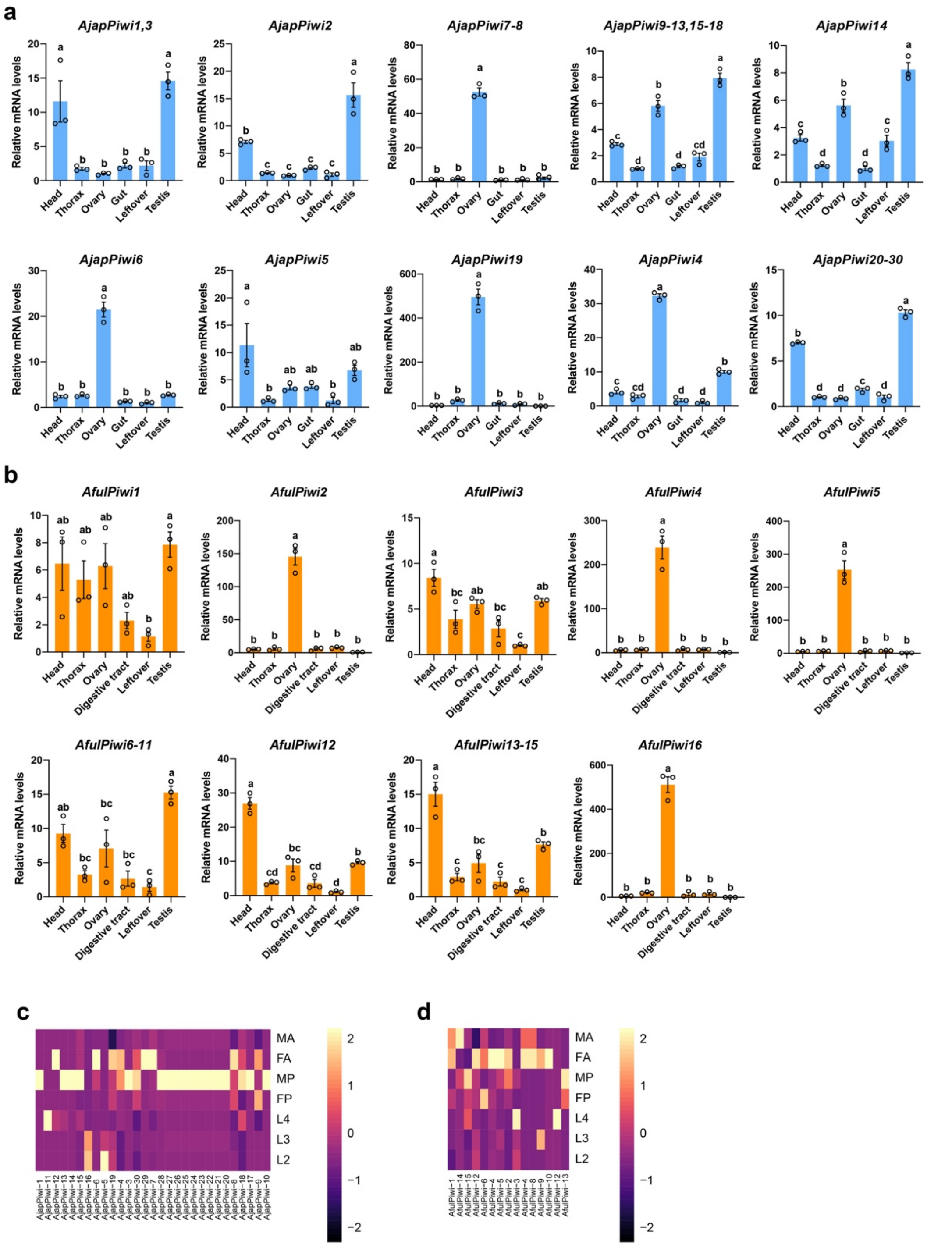

Supplementary Fig. 78 | Expression pattern of *Piwi* genes. a, qRT-PCR based gene expression of *Piwi* genes in the different tissues of adult *A. japonicus* wasp. **b**, qRT-PCR based gene expression of *Piwi* genes in the different tissues of adult *A. fulloi* wasp. The head, thorax, ovary, digestive tract and leftover from female adult wasps and testis from male adult wasps are used for qRT-PCR analyses. Data are presented as mean values +/- SEM from three biological replicates. Lowercase letters indicate significant differences (one-way ANOVA followed by Tukey's multiple comparison test, $p < 0.05$). Exact p values are presented in Supplementary Data 33. Due to the high sequence similarity of these duplicated genes, it is difficult to quantify the mRNA level of

each gene precisely. Thus, we designed primers to determine the expression of gene sets with high sequence similarity. **c**, RNA-seq based gene expression of *Piwi* genes among developmental stages of *A. japonicus*. **d**, RNA-seq based gene expression of *Piwi* genes among developmental stages of *A. fulloi*. The values of log2(TPM+1) are used for visualization. L2, 2nd instar larva; L3, 3rd instar larva; L4, 4th instar larva; MP, male pupa; FP, female pupa; MA, male adult; FA, female adult. Source data are provided as a Source Data file.

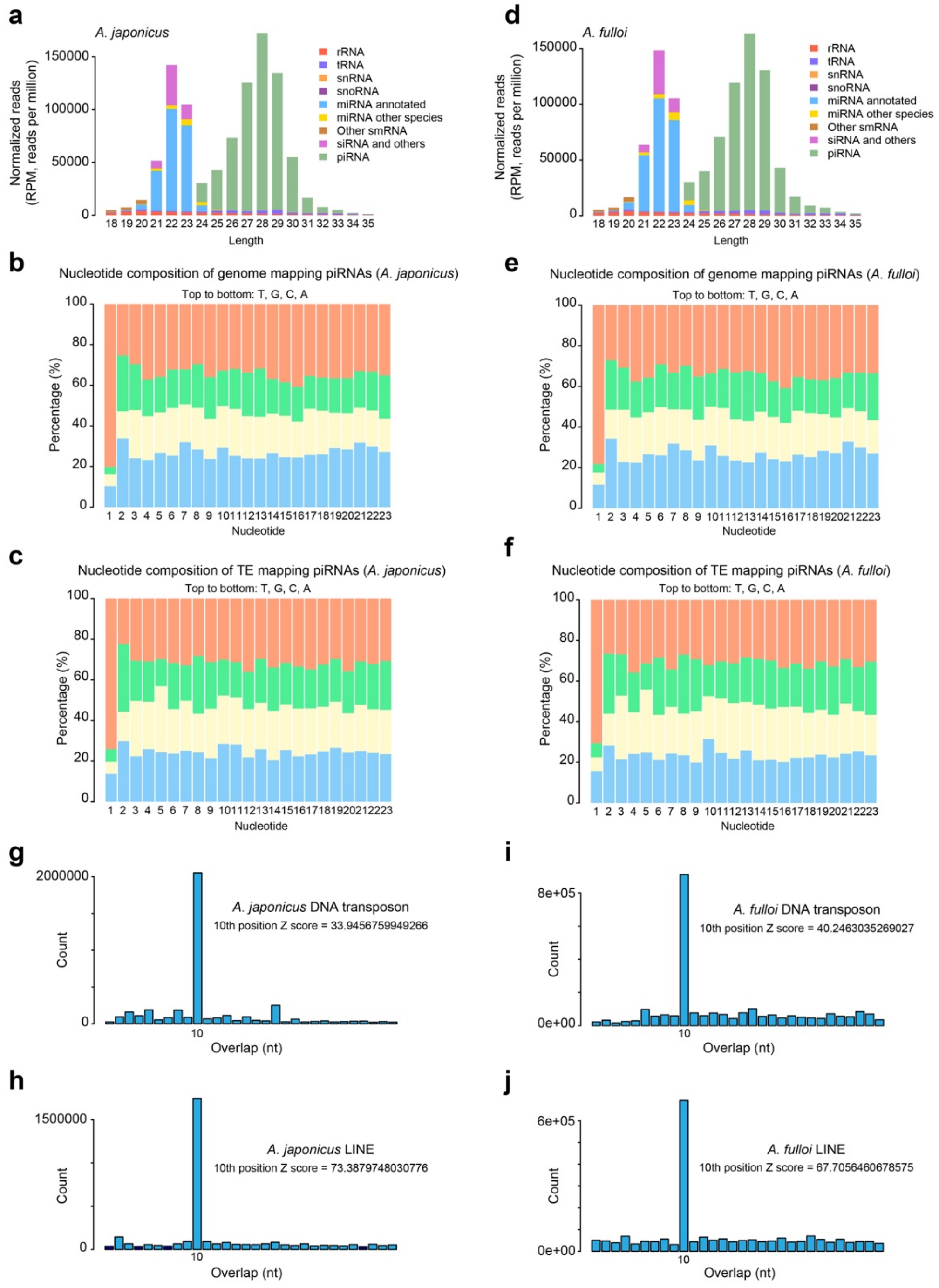

Supplementary Fig. 79 | Small RNA sequencing and piRNA analyses. Distribution of small RNA read sizes mapping to the *A. japonicus* genome (**a**) and the *A. fulloi* genome (**d**). The different kinds of small RNA were identified and shown in different colors. **b** and **e**, The nucleotide composition of genome mapping piRNAs of *A. japonicus* and *A. fulloi*. **c** and **f**, The nucleotide composition of TE mapping piRNAs of *A. japonicus* and *A. fulloi*. **g**, **h**, **i** and **j**, Analyses of the 5'-5' overlap between piRNAs from two types of TEs (DNA and LINE) in *A. japonicus* and *A. fulloi*. Significance of ten-nucleotide overlap ('Ping-Pong') was determined using Z-score. Source data are provided as a Source Data file.

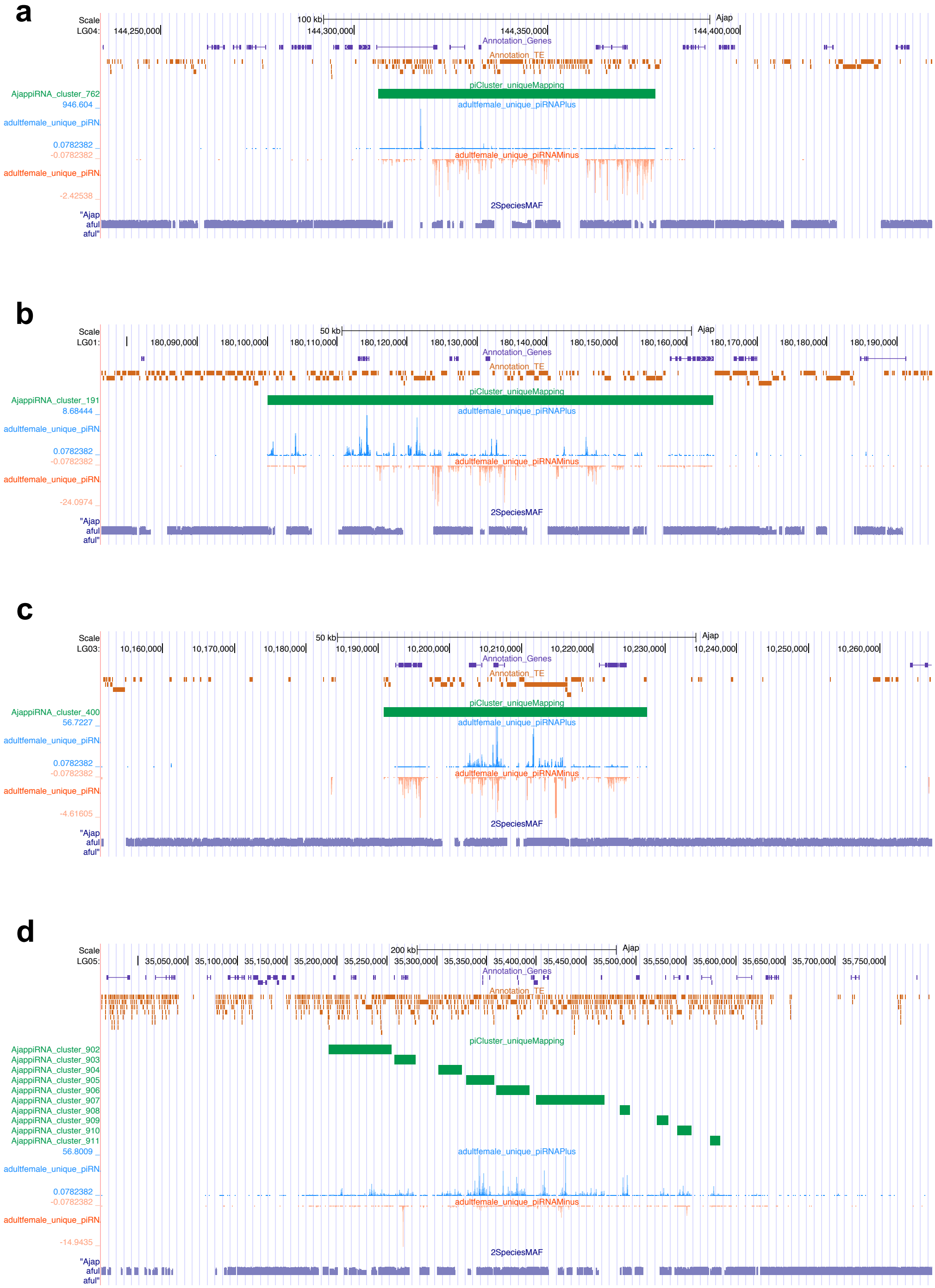

Supplementary Fig. 80 | Four examples of piRNA clusters in *A. japonicus*.

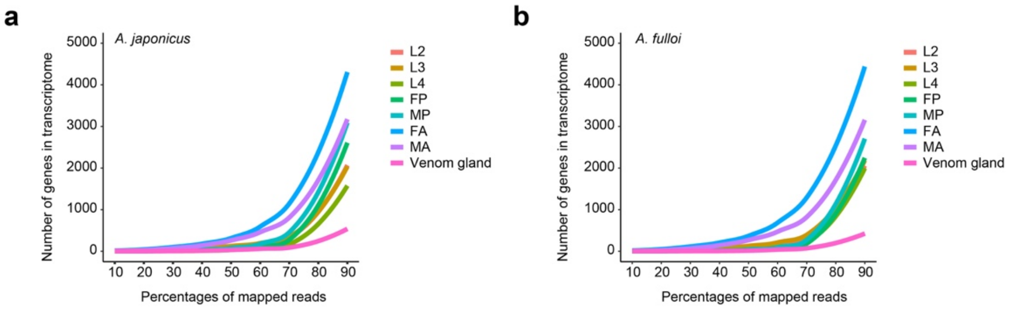

Supplementary Fig. 81 | The number of genes that account for different percentages of mapped reads in the venom gland transcriptome and other transcriptomes of developmental stages of *A. japonicus* (a) and *A. fulloi* (b). These figures showing the gene expression specialization in the venom gland. L2, 2nd instar larva; L3, 3rd instar larva; L4, 4th instar larva; MP, male pupa; FP, female pupa; MA, male adult; FA, female adult. Source data are provided as a Source Data file.

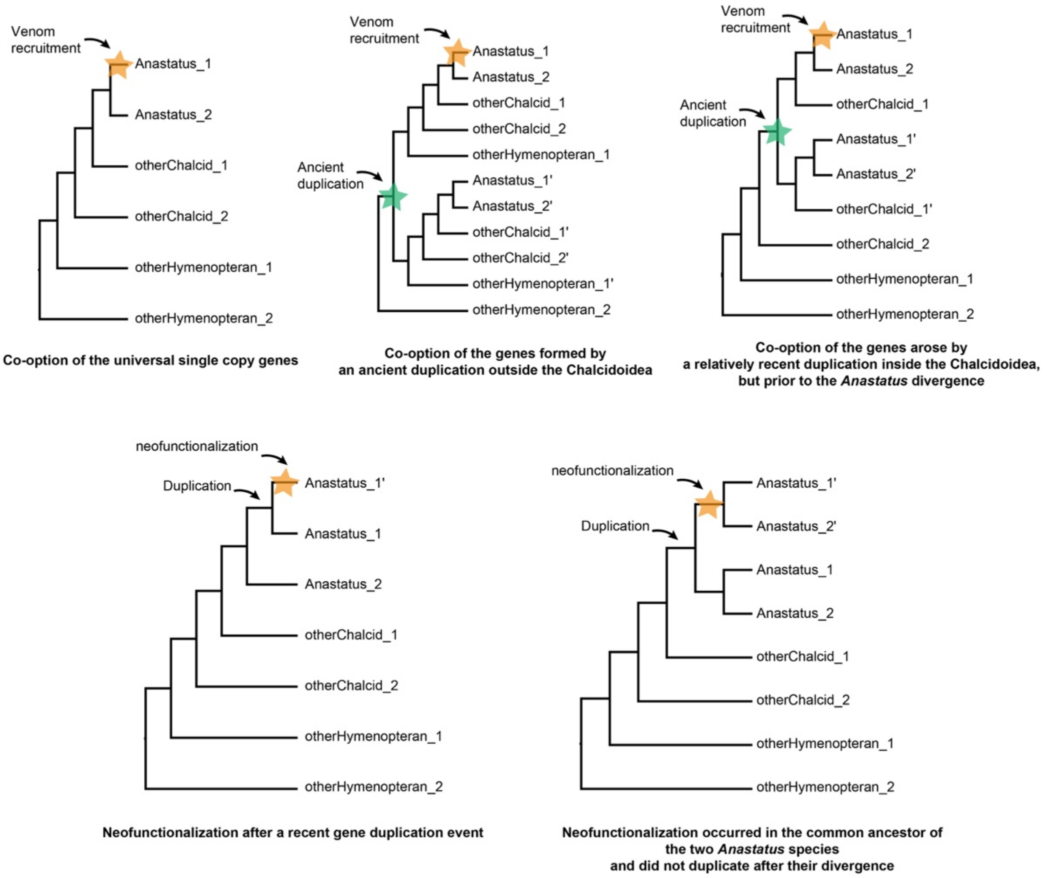

Supplementary Fig. 82 | The schematic diagrams of five evolution models. Three co-option venom recruitment models (Top): (1) co-option of the universal single copy genes, (2) co-option of the genes formed by an ancient duplication outside the Chalcidoidea, (3) co-option of the genes arose by a relatively recent duplication inside the Chalcidoidea, but prior to the *Anastatus* divergence. Two neofunctionalization models (Bottom): (1) the neofunctionalization followed by recent duplications, (2) the neofunctionalizations had occurred in the common ancestor of the two *Anastatus* species and did not duplicate after their divergence.

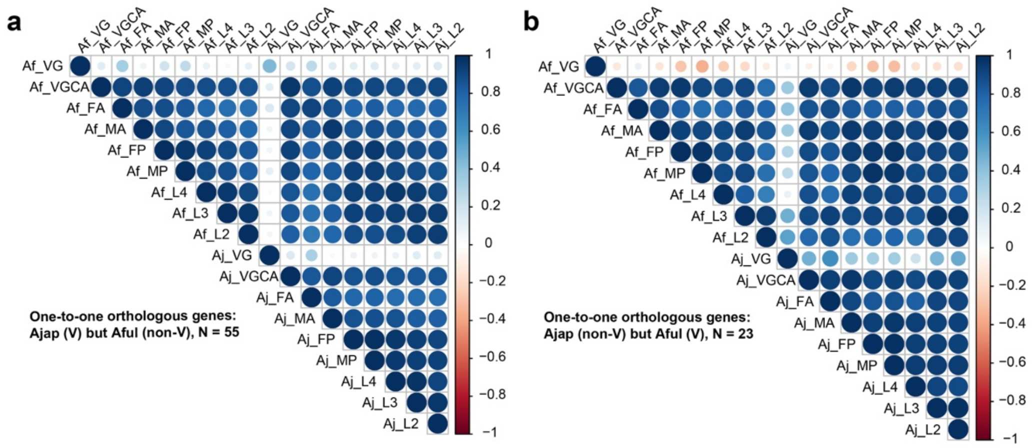

Supplementary Fig. 83 | Correlation analyses of the expression level between venom genes and their non-venom orthologs in the two *Anastatus* wasps. Source data are provided as a Source Data file.

Ajap025938.1, Chr02:17,692,034-17,696,336

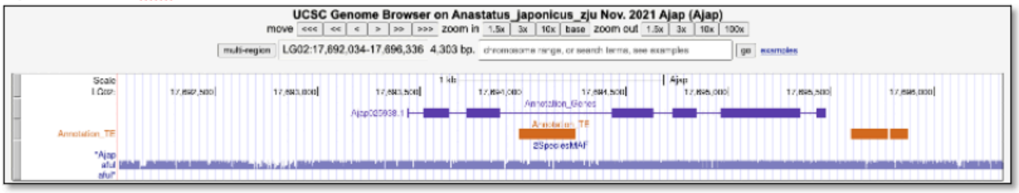

Ajap007659.1, Chr04:18,798,037-18,815,343

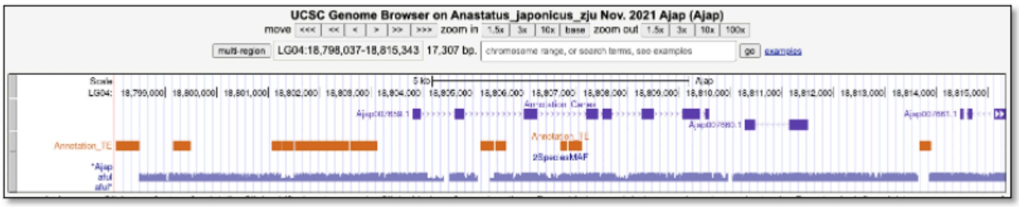

Ajap025973.1, Chr02:18,303,030-18,312,212

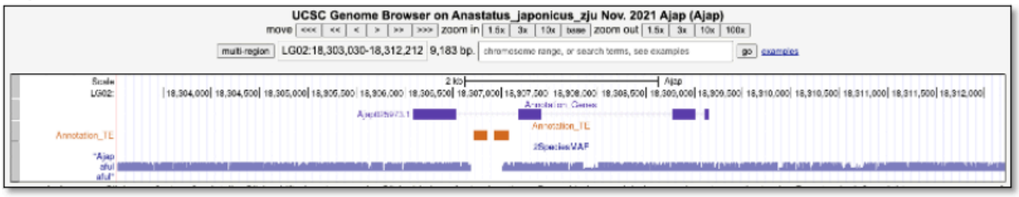

Ajap009930.1, Chr04:152,590,957-152,594,721

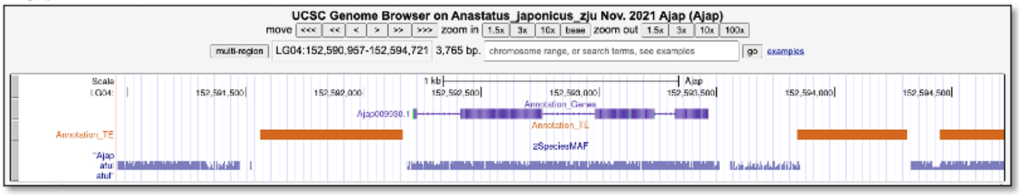

Ajap008554.1, Chr05:4,582,165-4,605,492

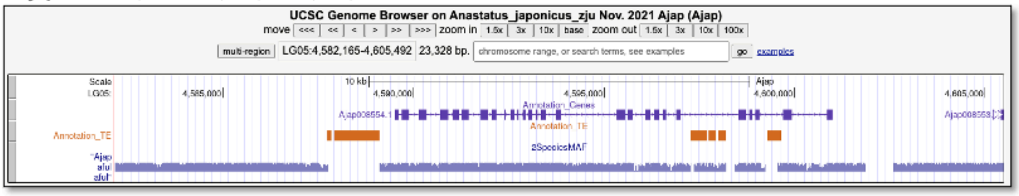

Ajap002190.1, Chr05:130,959,630-130,964,807

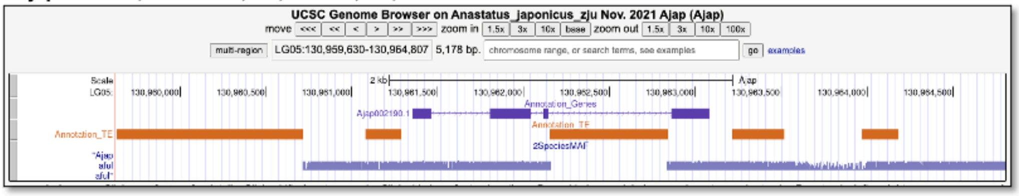

Supplementary Fig. 84 | Some representative examples of the NRER in the potential regulatory regions of venom genes, which may be relative to the large expression shift in venom gland between the two closely related species.

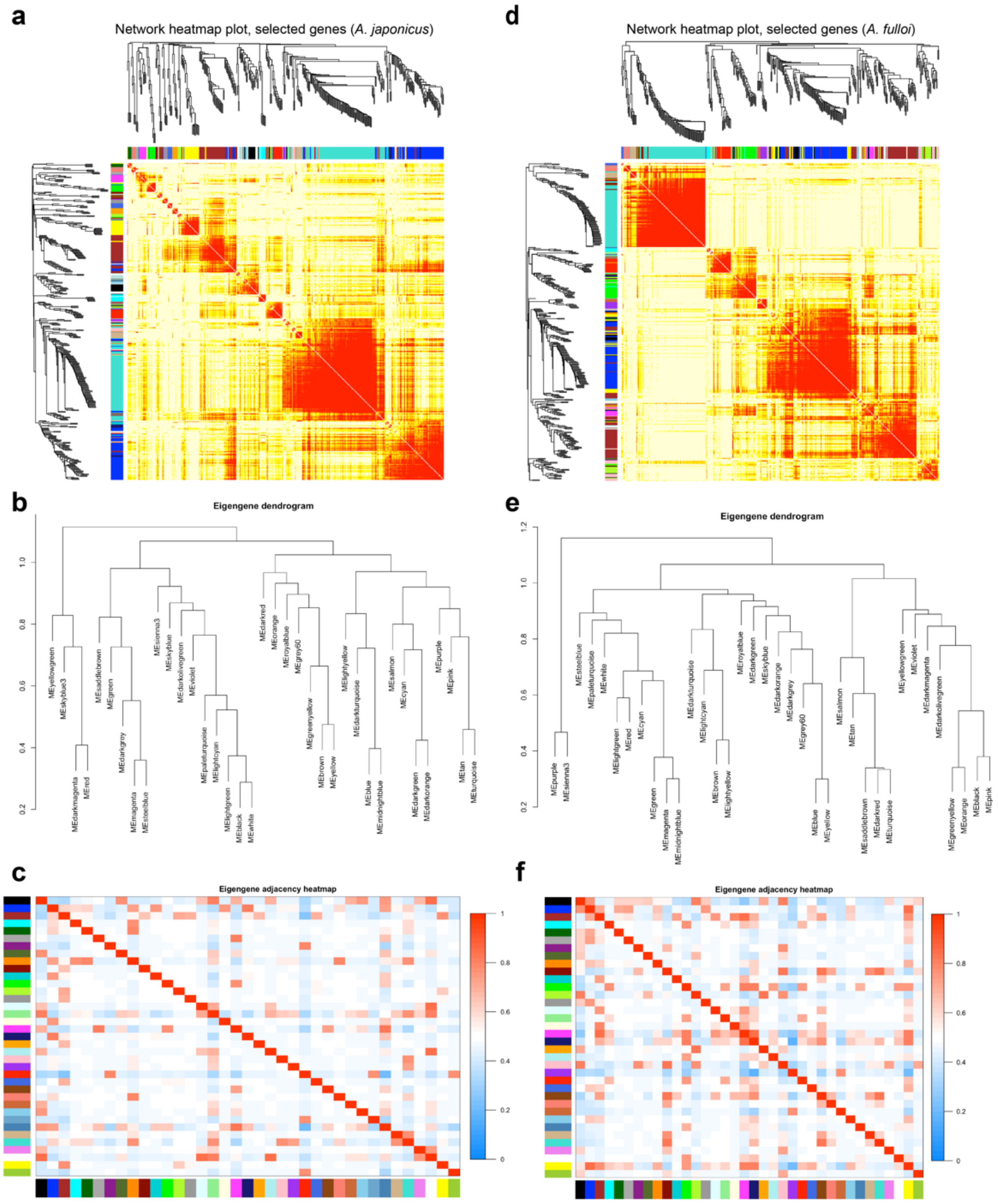

Supplementary Fig. 85 | WGCNA analyses of venom gland in two *Anastatus* wasps. a and **d**, Interaction plots of co-expression genes based on TOM (Topological Overlap Matrix) dissimilarity and the cluster dendrogram of 400 randomly selected genes in the two *Anastatus* wasps. **b** and **e**, The eigengene dendrograms identified groups of correlated modules in the two *Anastatus* wasps (left: *A. japonicus*, right: *A. fulloi*). **c** and **f**, The eigengene adjacency heatmaps of different gene co-expression modules in the two *Anastatus* wasps (left: *A. japonicus*, right: *A. fulloi*).

Supplementary Table 1. Statistics of Illumina sequencing.

Species	Total reads	Total bases	Clean reads	Clean bases	Q20 rate	Q30 rate	GC
Anastatus japonicas	140,277,238	21,041,585,700	138,867,048	19,358,709,752	96.57%	90.55%	30.55%
Anastatus fulloi	144,490,236	21,673,535,400	143,221,500	19,934,975,412	97.19%	91.94%	29.99%

Supplementary Table 2. statistics of Pacbio HiFi sequencing.

Species	Reads number	Pass reads mean length / bp	Pass reads max length / bp	Pass reads N50 length /bp	>10kb _ratio	>20kb _ratio
Anastatus japonicas	2,333,285	14,619	44,282	14,697	99.47	5.46
Anastatus fulloi	2,259,919	15,223	49,456	15,276	99.28	7.60

Supplementary Table 3. Statistics of Hi-C sequencing.

	Anastatus japonicas	*Anastatus fulloi*
Raw Paired-end Reads	982,354,384	1,216,631,552
Clean Paired-end Reads	982,326,288	1,216,604,938
Clean Bases(bp)	147,061,058,974	181,777,693,134
Clean Paired-end Reads Rate (%)	100.00	100.00
Clean Q30 Bases Rate (%)	91.45	91.66

Supplementary Table 4. Length of chromosomes.

Chr ID	*Anastatus japonicas*	*Anastatus fulloi*
Chr01	216,296,257	208,267,002
Chr02	181,361,897	182,570,706
Chr03	178,301,815	177,026,914
Chr04	167,252,468	161,898,282
Chr05	157,193,773	160,229,835
Total	900,406,210	889,992,739

Supplementary Table 5. Assembly statistics of the genome of two *Anastatus* wasps, *A. japonicus* and *A. fulloi*.

	Anastatus japonicas	*Anastatus fulloi*
Genome size (bp)	950,927,185	963,363,924
Number of contigs	614	375
Number of scaffolds	230	211
Scaffold N50	178,308,215	160,233,335
Number of chromosomes	5	5
Number of protein-coding genes	27,792	27,168

Supplementary Table 6. k-mer analyses of the genome of two *Anastatus* wasps, *A. japonicus* and *A. fulloi*.

	Anastatus japonicas	*Anastatus fulloi*
K-mer	17	17
K-mer depth	18	18
K-mer number	17,126,012,745	17,608,318,811
Estimated genome size	951,445,152	978,239,933

Supplementary Table 7. BUSCO assessment of the genome of two *Anastatus* wasps, *A. japonicus* and *A. fulloi*.

	Anastatus japonicas	*Anastatus fulloi*
**Test with assembly**		
Complete	1,329 (97.3%)	1,326 (97.0%)
Complete and single-copy	1,294 (94.7%)	1,287 (94.1%)
Complete and duplicated	35 (2.6%)	39 (2.9%)
Fragmented	23 (1.7%)	24 (1.8%)
Missing	15 (1.0%)	17 (1.2%)
**Test with official gene set (OGS)**		
Complete	1,300 (95.1%)	1,295 (94.8%)
Complete and single-copy	1,243 (90.9%)	1,223 (89.5%)
Complete and duplicated	57 (4.2%)	72 (5.3%)
Fragmented	24 (1.8%)	23 (1.7%)
Missing	43 (3.1%)	49 (3.5%)

Supplementary Table 8. The mapping rates with Illumina and PacBio sequencing reads of the two *Anastatus* genomes.

	Anastatus japonicas		*Anastatus fulloi*	
Library	Ajap Illumina genomic	Ajap PacBio genomic	Aful Illumina genomic	Aful PacBio genomic
Total Reads	140,750,492	2,405,076	294,811,014	2,522,557
Mapped Reads	140,562,849	2,405,035	294,276,959	2,519,393
Mapping Rates (%)	99.87	100.00	99.82	99.87
Paired Reads	140,277,238	-	292,967,928	-
Paired Mapping Reads	139,962,928	-	292,020,164	-
Properly Paired Reads	138,096,884	-	283,800,604	-
Properly Mapping Rates (%)	98.45	-	96.87	-

Supplementary Table 9. Statistics of Nanopore ultra-long read sequencing.

Species	Length of passed reads / bp	Mean length of passed reads / bp	Max length of passed reads / bp	reads N50 /bp	>20kb _ratio	>50kb _ratio
Anastatus japonicas	30,453,933,839	25,185	526,770	46,342	43%	14%
Anastatus fulloi	35,611,501,487	26,562	586,088	47,313	45%	15%

Supplementary Table 10. The mapping rate with PacBio ONT reads of the two *Anastatus* genomes.

Species	Mapped Reads	Mapped Reads (primary)	Mapping Rates	Mapping Rates (primary)
Anastatus japonicas	1,903,670	1,199,579	99.50%	99.21%
Anastatus fulloi	2,041,099	1,329,973	99.48%	99.20%

Supplementary Table 11. Mapped sequencing read coverage of the two *Anastatus* genomes.

	mean	sd
Ajap.HiFi	36.04774534	5.099207726
Ajap.ONT	30.85783526	8.485750056
Aful.HiFi	35.81994833	5.96924161
Aful.ONT	35.18961698	10.27573953

Supplementary Table 12. Odorant receptor subfamily size in the four hymenopteran species.

	A. japonicus	*A. fulloi*	*N. vitripennis*	*A. mellifera*
Orco	1	1	1	1
9-exon	104	102	72	22
A	1	1	2	3
D	3	4	10	0
E	10	14	29	1
F	17	12	23	1
H	15	17	12	12
I	5	4	1	1
J	0	0	0	8
L	5	5	9	22
M	1	0	2	1
N	1	1	1	0
Q	0	0	1	1
S	4	5	4	0
T	8	6	24	2
U	6	6	5	1
V	2	2	9	5

Z	8	12	18	1
ZB	6	4	20	0
Unclass	14	18	7	1

Supplementary Table 13. Sequencing coverage (HiFi) of the *piwi* gene regions and their flanking regions in Ajap.

Chr	Start	End	Gene	Depth1	Depth2	Depth3
LG01	32,963,671	32,966,993	AjapPiwi-3	39.54	41.68	37.46
LG01	32,993,488	32,996,810	AjapPiwi-2	28.54	32.72	21.58
LG01	33,065,401	33,069,291	AjapPiwi-1	30.15	30.89	33.21
LG01	181,230,388	181,233,932	AjapPiwi-4	42.35	52.53	39.36
LG01	191,537,225	191,541,409	AjapPiwi-5	24.24	26.55	26.63
LG01	202,860,209	202,864,258	AjapPiwi-6	38.74	43.88	26.26
LG02	28,106,720	28,110,169	AjapPiwi-7	39.13	41.98	36.3
LG02	28,157,577	28,161,170	AjapPiwi-8	45.1	43.35	45.98
LG04	6,256,680	6,259,738	AjapPiwi-9	38.08	39.18	35.87
LG04	6,471,251	6,474,299	AjapPiwi-10	32.82	33.17	31.62
LG04	6,485,027	6,488,075	AjapPiwi-11	29.18	31.16	27.6
LG04	6,498,803	6,501,851	AjapPiwi-12	27	27.5	30.82
LG04	6,512,578	6,515,626	AjapPiwi-13	36.61	31.4	37.11
LG04	6,544,431	6,547,479	AjapPiwi-14	31.89	29.38	32.48
LG04	6,590,758	6,593,806	AjapPiwi-15	29.08	37.82	12.25
LG04	6,599,389	6,602,436	AjapPiwi-16	6.29	23.85	12.03
LG04	6,612,623	6,615,674	AjapPiwi-18	45.66	12.76	41.9
LG04	6,621,255	6,624,306	AjapPiwi-17	34.26	47	27.02
LG04	17,846,788	17,850,830	AjapPiwi-19	50.77	54	50.25
LG04	147,830,520	147,833,819	AjapPiwi-20	43.9	38.94	23.42
LG04	147,837,696	147,840,995	AjapPiwi-21	22.66	39.75	8.48
LG04	147,848,052	147,851,351	AjapPiwi-22	9	12.39	24.64
LG04	147,855,227	147,858,526	AjapPiwi-23	26.14	10.74	52.52
LG04	147,862,403	147,865,702	AjapPiwi-24	57.79	28.56	62.1
LG04	147,869,579	147,872,878	AjapPiwi-25	59.92	55.5	53.39
LG04	147,876,755	147,880,054	AjapPiwi-26	51.46	61.32	46.01
LG04	147,883,931	147,887,230	AjapPiwi-27	49.04	51.69	38.34
LG04	147,940,960	147,944,233	AjapPiwi-28	44.34	47.16	43.56
LG04	147,951,630	147,954,903	AjapPiwi-29	47.77	42.45	43.28
LG04	148,940,714	148,944,083	AjapPiwi-30	42.08	40.43	40.99

*Depth1, average depth of the *piwi* region; Depth2, average depth of the 10 kb region upstream of the *piwi* gene; Depth3, average depth of the 10 kb region downstream of the *piwi* gene.

Supplementary Table 14. Sequencing coverage (ONT) of the *piwi* gene regions and their flanking regions in Ajap.

Chr	Start	End	Gene	Depth1	Depth2	Depth3
LG01	32,963,671	32,966,993	AjapPiwi-3	36.7	38.27	40.25
LG01	32,993,488	32,996,810	AjapPiwi-2	36.52	40.55	34.26
LG01	33,065,401	33,069,291	AjapPiwi-1	47.07	41.68	49.28
LG01	181,230,388	181,233,932	AjapPiwi-4	31.18	28.55	30.1
LG01	191,537,225	191,541,409	AjapPiwi-5	50.05	52.9	52.55
LG01	202,860,209	202,864,258	AjapPiwi-6	50.27	49.03	46.55
LG02	28,106,720	28,110,169	AjapPiwi-7	53.19	56.49	54.28
LG02	28,157,577	28,161,170	AjapPiwi-8	53.54	52.72	46.57
LG04	6,256,680	6,259,738	AjapPiwi-9	41.87	33.37	47.67
LG04	6,471,251	6,474,299	AjapPiwi-10	27.93	28.14	27.72
LG04	6,485,027	6,488,075	AjapPiwi-11	28.2	27.61	32.77
LG04	6,498,803	6,501,851	AjapPiwi-12	34.97	33.23	31.96
LG04	6,512,578	6,515,626	AjapPiwi-13	32.06	31.55	35.6
LG04	6,544,431	6,547,479	AjapPiwi-14	28.25	26.16	29.48
LG04	6,590,758	6,593,806	AjapPiwi-15	31.03	24.45	33.72
LG04	6,599,389	6,602,436	AjapPiwi-16	33.43	33.95	26.02
LG04	6,612,623	6,615,674	AjapPiwi-17	36.15	26.2	40.16
LG04	6,621,255	6,624,306	AjapPiwi-18	44.54	37.71	34.53
LG04	17,846,788	17,850,830	AjapPiwi-19	58.38	49.81	53.89
LG04	147,830,520	147,833,819	AjapPiwi-20	55.38	39.86	52.23
LG04	147,837,696	147,840,995	AjapPiwi-21	55.98	52.73	39.29
LG04	147,848,052	147,851,351	AjapPiwi-22	49.79	41.02	53.31
LG04	147,855,227	147,858,526	AjapPiwi-23	55.97	45.71	55.55
LG04	147,862,403	147,865,702	AjapPiwi-24	54.41	54.34	53.41
LG04	147,869,579	147,872,878	AjapPiwi-25	53.25	54.97	51.83
LG04	147,876,755	147,880,054	AjapPiwi-26	51.94	53.06	45.08
LG04	147,883,931	147,887,230	AjapPiwi-27	44.58	51.4	34.44
LG04	147,940,960	147,944,233	AjapPiwi-28	36.75	37.23	33.67
LG04	147,951,630	147,954,903	AjapPiwi-29	34.66	34.13	28.36
LG04	148,940,714	148,944,083	AjapPiwi-30	39.36	43.54	46.09

*Depth1, average depth of the *piwi* region; Depth2, average depth of the 10 kb region upstream of the *piwi* gene; Depth3, average depth of the 10 kb region downstream of the *piwi* gene.

Supplementary Table 15. Sequencing coverage (HiFi) of the *piwi* gene regions and their flanking regions in Aful.

Chr	Start	End	Gene	Depth1	Depth2	Depth3
LG01	13,045,160	13,047,783	AfulPiwi-3	41.49	41.89	49.07
LG01	31,444,243	31,448,091	AfulPiwi-2	43.27	43.28	36.35
LG01	181,876,863	181,879,945	AfulPiwi-1	56.09	53.94	44.4
LG02	155,058,260	155,061,837	AfulPiwi-4	43.97	44.36	35.52
LG02	155,080,112	155,083,583	AfulPiwi-5	38.38	39.91	33.88
LG04	5,022,939	5,026,001	AfulPiwi-11	32.64	35.39	30.41
LG04	5,066,989	5,070,051	AfulPiwi-10	30.33	31.56	34.12
LG04	5,142,439	5,145,497	AfulPiwi-9	34.26	29.62	32.48
LG04	5,157,407	5,160,465	AfulPiwi-8	23.09	30.23	24.7
LG04	5,198,171	5,201,229	AfulPiwi-7	24.43	26.86	35.12
LG04	5,206,745	5,209,803	AfulPiwi-6	39.25	27.97	41.87
LG04	20,403,115	20,405,566	AfulPiwi-12	33.34	29.88	37.41
LG04	21,317,817	21,320,967	AfulPiwi-13	46.42	40.96	48.13
LG04	21,338,440	21,341,590	AfulPiwi-14	49.63	47.09	53.53
LG04	21,391,399	21,394,550	AfulPiwi-15	41.77	42.17	49.18
LG04	149,774,767	149,778,831	AfulPiwi-16	36.08	33.68	38.93

*Depth1, average depth of the *piwi* region; Depth2, average depth of the 10 kb region upstream of the *piwi* gene; Depth3, average depth of the 10 kb region downstream of the *piwi* gene.

Supplementary Table 16. Sequencing coverage (ONT) of the *piwi* gene regions and their flanking regions in Aful.

Chr	Start	End	Gene	Depth1	Depth2	Depth3
LG01	13,045,160	13,047,783	AfulPiwi-3	60.9	62.62	56.86
LG01	31,444,243	31,448,091	AfulPiwi-2	42.23	36.45	35.61
LG01	181,876,863	181,879,945	AfulPiwi-1	47.95	47.86	46.77
LG02	155,058,260	155,061,837	AfulPiwi-4	40.31	41.73	39.63
LG02	155,080,112	155,083,583	AfulPiwi-5	35.92	37.35	29.3
LG04	5,022,939	5,026,001	AfulPiwi-11	16.78	17.85	16.24
LG04	5,066,989	5,070,051	AfulPiwi-10	20.96	17.79	23.31
LG04	5,142,439	5,145,497	AfulPiwi-9	19.71	22.09	20.71
LG04	5,157,407	5,160,465	AfulPiwi-8	19.73	20.62	20.42
LG04	5,198,171	5,201,229	AfulPiwi-7	17.29	17.8	17.23
LG04	5,206,745	5,209,803	AfulPiwi-6	16.78	17.19	18.4
LG04	20,403,115	20,405,566	AfulPiwi-12	56.78	54.12	57.32
LG04	21,317,817	21,320,967	AfulPiwi-13	53.34	54.64	52.79
LG04	21,338,440	21,341,590	AfulPiwi-14	52.77	49.1	52.68
LG04	21,391,399	21,394,550	AfulPiwi-15	50.48	52.14	46.79
LG04	149,774,767	149,778,831	AfulPiwi-16	50.89	49.09	49.76

*Depth1, average depth of the *piwi* region; Depth2, average depth of the 10 kb region upstream of the *piwi* gene; Depth3, average depth of the 10 kb region downstream of the *piwi* gene.

Supplementary Table 17. Selection analysis of the *piwi* gene family.

Hypothesis	lnL	Branches	Omega (ω)	LRT	*P*-value
H0	-24790.7069	All the branches	0.057		
H_alternative	-24741.44848	Hym-conserved	0.050	98.51685	0.00E+00
		Cha-specific	0.115		
		Rest of the branches	0.040		

*P value was calculated using the likelihood ratio test (LRT).

Supplementary Table 18. Small RNA read sizes mapping to the *A. japonicus* genome (Reads per million)

Length	piRNA	miRNA	4ncRNA (rRNA, tRNA, snRNA, snoRNA)	Other smRNA	siRNA
18	0	12885	88397	52965	0
19	0	21073	134805	77430	0
20	0	158562	173806	125859	0
21	0	1288505	134083	0	231373
22	0	3213090	126662	0	1221650
23	0	2799872	119417	0	437370
24	570546	288162	111730	0	0
25	1191964	33477	139740	0	0
26	2198463	6121	144291	0	0
27	3894775	7283	121726	0	0
28	5385658	2012	145185	0	0
29	4155701	835	164050	0	0
30	1681660	1214	80848	0	0
31	468022	328	58813	0	0
32	180541	33	63907	0	0
33	103263	56	57654	0	0
34	26593	16	38565	0	0
35	13840	43	18317	0	0

Supplementary Table 19. Small RNA read sizes mapping to the *A. fulloi* genome (Reads per million)

length	piRNA	miRNA	4ncRNA (rRNA, tRNA, snRNA, snoRNA)	Other smRNA	siRNA
18	0	12845	87018	58042	0
19	0	22569	119709	72647	0
20	0	211632	165864	125245	0
21	0	1597189	121248	0	209320
22	0	3194975	107463	0	1186414
23	0	2705981	104264	0	377588
24	493367	318729	98085	0	0
25	1049335	36010	122877	0	0

26	2005024	4100	127436	0	0
27	3477107	2002	130811	0	0
28	4797025	978	150329	0	0
29	3805476	541	142046	0	0
30	1219547	193	81818	0	0
31	466901	81	53909	0	0
32	205959	62	66463	0	0
33	153310	33	62347	0	0
34	41997	35	60618	0	0
35	28302	86	30905	0	0

Supplementary Table 20. 2*2 contingency table for Chi-square test to assess the significance of the coincidence of the gene expression shift in venom gland and NRER.

	With NRER	Without NRER
With large expression change	22	2
Without obvious expression change	28	23

*The chi-square statistic is 9.9265. The p-value is 0.001629.

Supplementary Table 21. 2*2 contingency table for Chi-square test to assess the significance of the coincidence of the module conservation and venom genes.

Ajap	Module shift	Module conservation
Venom	9	131
non-Venom	135	79

The chi-square statistic is 112.5766. The p-value is 2.67e-26.

Aful	Module shift	Module conservation
Venom	7	113
non-Venom	86	96

The chi-square statistic is 58.2154. The p-value is 2.35e-14.

Supplementary Table 22. Species used for comparative genomics analyses.

Species	Data sources	Assembly accession
Athalia rosae	NCBI	GCF_000344095.2
Orussus abietinus	NCBI	GCF_000612105.2
Diadromus collaris	NCBI	GCA_009394715.1
Cotesia chilonis	InsectBase2.0	IBG_00206
Belonocnema treatae	NCBI	GCF_010883055.1
Telenomus remus	NCBI	GCA_020615435.1
Trichogramma pretiosum	NCBI	GCF_000599845.2
Copidosoma floridanum	NCBI	GCF_000648655.2
Ceratosolen solmsi	NCBI	GCF_000503995.1
Pteromalus puparum	InsectBase2.0	IBG_00672
Nasonia vitripennis	NCBI	GCF_009193385.2
Gonatopus flavifemur	InsectBase2.0	IBG_00409

Polistes dominula	NCBI	GCF_001465965.1
Ooceraea biroi	NCBI	GCF_003672135.1
Atta cephalotes	NCBI	GCA_000143395.3
Apis mellifera	NCBI	GCF_003254395.2
Bombus terrestris	NCBI	GCF_000214255.1
Anastatus japonicus	this study	-
Anastatus fulloi	this study	-
