# Supplementary material for: Genomic signatures associated with maintenance of genome stability and venom turnover in two parasitoid wasps
Source: Nat Commun. 2022 Oct 27;13:6417. doi: 10.1038/s41467-022-34202-y (PMC9613689; doi:10.1038/s41467-022-34202-y)
Supplement: Supplementary file 3 — Reporting Summary [file 41467_2022_34202_MOESM3_ESM.pdf]

nature portfolio

Corresponding author(s): Gongyin Ye, Dunsong Li

Last updated by author(s): Oct 8, 2022

Reporting Summary

Nature Portfolio wishes to improve the reproducibility of the work that we publish. This form provides structure for consistency and transparency in reporting. For further information on Nature Portfolio policies, see our Editorial Policies and the Editorial Policy Checklist.

Statistics

For all statistical analyses, confirm that the following items are present in the figure legend, table legend, main text, or Methods section.

n/a	Confirmed	
☐	☒	The exact sample size (*n*) for each experimental group/condition, given as a discrete number and unit of measurement
☐	☒	A statement on whether measurements were taken from distinct samples or whether the same sample was measured repeatedly
☐	☒	The statistical test(s) used AND whether they are one- or two-sided<br>*Only common tests should be described solely by name; describe more complex techniques in the Methods section.*
☒	☐	A description of all covariates tested
☐	☒	A description of any assumptions or corrections, such as tests of normality and adjustment for multiple comparisons
☐	☒	A full description of the statistical parameters including central tendency (e.g. means) or other basic estimates (e.g. regression coefficient) AND variation (e.g. standard deviation) or associated estimates of uncertainty (e.g. confidence intervals)
☐	☒	For null hypothesis testing, the test statistic (e.g. *F*, *t*, *r*) with confidence intervals, effect sizes, degrees of freedom and *P* value noted<br>*Give P values as exact values whenever suitable.*
☒	☐	For Bayesian analysis, information on the choice of priors and Markov chain Monte Carlo settings
☒	☐	For hierarchical and complex designs, identification of the appropriate level for tests and full reporting of outcomes
☐	☒	Estimates of effect sizes (e.g. Cohen's *d*, Pearson's *r*), indicating how they were calculated

Our web collection on statistics for biologists contains articles on many of the points above.

Software and code

Policy information about availability of computer code

Data collection	PacBio HiFi sequencing was performed on the PacBio Sequel II platform. Oxford Nanopore ultralong-read genome sequencing was performed on the Nanopore PromethION sequencer. Short-read genome sequencing and RNA sequencing were performed on the Illumina Hiseq Xten platform. Hi-C data were sequenced using the Illumina Novaseq platform. BGISEQ-500 platform was used to sequence small RNA libraries.
Data analysis	jellyfish (2.2.10), findGSE (1.94), Fastp (0.20.0), ccs (6.0.0), Hifiasm (0.12), Nextpolish (1.0.5), HiC-Pro (3.1.0), LACHESIS, bowtie2 (2.2.3), BUSCO (5), BWA (0.7.17), minimap2 (2.20), Samtools (1.16), EDTA (1.9.9), RepeatMasker (4.0.7), LTR_FINDER_parallel (1.1), LTR_retriever (2.9.0), HISAT2 (2.2.1), StringTie (2.1.0), TransDecoder (5.4.0), GenomeThreader (1.7.1), BRAKER2 (2.1.6), GeneMark-EP+ (4.69), AUGUSTUS (3.1), EVidenceModeler (1.1.1), BLAST+ (2.13.0), HMMscan (3.3.2), MAFFT (7.478), DISTMAT (implemented in EMBOSS v6.6.0.0), r8s (1.8.1), TEsorter (1.3.0), trimAl (1.2), Fasttree (2.1), Orthofinder (2.1), IQ-TREE (2.0), ModelFinder (implemented in IQ-TREE v2.0), CAFE (4.2.1), Exonerate (2.4.0), InsectOR, Fgenesh+ (http://www.softberry.com/berry.phtml?topic=fgenes_plus&group=programs&subgroup=gfs), Paml (4.9), bowtie (1.3.1), Trimmomatic (0.38), BEDtools (2.30.0), proTRAC (2.4.3), RSEM (1.3.3), MaxQuant (2.0.3.1), LASTZ (1.04.03), WGCNA (1.66), GOATOOLS (1.0.6), IGV (2.9.4). All computational codes used in this study are available at https://github.com/yexinhai/Anastatus_genome_project103 and archived at https://10.5281/zenodo.7155373.

For manuscripts utilizing custom algorithms or software that are central to the research but not yet described in published literature, software must be made available to editors and reviewers. We strongly encourage code deposition in a community repository (e.g. GitHub). See the Nature Portfolio guidelines for submitting code & software for further information.

Data

Policy information about availability of data

All manuscripts must include a data availability statement. This statement should provide the following information, where applicable:

- Accession codes, unique identifiers, or web links for publicly available datasets
- A description of any restrictions on data availability
- For clinical datasets or third party data, please ensure that the statement adheres to our policy

The sequencing data generated in this study have been deposited in the National Genomics Data Center under accession number PRJCA008911[https://ngdc.cncb.ac.cn/bioproject/browse/PRJCA008911]. The genome assembly data have been deposited in the Genome Warehouse under accession number GWHBKAI00000000 and GWHBJYV00000000. The raw data of PacBio (CRA007569[https://ngdc.cncb.ac.cn/gsa/browse/CRA007569] and CRA007570[https://ngdc.cncb.ac.cn/gsa/browse/CRA007570]), Illumina (CRA006534[https://ngdc.cncb.ac.cn/gsa/browse/CRA006534] and CRA006538[https://ngdc.cncb.ac.cn/gsa/browse/CRA006538]), Hi-C (CRA007796[https://ngdc.cncb.ac.cn/gsa/browse/CRA007796] and CRA007800[https://ngdc.cncb.ac.cn/gsa/browse/CRA007800]), ONT (CRA007785[https://ngdc.cncb.ac.cn/gsa/browse/CRA007785] and CRA007786[https://ngdc.cncb.ac.cn/gsa/browse/CRA007786]), small RNA-seq (CRA007801 [https://ngdc.cncb.ac.cn/gsa/browse/CRA007801] and CRA007802[https://ngdc.cncb.ac.cn/gsa/browse/CRA007802]) and RNA-seq (CRA006651[https://ngdc.cncb.ac.cn/gsa/browse/CRA006651] and CRA006642[https://ngdc.cncb.ac.cn/gsa/browse/CRA006642]) are available in the Genome Sequence Archive. Other public datasets used in this study include insecta_odb10 (https://busco-data.ezlab.org/v5/data/lineages/insecta_odb10.2020-09-10.tar.gz), all known miRNA hairpin precursors in miRbase (https://mirbase.org/ftp/CURRENT/hairpin.fa.gz), OrthoDB (https://v101.orthodb.org/download/odb10v1_all_fasta.tab.gz). Source data are provided with this paper.

Human research participants

Policy information about studies involving human research participants and Sex and Gender in Research.

Reporting on sex and gender	N/A
Population characteristics	N/A
Recruitment	N/A
Ethics oversight	N/A

Note that full information on the approval of the study protocol must also be provided in the manuscript.

Field-specific reporting

Please select the one below that is the best fit for your research. If you are not sure, read the appropriate sections before making your selection.

- [x] Life sciences
- [ ] Behavioural & social sciences
- [ ] Ecological, evolutionary & environmental sciences

For a reference copy of the document with all sections, see nature.com/documents/nr-reporting-summary-flat.pdf

Life sciences study design

All studies must disclose on these points even when the disclosure is negative.

Sample size	The genomic DNA of each species was extracted from about 50 haploid male pupae. RNA-seq libraries (insert size of 250 bp) were prepared from 2nd instar larva (10 individuals), 3nd instar larva (10 individuals), 4th instar larva (10 individuals), female pupa (10 individuals), male pupa (10 individuals), female adult (1-day-old, 10 individuals), male adult (1-day-old, 10 individuals), venom gland (3-day-old female adult, 100 individuals), and carcass (3-day-old female adult, remove venom gland, 10 individuals). Small RNA sequencing libraries were constructed from 50 adult females, for A. japonicus and A. fulloi, respectively. 100 venom reservoirs of each species were isolated for LC-MS/MS analysis. Our prior experience demonstrates that our sample sizes are sufficient for related DNA or RNA sequencing.
Data exclusions	No data were excluded for analysis.
Replication	We include at least 3 biological replicates to compute our p value. All replicates were successful.
Randomization	Randomization procedures are not applicable to this study since we are not studying on population.
Blinding	Blinding is not relevant to our study as we don't have treatment or control groups.

Reporting for specific materials, systems and methods

We require information from authors about some types of materials, experimental systems and methods used in many studies. Here, indicate whether each material, system or method listed is relevant to your study. If you are not sure if a list item applies to your research, read the appropriate section before selecting a response.

Materials & experimental systems

n/a	Involved in the study
☒	☐ Antibodies
☒	☐ Eukaryotic cell lines
☒	☐ Palaeontology and archaeology
☐	☒ Animals and other organisms
☒	☐ Clinical data
☒	☐ Dual use research of concern

Methods

n/a	Involved in the study
☒	☐ ChIP-seq
☒	☐ Flow cytometry
☒	☐ MRI-based neuroimaging

Animals and other research organisms

Policy information about studies involving animals; ARRIVE guidelines recommended for reporting animal research, and Sex and Gender in Research

Laboratory animals	We used two Anastatus wasps, A. japonicus (strain: zju) and A. fulloi (strain: zju). The venom glands and venom reservoirs were collected from 3-day-old female wasps. The genomic DNA of each species was extracted from male pupae. RNA-seq libraries were prepared from 2nd instar larva, 3nd instar larva, 4th instar larva, female pupa, male pupa, 1-day-old female adult, 1-day-old male adult.
Wild animals	The study did not involve wild animals.
Reporting on sex	The venom glands and venom reservoirs were collected from female wasps since the venom organ was specific to female wasps.
Field-collected samples	The study did not involve sample collected from the wild.
Ethics oversight	No ethical approval or guidance was required.

Note that full information on the approval of the study protocol must also be provided in the manuscript.
