# Supplementary material for: Genomic signatures associated with maintenance of genome stability and venom turnover in two parasitoid wasps
Source: Nat Commun. 2022 Oct 27;13:6417. doi: 10.1038/s41467-022-34202-y (PMC9613689; doi:10.1038/s41467-022-34202-y)
Supplement: Supplementary file 4 — Description of Additional Supplementary Files [file 41467_2022_34202_MOESM4_ESM.pdf]

Description of Additional Supplementary Files

File Name: Supplementary Data 1
Description: Structural variant (larger than 10Kb) between the two *Anastatus* genomes.

File Name: Supplementary Data 2
Description: TE content (%) of selected hymenopteran genomes (based on EDTA results).

File Name: Supplementary Data 3
Description: Intact LTR-RTs in *Anastatus japonicas* genome.

File Name: Supplementary Data 4
Description: Intact LTR-RTs in *Anastatus fulloi* genome.

File Name: Supplementary Data 5
Description: Rate of gene gain and loss along the hymenopteran phylogeny.

File Name: Supplementary Data 6
Description: Go enrichment of expanded genes in the common ancestor of the two *Anastatus* wasps (biological process, FDR-adjusted $p < 0.05$)

File Name: Supplementary Data 7
Description: Go enrichment of expanded genes in *A. japonicus* (biological process, FDR-adjusted $p < 0.05$).

File Name: Supplementary Data 8
Description: Go enrichment of expanded genes in *A. fulloi* (biological process, FDR-adjusted $p < 0.05$).

File Name: Supplementary Data 9
Description: Summary of genome sizes of the 193 hymenopteran species.

File Name: Supplementary Data 10
Description: LTR-RT content (%) of selected hymenopteran genomes (based on EDTA resutls).

File Name: Supplementary Data 11
Description: Gene length comparison among hymenopterans (single copy genes).

File Name: Supplementary Data 12
Description: Intact ONT long reads cover the *Piwi* gene regions in *A. japonicus*.

File Name: Supplementary Data 13
Description: Intact ONT long reads cover the *Piwi* gene regions in *A. fulloi*.

File Name: Supplementary Data 14
Description: Predicted piRNA clusters in *A. japonicus*.

File Name: Supplementary Data 15
Description: Predicted piRNA clusters in *A. fulloi*.

File Name: Supplementary Data 16
Description: Venom genes in *A. japonicus*.

File Name: Supplementary Data 17
Description: Venom genes in *A. fulloi*.

File Name: Supplementary Data 18
Description: Comparative analysis of venom genes among 38 parasitoid wasps

File Name: Supplementary Data 19
Description: Comparative analysis of venom genes between the two *Anastatus* wasps.

File Name: Supplementary Data 20
Description: Summary of the venom evolution models of the two *Anastatus* wasps.

File Name: Supplementary Data 21
Description: Summary of the rapid evolving venom genes of the two *Anastatus* wasps.

File Name: Supplementary Data 22
Description: 202 one-to-one orthologous genes used for venom gene expression analysis.

File Name: Supplementary Data 23
Description: Analysis of the gene expression shift in venom gland and NRER.

File Name: Supplementary Data 24
Description: 38 gene coexpression modules in *A. japonicus* (red module represents the venom-related network module).

File Name: Supplementary Data 25
Description: 37 gene coexpression modules in *A. fulloi* (purple module represents the venom-related network module).

File Name: Supplementary Data 26
Description: Module assignment of the venom-related network module member of *A. japonicus* in a dataset without venom gland samples.

File Name: Supplementary Data 27
Description: Module assignment of the venom-related network module member of *A. fulloi* in a dataset without venom gland samples.

File Name: Supplementary Data 28
Description: Go enrichment of the non-venom genes which are coexpressed with venom genes of *A. japonicus* (biological process, FDR-adjusted $p < 0.05$).

File Name: Supplementary Data 29
Description: Module conservation and module shift of ortholog pairs between the two *Anastatus* species (*A. japonicus* as reference).

File Name: Supplementary Data 30
Description: Module conservation and module shift of ortholog pairs between the two *Anastatus* species (*A. fulloi* as reference).

File Name: Supplementary Data 31
Description: Ortholog analysis of the non-venom genes in VRMs.

File Name: Supplementary Data 32
Description: Primers used in this study.

File Name: Supplementary Data 33
Description: Statistical test analyses for qPCR results of *Piwi* genes.
